# Unsaturated Phosphine Oxides for Modular Antibody Rebridging and Single Reagent Peptide‐Cyclization‐Bioconjugation

**DOI:** 10.1002/anie.202508656

**Published:** 2025-08-08

**Authors:** Christian E. Stieger, Alastair J. McMillan, Mark A. R. de Geus, Jan Vincent V. Arafiles, Luise Franz, Christian P. R. Hackenberger

**Affiliations:** ^1^ Leibniz‐Forschungsinstitut für Molekulare Pharmakologie, im Forschungsverbund Berlin e.V. (FMP), Chemical Biology Department Campus Berlin‐Buch Robert‐Roessle‐Strasse 10 13125 Berlin Germany; ^2^ Department of Chemistry Humboldt‐Universität zu Berlin Brook‐Taylor‐Strasse 2 12489 Berlin Germany; ^3^ Freie Universität Berlin, Institute of Chemistry and Biochemistry Arnimallee 20 14195 Berlin Germany

**Keywords:** Antibody rebridging, Antibody‐drug conjugates, Bioconjugation, Click chemistry, Peptide cyclization

## Abstract

Achieving modular, selective and homogeneous protein modifications is of utmost importance for the design of next generation biopharmaceuticals, especially in the context of antibody‐drug conjugates (ADCs). Here, we introduce unsaturated phosphine oxides as versatile triple‐reactive reagents, allowing orthogonal chemoselective bioconjugation schemes. Starting from triethynyl‐phosphine oxide, a variety of functionalized diethynyl‐triazolyl‐phosphine oxides (DTPOs) could be accessed by using Cu^I^‐catalyzed azide‐alkyne cycloaddition (CuAAC). We showcase DTPO‐reagents in the fast and selective generation of various highly stable antibody‐conjugates via antibody disulfide rebridging. A highlight from this methodology is the synthesis of a DAR 4 ADC following a modular 2‐step strategy using bioorthogonal tetrazine‐labeling with bicyclo‐[6.1.0]non‐4‐yne (BCN) or *trans*‐cyclooctene (TCO) containing payloads. Notably, the DTPO‐rebridged ADC exhibited potent cytotoxicity against Her2^+^ cancer cells. Moreover, we utilize triethynyl‐phosphine oxide to obtain ethynyl‐ditriazolyl‐phosphine oxides (EDPOs) which enable a unique, single‐reagent peptide‐cyclization‐bioconjugation protocol resulting in functional cyclic peptide‐protein conjugates. Overall, our work provides versatile and powerful chemoselective modalities for the controlled modification of antibodies, peptide‐cyclization and peptide‐protein conjugation, expanding the toolkit for chemical biology and therapeutic development.

## Graphical abstract

We report the modular synthesis of unsaturated P5 reagents starting from triethynyl‐phosphine oxide via CuAAC. Diethynyl‐triazolyl‐phosphine oxides enable rapid, selective and stable antibody disulfide rebridging, exemplified by the generation of a potent and selective ADC. In addition, triethynyl‐phosphine oxide enables a unique single reagent peptide‐cyclization‐bioconjugation protocol to access functional cyclic‐peptide protein conjugates.

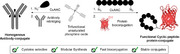

## Introduction

**Scheme 1:**
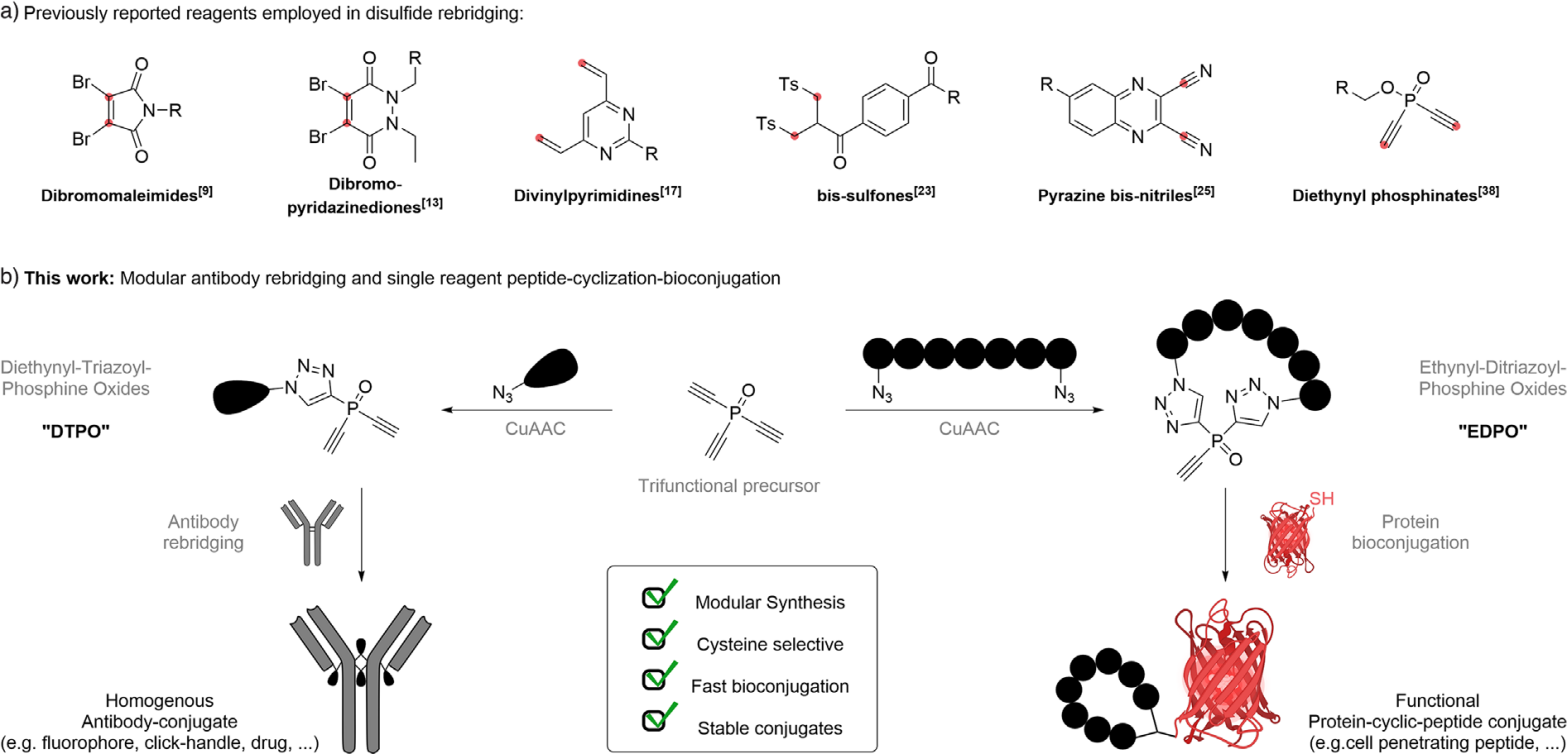
a) Chemical structure of previously reported bis‐reactive electrophiles employed in disulfide rebridging.^[^ ^9^, ^13^, ^17^, ^23^, ^25^, ^38^ ^]^ b) This work: Modularly accessible reagents for fast and chemoselective antibody rebridging and for single reagent peptide‐cyclization‐bioconjugation.

The ability to selectively and precisely modify biomolecules is a transformative endeavor in the chemical and pharmaceutical life sciences. Over the recent years, significant progress has been made in the development of various methodologies, particularly for cysteine‐selective peptide and protein modification.^[^ ^1^, ^2^, ^3^, ^4^ ^]^ One main driver for these bioconjugation techniques is the field of antibody‐drug conjugates (ADCs), since antibodies can be functionalized via stochastic modification of the reduced interchain disulfides. Nevertheless, this strategy often results in a heterogeneous mixture of antibody‐conjugates, leading to unpredictable and inconsistent pharmacological profiles.^[^ ^5^, ^6^ ^]^ Even though several approaches to produce homogeneous ADCs have been developed,^[^ ^7^ ^]^ complex genetic engineering often limits broad accessibility. Another attractive approach, known as disulfide rebridging, involves the reduction of cystines followed by a subsequent reaction with a bis‐reactive electrophile.^[^ ^8^ ^]^ Several of these reagents were recently developed, including modified‐maleimides,^[^ ^9^, ^10^, ^11^, ^12^ ^]^ dihalopyridazinediones,^[^ ^13^, ^14^, ^15^, ^16^ ^]^ divinyl‐pyrimidines,^[^ ^17^, ^18^, ^19^, ^20^, ^21^, ^22^ ^]^ bis‐sulfones^[^ ^23^, ^24^ ^]^ among others^[^ ^25^, ^26^, ^27^, ^28^, ^29^, ^30^ ^]^ (Scheme 1a). This approach is particularly appealing when it comes to the modification of native immunoglobulin G antibodies (IgGs). IgG1 and IgG4 both contain four readily accessible inter‐chain disulfide bridges, which allows the precise loading of four cargo molecules, one on each rebridging reagent, onto the antibody. Moreover, studies have demonstrated that a covalent non‐reductive linkage between the antibody chains has the potential to improve the antibody's thermal stability.^[^ ^16^ ^]^ Because of this, rebridging is considered a promising strategy for enhancing antibody properties and developing ADCs with improved pharmacokinetics.^[^ ^12^, ^14^, ^19^, ^31^ ^]^ In addition to ADCs, protein‐peptide or peptide‐peptide conjugates are of great interest in chemical biology. In this regard, cyclic peptides are of particular interest because of their superior properties including enhanced cell‐permeability, improved binding affinity and resistance to proteolytic degradation.^[^ ^32^, ^33^, ^34^, ^35^ ^]^ To achieve both cyclization and electrophile incorporation, complex protecting group strategies or multi‐step reactions have to be employed to obtain cyclic peptides that are accessible for subsequent protein‐bioconjugation.^[^ ^36^, ^37^ ^]^

In a previous study, our group has reported on the use of diethynyl‐phosphinates (DPs) as cysteine‐selective rebridging reagents that produce homogeneous and highly stable antibody conjugates (Scheme 1a).^[^ ^38^ ^]^ Following this report, we discovered that the derivatization of one alkyne‐substituent with Copper^I^‐catalyzed azide‐alkyne cycloaddition (CuAAC) delivers ethynyl‐triazolyl‐phosphinates (ETPs), which offer highly increased cysteine reactivity compared to previously developed P^V^‐electrophiles, while remaining highly selective.^[^ ^39^ ^]^ Building upon these two recent discoveries, we aimed to create a modularly accessible disulfide‐rebridging platform based on unsaturated phosphine oxides that operates with excellent efficiency and selectivity (Scheme 1b, left panel). We have now identified triazolyl‐phosphine oxides containing two ethynyl‐substituents (**D**iethynyl‐**T**riazolyl‐**P**hosphine **O**xide, DTPOs) as optimally functionalized reagents for antibody rebridging, offering superior reactivity, efficiency and accessibility, which ultimately allow the straightforward site‐selective and stable generation of various antibody‐conjugates including a biologically active ADC. Moreover, we discovered that the corresponding ditriazolyl‐phosphine oxides containing a single terminal alkyne (**E**thynyl‐**D**itriazolyl‐**P**hosphine **O**xides, EDPOs), represent a new class of cysteine directed electrophiles. We leveraged this finding to obtain cyclic‐peptides that are directly usable in protein‐bioconjugation and furnished a cell‐permeable and fluorescent peptide‐protein conjugate (Scheme 1b, right panel). To the best of our knowledge, this work represents the first reagent that enables chemoselective peptide cyclization with products that contain a bioconjugation‐compatible, cysteine‐reactive handle which can directly react with native proteins. Comparable methods stem from the development of covalent inhibitors, which employ a very weak electrophile that will only react in a proximity‐induced manner.^[^ ^40^, ^41^ ^]^

## Results and Discussion

**Figure 1:**
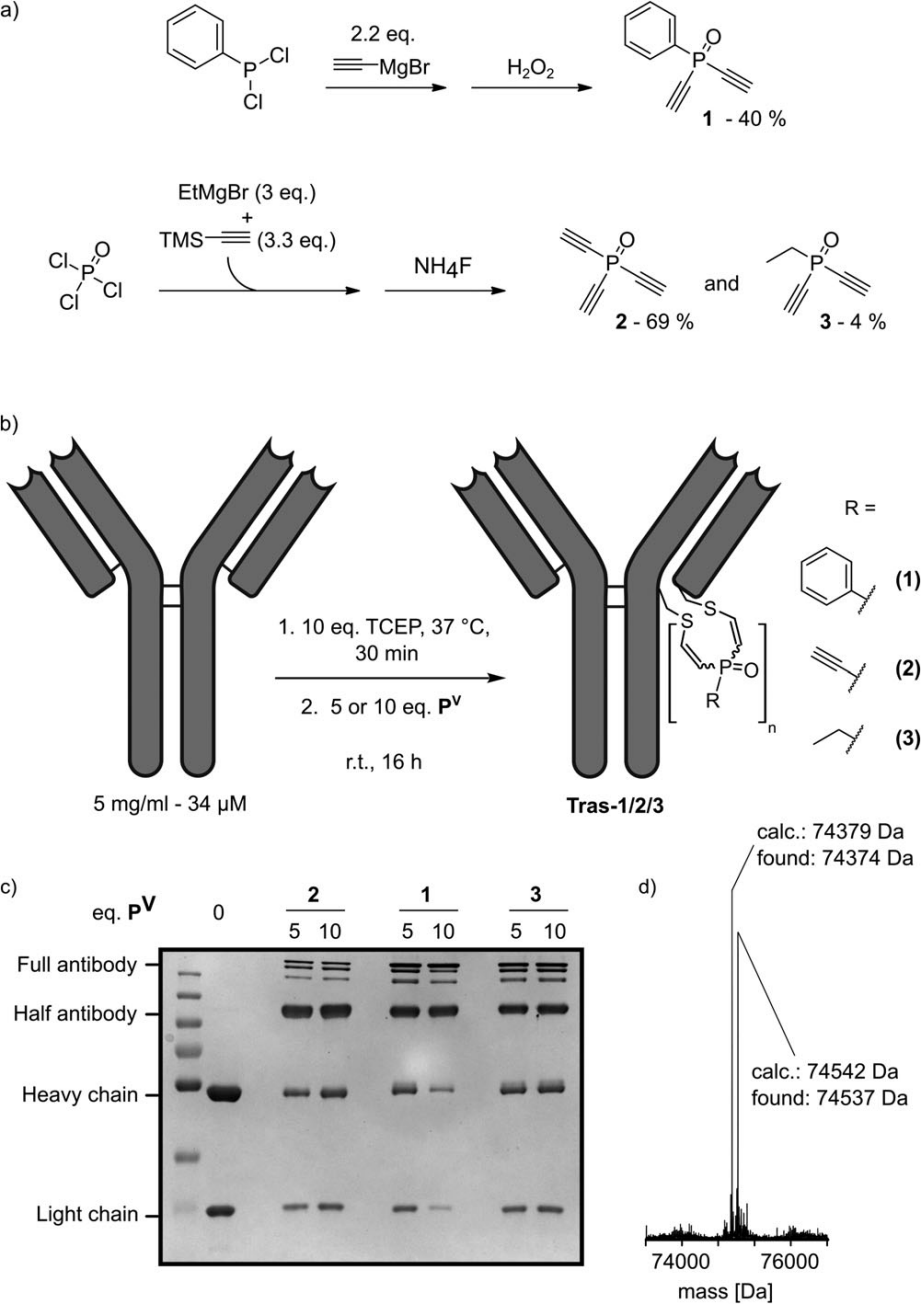
a) Synthetic procedure to obtain diethynyl‐phosphine oxides **1**–**3**. b) Schematic representation of the antibody rebridging reaction. c) SDS‐PAGE analysis of Trastuzumab rebridged with 5 or 10 eq. of the corresponding phosphine oxide **1**–**3** overnight. For uncropped gel see ESI 5.1.1. d) Intact‐protein mass spectrum of Trastuzumab, rebridged with 10 eq. phosphine oxide **1** overnight (**Tras‐1**).

Since our previous studies demonstrated that the P‐substitution pattern greatly influences the reactivity of unsaturated P^V^‐electrophiles,^[^ ^42^ ^]^ we started our investigation by exploring the reactivity of different diethynyl‐containing phosphine oxides containing sp, sp^2^ or sp^3^‐hybridized substituents. As a first example, we obtained the sp^2^‐hybridized phenyl‐substituted diethynyl‐phosphine oxide (**1**) from commercially available starting materials in 40% yield over two steps. Moreover, the sp‐hybridized triethynyl‐phosphine oxide (**2**) was synthesized as described in the literature.^[^ ^43^ ^]^ Conveniently, we obtained diethynyl‐ethyl‐phosphine oxide (**3**) as a minor side product delivering a sp^3^‐hybridized model compound (Figure 1a). With these compounds in hand, we tested their ability to rebridge the monoclonal IgG1 antibody Trastuzumab, a FDA‐approved monoclonal antibody that is commonly used in the development of ADCs for the treatment of Her2^+^ breast cancer (Figure 1b).^[^ ^44^ ^]^ We treated the reduced antibody (5 mg ml^−1^, 10 eq. TCEP, 37 °C, 30 min) with 5 or 10 eq. of the corresponding phosphorus electrophiles **1**‐**3** and let the reaction proceed overnight before analysis by SDS‐PAGE. Gel electrophoresis indicated significant rebridging yields for all three compounds with **1** showing the best result with > 70% conversion (by gel densitometry) corresponding to rebridged antibody species (Figure 1c). Moreover, we were able to identify the expected half antibody‐species obtained for **Tras‐1** via intact protein MS (Figure 1d, ESI 3.1.2). To our surprise, for **Tras‐2** and **Tras‐3** we observed partial hydrolysis of the reagents (see ESI 3.1.3–3.1.4). This finding suggests that sp^2^‐hybridized diethynyl‐phosphine oxides might be suitable candidates to obtain efficient rebridging reagents.

Motivated by these promising results, we investigated how to incorporate functional modules most easily into the sp^2^‐hybridzed diethynyl‐phosphine oxide core structure to generate functional antibody conjugates. Although aryl‐phosphine oxides bearing functional groups for subsequent derivatization are synthetically accessible,^[^ ^45^, ^46^ ^]^ we were seeking for a more modular chemoselective approach to obtain sp^2^‐substituted phosphine oxide‐rebridging reagents. Since phosphine oxide **2** displays three electrophilic ethynyl‐substituents, one could imagine a first functionalization with a thiol‐containing nucleophile to obtain a sp^2^‐hybridized diethynyl‐phosphine oxide (Figure S1). While we observed sufficient reactivity for the first and second addition of thiols to **2**, the third thiol addition proceeds extremely slow and did not reach completion (data not shown). This observation is in agreement with the previously reported reduced reactivity of thiovinyl‐ethynyl‐phosphinates compared to diethynyl‐phosphinates.^[^ ^39^ ^]^ Consequently, we concluded that diethynyl‐thiovinyl‐phosphine oxides would not provide favorable rebridging results.

Recently, we have reported that functionalization of one ethynyl‐substituent in diethynyl phosphinates into a triazole using CuAAC greatly enhances the reaction rates of thiol additions to the remaining ethynyl‐moiety.^[^ ^39^ ^]^ We found that the triazolyl group attached to phosphorus leads to improved thiol‐reactivity due to electron‐withdrawing effects and hyperconjugation.^[^ ^39^ ^]^ Therefore, we proposed to adopt this concept to triethynyl‐phosphine oxide (**2**) by generating electrophilic DTPOs using CuAAC. We reacted compound **2** with fluorescein azide (5 eq. phosphine oxide, 20 mol% CuBr)^[^ ^39^ ^]^ in a 1:1 mixture of PBS and acetonitrile. Already after 10 minutes, full consumption of the starting material was observed by UPLC‐MS. However, alongside the desired diethynyl‐triazolyl‐phosphinate, the double and triple‐cycloaddition products (**4a** & **4b**) were formed as side products (see ESI 4.2.1). This byproduct formation could be mostly abolished by doubling the equivalents of **2**, yielding DTPO **4** in 51% yield after HPLC purification.

**Figure 2:**
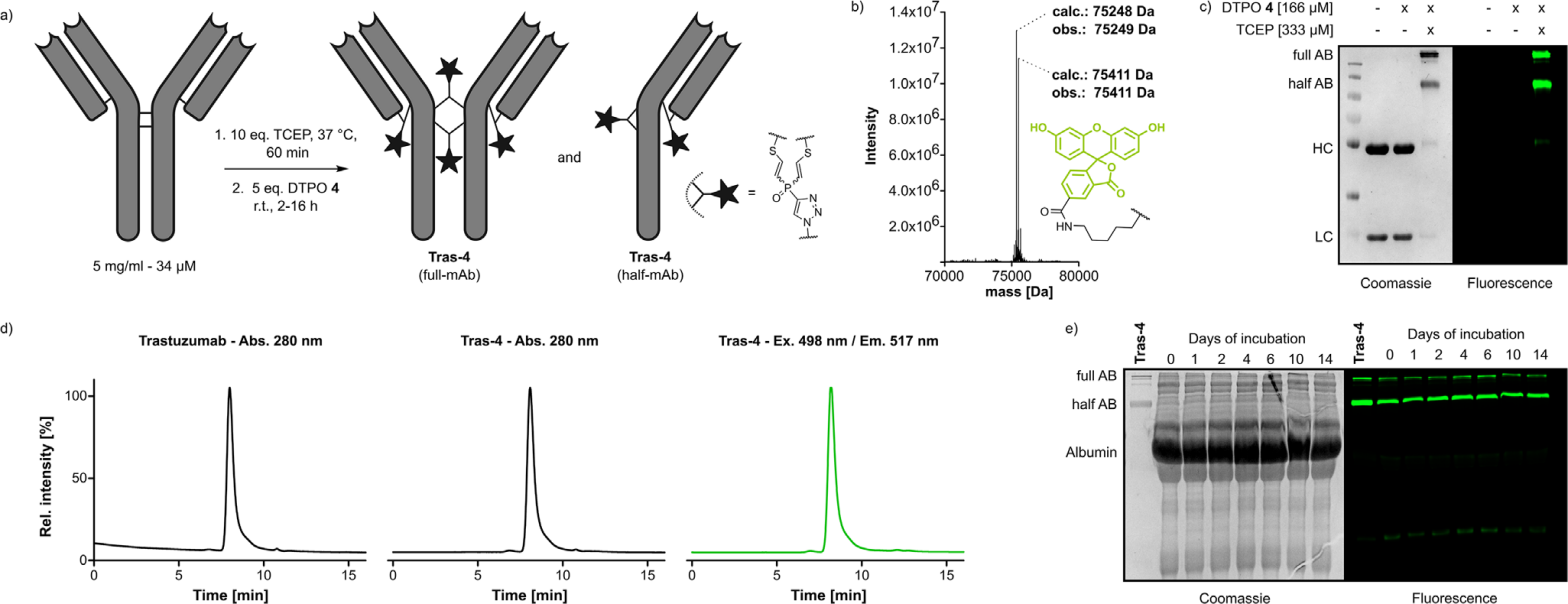
a) Schematic representation of the rebridging reaction of Trastuzumab and DTPO **4**. b) Deconvoluted intact protein MS spectrum of the crude reaction mixture after overnight incubation, indicating complete formation of rebridged antibody species (**Tras‐4**). c) Reducing SDS‐PAGE analysis of Trastuzumab rebridged with 5 eq. DTPO **4** overnight with or without prior reduction with TCEP. For uncropped gel see ESI 5.1.2. d) Size‐exclusion chromatography analysis of Trastuzumab and **Tras‐4**. e) Stability testing of **Tras‐4** incubated in human serum by SDS‐PAGE. For uncropped gel see ESI 5.1.3.

Subsequently, we employed DTPO **4** in disulfide rebridging using Trastuzumab and a very similar protocol (ESI 3.1.1) to that described above (Figure 2a). To our delight, five equivalents DTPO **4** were sufficient to achieve complete conversion of reduced Trastuzumab (5 mg ml^−1^) after overnight reaction as shown by SDS‐PAGE and intact‐protein mass spectrometry (Figure 2b,c). In contrast, no modification or rebridging could be detected without the prior addition of TCEP (Figure 2c), highlighting the cysteine selectivity of the reagent. It should be noted that although fully rebridged antibody was observed, the major product observed from the reaction was found by gel densitometry to be the so‐called half‐antibody species (half‐mAb) where the two hinge‐region cysteines of the same heavy chain have been rebridged (as depicted in Figure 2a). We also observed the formation of low levels of other partially rebridged products seen in most other rebridging methodologies (for a thorough analysis see ESI section 3.1.14). As has been demonstrated previously by us and others, partially rebridged conjugates remain functional and can reassemble in solution to form a full antibody species via non‐covalent interactions.^[^ ^17^, ^38^, ^47^ ^]^ In the major products (full and half‐mAb), the disulfide between the IgG heavy and light chain is efficiently rebridged meaning the Fab region that is responsible for antigen recognition, is covalently linked.^[^ ^17^ ^]^ Furthermore, we demonstrate the formation of the full‐antibody structure in solution by SEC analysis of the fluorescently labeled **Tras‐4** (Figure 2d), which shows similar aggregation levels for **Tras‐4** and unmodified Trastuzumab, indicating no unfavorable influence of the rebridging reagent.

We also tested if chemical modification influences the antibody's selectivity for Her2. To this end, we incubated both a Her2^+^ (SKBR‐3) and a Her2^−^ (MDA‐MB‐468) cell line with **Tras‐4** and performed live cell confocal fluorescence microscopy. The functionalized antibody exclusively stained membrane‐bound Her2 on SKBR‐3 cells, with no detectable labeling of the triple‐negative breast cancer cell line MDA‐MB‐468 (Figure S2). To further probe the reactivity and performance of DTPOs in antibody rebridging reactions, we performed a time‐course rebridging experiment using Trastuzumab (5 mg ml^−1^) and 5 eq. **4**. As anticipated, the rebridging occurred significantly faster compared to diethynyl‐phosphinates. Already after 15 min, the most prominent band (approx. 50%) was the half antibody, with almost complete conversion (>95%) reached after 2 h of reaction time based on gel densitometry (Figure S3). In contrast, previously reported diethynyl‐phosphinates (DPs) require overnight reaction to reach full rebridging (Figure S4).

Since excellent conjugate stability is a prerequisite for potential biopharmaceutical applications, we verified that the increased reactivity does not alter the linkage stability. The Trastuzumab fluorescein conjugate **Tras‐4** was incubated in glutathione supplemented rat serum. Pleasingly, over the course of 14 days neither release of fluorescent heavy‐ or light‐chain nor transfer of the fluorophore onto plasma proteins could be observed via in‐gel fluorescence (Figure 2e). This finding further underlines that DTPOs are versatile reagents for the fast and selective generation of stable bioconjugates.

**Figure 3:**
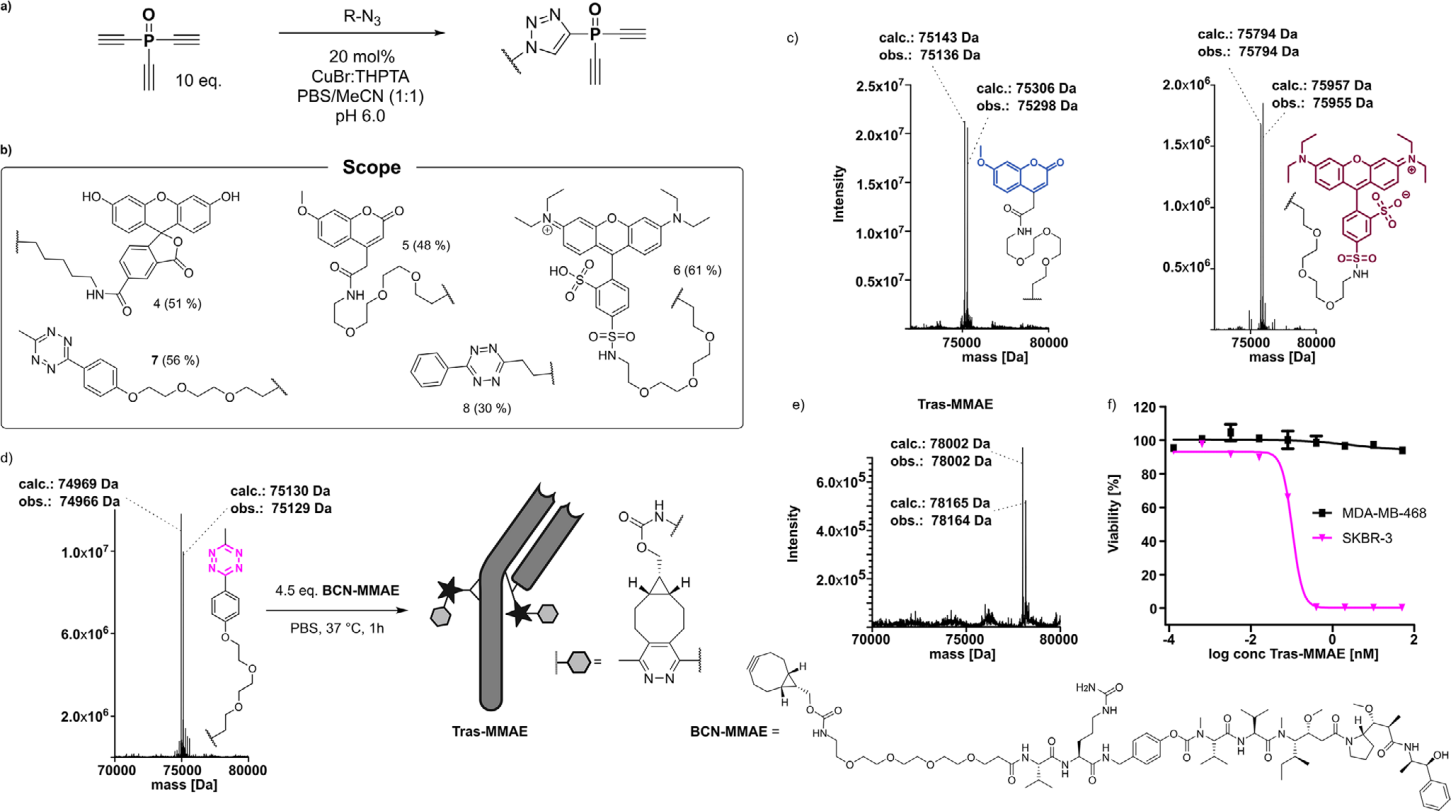
a) Optimized procedure for the synthesis of functional diethynyl‐triazolyl‐phosphine oxides (DTPOs) via CuAAC. b) Structure and isolated yield of the synthesized DTPOs **4**–**8**. c) Deconvoluted intact protein MS spectra of Trastuzumab rebridged with DTPOs **5** & **6**. d) Deconvoluted intact protein MS spectra of Trastuzumab rebridged with DTPO **7** and schematic representation of subsequent post functionalization with BCN‐MMAE via IEDDA. e) Deconvoluted intact protein MS spectra of the obtained antibody‐drug‐conjugate (**Tras‐MMAE**). f) Concentration dependent cellular cytotoxicity of the generated ADC in Her2+ (SKBR‐3, pink) and Her2‐ (MDA‐MB‐468, black) cell lines, shown are mean and standard error of the mean (SEM) values calculated from from four biological replicates which all contained triplicate datasets (*N* = 4, *n* = 3).

In subsequent experiments, we further expanded the scope of diethynyl‐phosphine oxide based rebridging reagents. For this, we reacted several easily accessible, functionalized azides with triethynyl‐phosphine oxide **2** following the CuAAC procedure (Figure 3a) followed by purification via semi‐preparative HPLC. Following this approach, various DTPOs bearing fluorophores (**4**–**6**) or bioorthogonal tetrazine handles (**7** & **8**) were obtained (Figure 3b). Importantly, the synthesis of tetrazine containing diethynyl‐P^V^ reagents is not trivial. Previous attempts to obtain tetrazine‐modified diethynyl phosphinates resulted in an unexpected intramolecular reaction between the tetrazine and one alkyne group followed by further decomposition (Figure S5 & ESI 4.1.8–9). However, we did not observe this intramolecular reaction for tetrazine DTPOs. To investigate the generality of our previous observation, we prepared a variety of functionally rebridged antibodies. For all investigated substituents, complete rebridging was achieved after only 2 h of reaction time (Figures 3c,d and S6).

Having demonstrated that DTPOs can reliably generate stable antibody conjugates without disrupting their inherent function, we aimed to utilize this technology to synthesize ADCs. Although a rebridging strategy involving the conversion of a cytotoxic drug into a DTPO for one‐step ADC formation could be envisioned, we decided to take advantage of the compatibility of tetrazine groups with our DTPO reagents to form the ADC as part of a two‐step strategy. Splitting the formation of the ADC into two steps allows for the use of **DTPO‐7** to decorate the antibody with four tetrazines and then the use of extremely fast inverse electron‐demand Diels‐Alder (IEDDA)^[^ ^48^, ^49^, ^50^ ^]^ chemistry to rapidly conjugate these to four copies of the payload of choice. This method would avoid the need for re‐optimization of the delicate rebridging step for each new payload and minimizes the direct handling of cytotoxic agents by using commercial dienophile conjugated (IEDDA‐ready) ADC payloads. Two‐step methods have been used previously to generate functional ADCs; however, they have either not measured the homogeneity of the ADCs generated or have found their second (click) step to be sluggish leading to poor homogeneity – something we hoped to avoid through the use of a fast IEDDA reaction.^[^ ^9^, ^13^, ^20^, ^21^ ^]^

We initially chose a commercially available payload linker consisting of monomethyl‐auristatin E (MMAE), a potent inhibitor of tubulin polymerization, an enzymatically cleavable peptide linker (valine‐citrulline‐*para*‐aminobenzyl) and a polyethylene glycol‐linked endo‐BCN (bicyclo[6.1.0]non‐4‐yne) moiety as the dienophile (**BCN‐MMAE**, Figure 3d). Trastuzumab intermediate **Tras‐7** was incubated for 1 h at 37 °C with 4.5 equivalents of **BCN‐MMAE**, achieving full conversion to the desired ADC as indicated by intact‐protein MS (Figure 3e). After purification via size‐exclusion‐chromatography, the cytotoxicity of the Her2 directed ADC (**Tras‐MMAE**) was investigated using MDA‐MB‐468 and SKBR‐3 cell lines. While the proliferation of SKBR‐3 cells (HER2^+^) was fully inhibited by **Tras‐MMAE** (IC_50_ = 0.1 nM), antigen‐negative cells were not affected at the concentrations tested in this experiment (Figure 3f and ESI 3.4). These results are in agreement with the previously reported cytotoxicity data for MMAE‐ETP modified Trastuzumab (DAR 4.3).^[^ ^39^ ^]^ We analyzed **Tras‐MMAE** and a comparable ADC, formed by incubating **Tras‐7** with an analogous commercial *trans*‐cyclooctene (TCO)‐linked MMAE payload, by hydrophobic interaction chromatography (HIC, see ESI 3.1.12 and 3.1.13 for details). Analysis of the resulting conjugates revealed that although the reaction with **BCN‐MMAE** to afford **Tras‐MMAE** resulted in good DAR values, the homogeneity of the conjugate on HIC was relatively low (33% DAR 4, ESI 3.1.12). The use of TCO‐linked MMAE‐payload resulted in a much improved HIC demonstrating DAR of 3.8 with 47% DAR 4 species (ESI 3.1.13) which, while modest, represents the highest recorded for the modular, two‐step procedure of a DAR 4 ADC.^[^ ^20^, ^22^ ^]^

In the course of developing a synthetic route for DTPO **4**, we observed that depending on the reaction conditions a significant amount of the doubly conjugated product (EDPO **4a**) was obtained (see ESI 4.2.1). Since we could previously demonstrate, that a triazole‐group conjugated to the phosphorus can increase the electrophilicity, we were curious if the reactivity‐trend continues with two conjugated triazole‐groups. To this end, we performed a thiol reactivity assay using the model thiol glutathione (GSH). To our delight, we observed rapid conversion of the starting product into an E/Z mixture of the corresponding GSH adduct at pH 8.5. Kinetic analysis revealed a second order rate constant of 3.3 M^−1^s^−1^ (ESI 3.5), which is comparable to our previously reported ETP‐reagents.^[^ ^39^ ^]^ In addition, we observed good stability of a **4a**‐peptide conjugate in various buffers and in the presence of a large excess glutathione over 24 h (ESI 3.6). Moreover, we could successfully use **4a** for homogeneous protein double‐modification on a single amino acid side chain as exemplified on the 66 kDa protein bovine serum albumin (BSA, ESI 3.1.15).

**Figure 4:**
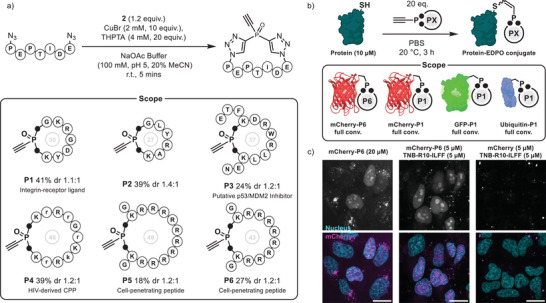
a) Optimized synthetic procedure toward cyclic, electrophilic EDPO‐peptides. Scope of synthesized peptides (**P1**‐**P6**), numbers in the middle indicate ring‐size. Azide containing amino acids are always either azido‐glycine (**P1**, **P2**) or side chain azido‐lysine (**P1‐P6**). Black dots in scope represent triazole rings, see SI for full structures. b) Schematic representation of protein‐bioconjugation reactions between cyclic peptides and cysteine‐containing proteins. For specific conditions see SI section 3.1.16–19. c) HeLa cells treated with mCherry or mCherry‐**P6** in the presence or absence of TNB‐R10‐ILFF CPP‐additive. Cells treated with mCherry‐**P6** in the presence of the CPP additive and at 20 µM concentration without CPP‐additive showing direct translocation and nuclear localization. Scale bar = 20 µm.

Based on these observations, we proposed an unprecedented approach to convert linear peptides into their cyclic, electrophilic counterparts. Unlike linear peptides, cyclic peptides offer the benefit of enhanced stability, improved binding affinity, and resistance to proteolytic degradation. We envisioned that a peptide equipped with two azide functionalities could be employed for single reagent mediated peptide cyclization and subsequent cysteine‐bioconjugation. For our initial studies and optimization of the synthetic route, we chose the small model peptide N_3_‐GKRGDYK(N_3_), a derivative of a commonly used cyclic integrin ligand (**P1**). Using the initial reaction conditions established for the synthesis of **4a**, we only observed minimal conversion to the desired peptide product. Recognizing the distinct nature of chemical transformations of peptides, we optimized the catalyst and triethynyl‐phosphine amounts for this reaction (ESI 4.4.2). We determined that at 1 mM peptide concentration, a slight excess of phosphine oxide and a tenfold excess of CuBr resulted in the best conversion and ensured short reaction times (5 min, Figure 4a). Finally, we were able to isolate the cyclized electrophilic peptide by semi‐preparative HPLC in 41% yield as a mixture of two diastereomers (due to chiral phosphorous center, see ESI 4.4.3) with a diastereomeric ratio (dr) of 1.1:1. To verify the identity of obtained product, we performed ^31^P‐NMR, which resulted in a similar shift as **4a** (‐16.38/‐16.79 ppm). Additionally, we incubated the obtained peptide with an excess (100 eq.) of reduced glutathione at pH 8.5, indicating the successful formation of the desired EDPO‐mono‐adduct. It is important to note, that we did not observe the formation of an uncyclized DTPO‐peptide as a side product (see ESI 4.4.3 for details).

Having determined suitable reaction conditions, we synthesized a small scope of electrophilic cyclic peptides of various sizes containing a multitude of functional side chains. These include a p53/MDM2 interaction inhibitor (**P3**, 24%, dr 1.2:1),^[^ ^51^ ^]^ an optimized cell‐penetrating peptide derived from the transactivator of transcription (TAT) of human immunodeficiency virus (**P4**, 39%, dr 1.2:1)^[^ ^52^ ^]^ and two polyarginine‐based cell‐penetrating peptides (R10‐peptide, **P5**, 18% dr 1.2:1; R8‐peptide, **P6**, 27%, dr 1.2:1).

Finally, we examined how these electrophilic cyclic peptides perform in protein bioconjugation. To do this, we incubated a variety of single‐cysteine containing proteins with 20 eq. of the electrophilic, cyclic RGD or R8‐peptide (**P1** or **P6**, Figure 4b) in PBS (see ESI 3.1.16–19 for exact conditions). We observed full conversion to the desired peptide‐protein conjugate via intact‐protein mass spectrometry after 3 h. Previously, our group described using cyclic polyarginine peptides to facilitate cellular delivery of proteins into living cells with immediate bioavailability.^[^ ^53^ ^]^ Following the reported protocol, we successfully demonstrated the direct transduction of **P6**‐modified NLS‐mCherry (mCherry‐**P6**) into the cytosol at an elevated concentration of 20 µM (Figure 4c). Additionally, in the presence of additional cell‐surface retaining R10s (“CPP additive technology”)^[^ ^54^ ^]^ even at lower concentrations (5 µM) efficient cellular delivery of the fluorescent protein was observed (Figure 4c, middle panel). In contrast, the unmodified protein did not lead to detectable cellular uptake. This underscores the versatility of our cyclization‐bioconjugation strategy utilizing triethynyl‐phosphine oxide **2** to furnish functional protein‐bioconjugates.

## Conclusion

In conclusion, we have successfully implemented triethynyl‐phosphine oxide **2** as a highly attractive electrophilic building block to 1) generate functional rebridging reagents for the modification of monoclonal antibodies and 2) for peptide cyclization‐bioconjugation applications. By utilizing CuAAC, we were able to straightforwardly access various functionalized DTPOs as antibody rebridging agents. Notably, DTPOs display fast cysteine reactivity, leading to complete rebridging of the antibody in just 2 h. Importantly, the resulting bioconjugates remain stable in serum and selective for their target. These properties render DTPOs particularly suitable for the preparation of ADCs in a two‐step protocol, as shown by the preparation and in vitro evaluation of a ∼DAR4 MMAE‐Trastuzumab conjugate via a tetrazine‐rebridged intermediate species. Additionally, CuAAC between **2** and two linked azide modalities furnished EDPOs. This chemistry provided a versatile synthetic route for the generation of electrophilic, cyclic peptides, whose utility was demonstrated in efficient protein bioconjugation and CPP‐mediated cellular delivery of a fluorescent protein.

Combined, these findings further expand the repertoire of P5‐labeling reagents and underscore the potential of DTPOs and EDPOs as powerful tools for the selective and stable generation of functional protein and antibody conjugates, opening up exciting prospects for chemical biology and tailored biopharmaceuticals.

## Supporting Information

The authors have cited additional references within the Supporting Information.^[^ ^55^, ^56^, ^57^, ^58^, ^59^, ^60^ ^]^

## Conflict of Interests

C.E.S. and C.P.R.H. are inventors of a patent containing parts of the chemistry described in this manuscript (WO2022223783A8).

## Supporting information



Supporting information

## Data Availability

The data that support the findings of this study are available from the corresponding author upon reasonable request.
