# Supplementary material for: Unsaturated Phosphine Oxides for Modular Antibody Rebridging and Single Reagent Peptide‐Cyclization‐Bioconjugation
Source: Angew Chem Int Ed Engl. 2025 Aug 8;64(38):e202508656. doi: 10.1002/anie.202508656 (PMC12435405; doi:10.1002/anie.202508656)
Supplement: Supplementary file 1 — Supporting information [file ANIE-64-e202508656-s001.pdf]

Unsaturated Phosphine Oxides for Modular Antibody Rebridging and Single Reagent Peptide-Cyclization-Bioconjugation

Christian E. Stieger[a,b,†] , Alastair J. McMillan[a,†], Mark A.R. de Geus[a,†], Jan Vincent V. Arafiles[a], Luise Franz[a,c], Christian P.R. Hackenberger[a,b]*

Dr. Christian E. Stieger, Dr. Alastair J. McMillan, Dr. Mark A.R. de Geus, Dr. Jan Vincent V. Arafiles, Luise Franz, Prof. Dr. Christian P.R. Hackenberger*
Chemical Biology Department
Leibniz-Forschungsinstitut für Molekulare Pharmakologie, im Forschungsverbund Berlin e.V. (FMP)
Campus Berlin-Buch, Robert-Roessle-Strasse 10, 13125 Berlin, Germany
E-mail: hackenbe@fmp-berlin.de

[b] Dr. Christian Stieger, Prof. Dr. Christian P.R. Hackenberger
Department of Chemistry
Humboldt-Universität zu Berlin
Brook-Taylor-Strasse 2, 12489 Berlin, Germany

[b] Dr. Luise Franz
Institute of Chemistry and Biochemistry
Freie Universität Berlin
Arnimallee 20, 14195 Berlin, Germany

[†] These authors contributed equally to this work.

Table of Contents

1 Supplementary Figures 4

1.1 Figure S1 4

1.2 Figure S2 5

1.3 Figure S3 6

1.4 Figure S4 7

1.5 Figure S5 8

1.6 Figure S6 9

2 General Information: 10

2.1 Chemicals and Solvents 10

2.2 Flash- and Thin-Layer Chromatography 10

2.3 Semi-Preparative HPLC 10

2.4 NMR-Spectroscopy 10

2.5 UPLC-UV/MS 11

2.6 High Resolution Mass Spectrometry 11

2.7 Intact Protein MS 11

2.8 Protein Concentration Determination 12

2.9 Deglycosylation of Modified Trastuzumab 12

2.10 Size-exclusion Chromatography 12

2.11 Hydrophobic interaction Chromatography (HIC) 13

3 Experimental Procedures 14

3.1 Protein Modification: 14

3.2 Protein expression and purification 31

3.3 Fluorescence Microscopy 36

3.4 Cell Based Anti-Proliferation Assays 37

3.5 Determination of the reaction kinetics of substituted ethynyl-ditriazolyl-phosphinates (EDPO): 38

3.6 Determination of EDPO-conjugate stability 40

4 Organic Synthesis 41

4.1 General synthesis 41

4.2 Synthesis of DTPOs 61

4.3 Solid-phase peptide synthesis (SPPS) 79

4.4 Synthesis of EDPOs and Development of the Cyclization 87

4.5 EDPO-Thiol conjugation: 105

5 Uncropped Gels 106

6 Supporting References 114

1 Supplementary Figures

1.1 Figure S1

Proposed application of phosphine oxide 2 in disulfide rebridging

Theoretical considerations for disulfide rebridging using **2**. Ideally, one alkyne remains intact after disulfide rebridging for further functionalization with an external thiol. Alternatively, **2** could be initially functionalized with a small-molecule thiol to obtain a functional rebridging reagent. Unfortunately, due to stability and reactivity limitations, both approaches were discarded.

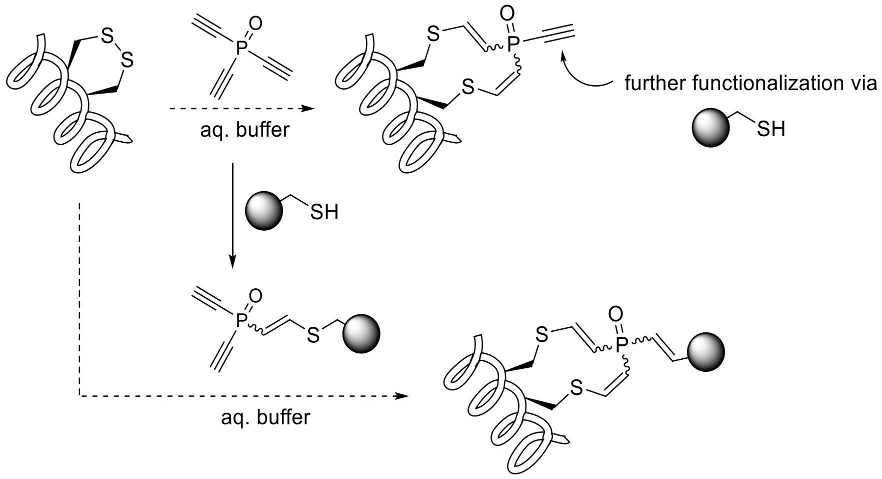

Figure S1: A depiction of the original proposal for the use of phosphine oxide **2** in disulfide rebridging.

1.2 Figure S2

Receptor dependent staining of cancer cell lines using Tras-4

Cells were seeded and incubated as described in section 3.3.1 Images were recorded on a Nikon-CSU spinning disc microscope with a CSU-X1 (Andor) and live cell incubation chamber (OKOlab). For more details, refer to section 3.3.2.

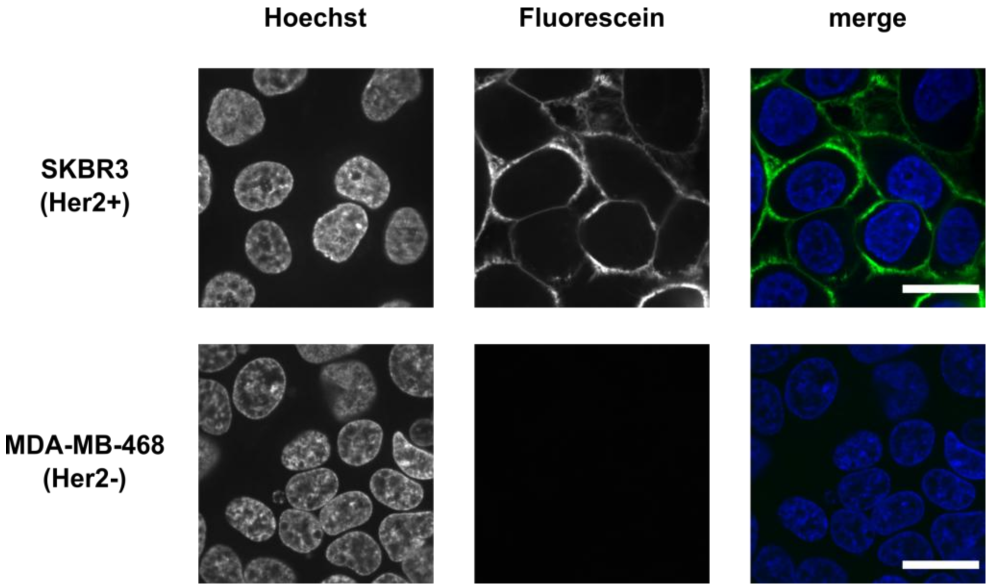

Figure S2: Her2$^{+/-}$ cells were incubated with DTPO-4 conjugated Trastuzumab (**Tras-4**). Receptor bearing cells (SKBR3) were evenly stained with no visible signal for receptor negative cells (MDA-MB-468). BFP (Hoechst 33342) ex.: 405 nm em.: 450/50 nm, GFP (antibody), ex.: 488 nm em.: 525/50 nm). Scalebar = 20 nm.

1.3 Figure S3

Time course of the rebridging of Trastuzumab with 5 eq. DTPO 4:

Trastuzumab was rebridged according to the general procedure for antibody rebridging (3.1.1) with the DTPO added in one portion. After 15, 30, 60, 90, 120, 240 and 960 minutes a 2 µl aliquot was quenched with 6x Laemmli buffer, flash frozen and stored at -20 °C until analysis by gel electrophoresis.

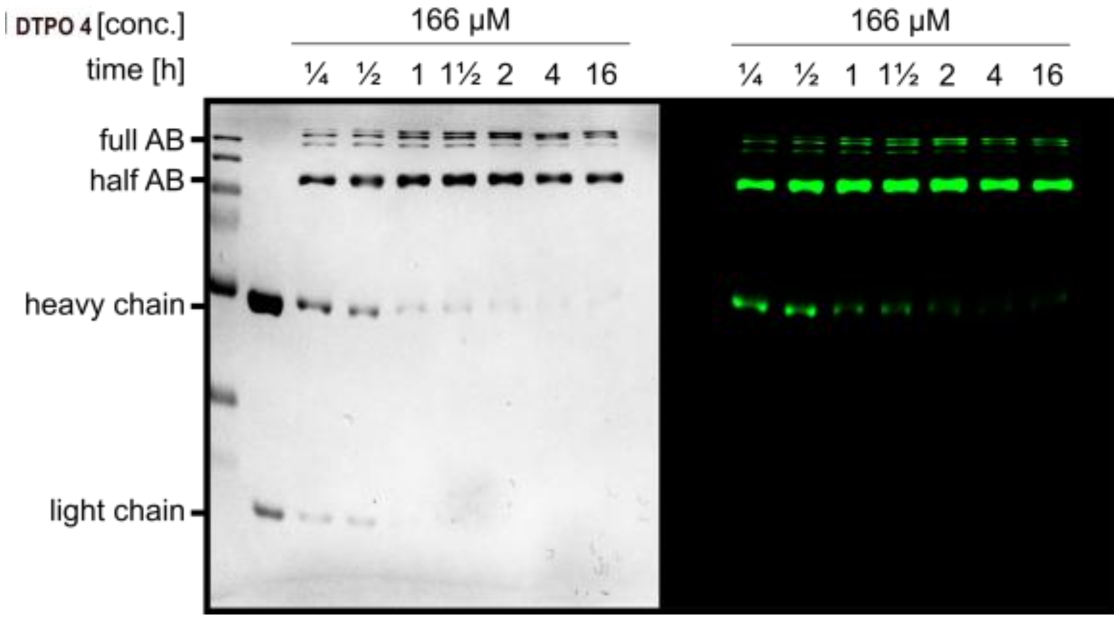

Figure S3: Left: Coomassie Staining. Right: In-Gel Fluorescence, Ex.: Blue Epi Illumination, Em.: 530/28 Filter product mixture discussed in section 3.1.14. For uncropped gel see section 5.1.4.

1.4 Figure S4

Time course of the antibody rebridging using 10 eq. butynyl diethynyl-phosphinate:

The antibody (5 mg/ml; 50 mM Tris, 1 mM EDTA, 300 mM NaCl, pH 8.3) was reduced using 8 eq. TCEP (37 °C, 30 min) followed by the reaction with 10 eq. butynyl diethynyl-phosphinate. After 1, 2, 4, 6, 8 and 24 h a 1 µl aliquot of the reaction mixture was quenched with 6x Laemmli buffer, flash frozen and stored at -20 °C until analysis by gel electrophoresis.

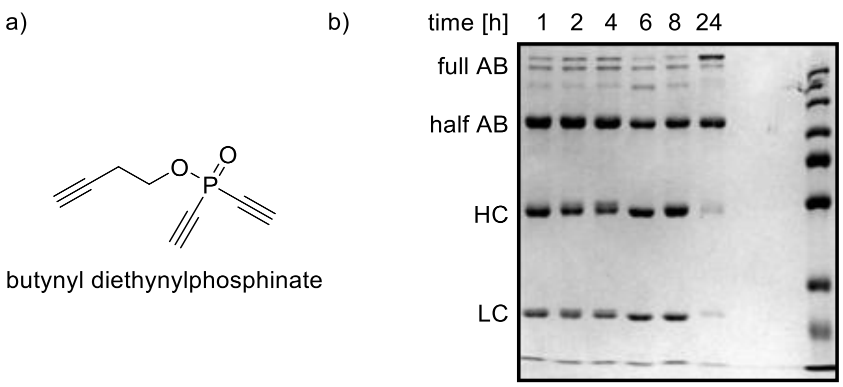

Figure S4: a) Structure of the used diethynyl phosphine oxide. b) SDS-PAGE analysis of the time-course experiment stained via Coomassie brilliant blue. For uncropped gel see section 5.1.5.

1.5 Figure S5

Unexpected side-reaction for the synthesis of tetrazine containing diethynyl-phosphinates

a)

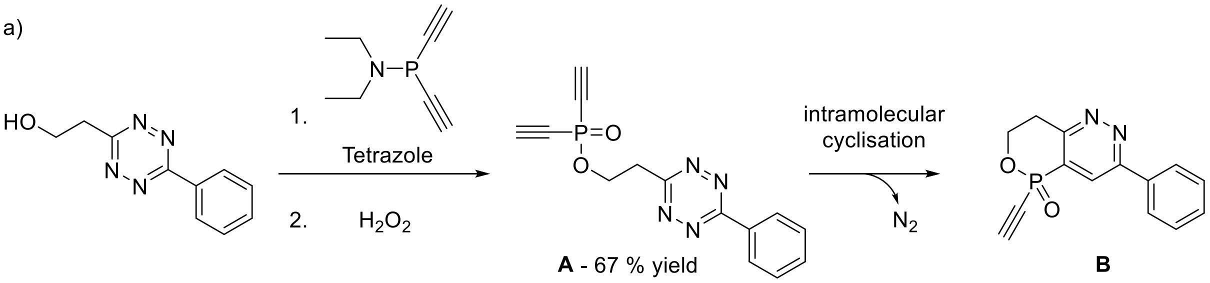

b)

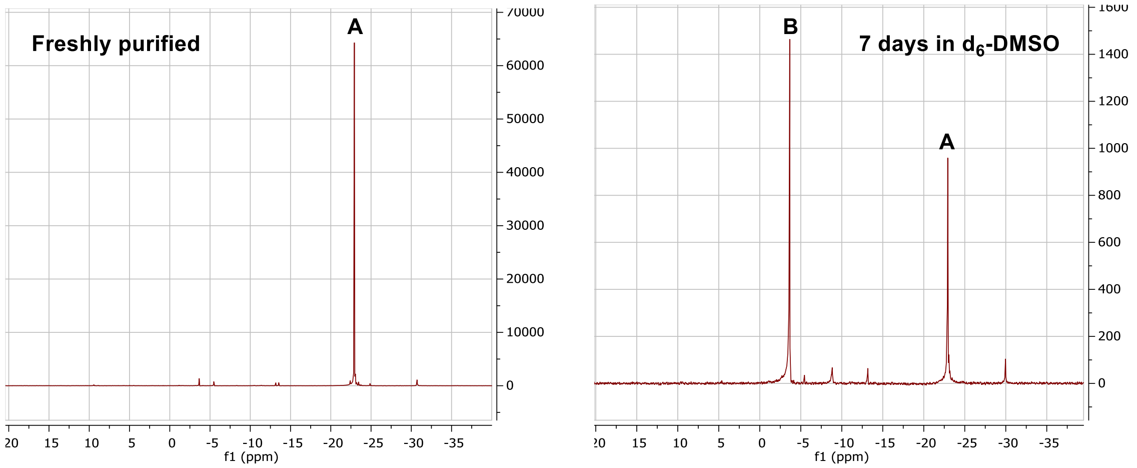

Figure S5: a) Synthetic procedure towards compound **A** and the subsequent intramolecular cyclization. b) ^{31}P-NMR spectrum of the freshly purified compound **A**. c) ^{31}P-NMR spectrum of compound **A** after 1 week.

1.6 Figure S6

SDS-PAGE analysis of Trastuzumab rebridged with 5 eq. DTPO 7 followed by conjugation to BCN-TAMRA:

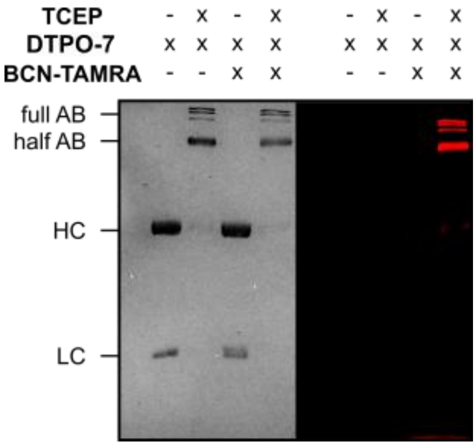

Figure S6: SDS-PAGE analysis of Trastuzumab rebridged with **DTPO 7** followed by labelling with BCN-TAMRA. (Left: Coomassie Staining. Right: In-Gel Fluorescence; product mixture discussed in section 3.1.14. For uncropped gel see section 5.1.6.

2 General Information:

2.1 Chemicals and Solvents

Chemicals and solvents were purchased from Merck (Merck group, Germany), TCI (Tokyo chemical industry CO., LTD., Japan) and Acros Organics (Thermo Fisher scientific, USA) and used without further purification. Dry solvents were purchased from Acros Organics (Thermo Fisher scientific, USA). Amino acids and resins for SPPS were purchased from Novabiochem (Merck, USA) or Iris Biotech GmbH (Germany).

2.2 Flash- and Thin-Layer Chromatography

Flash column chromatography was performed, using NORMASIL 60® silica gel 40-63 µm (VWR international, USA). Glass TLC plates, silica gel 60 W coated with fluorescent indicator F254s were purchased from Merck (Merck Group, Germany). Spots were visualized by fluorescence depletion with a 254 nm lamp or permanganate staining (10 g K_2CO_3, 1.5 g $KMnO_4$, 0.1 g NaOH in 200 ml H_2O), followed by heating.

2.3 Semi-Preparative HPLC

Semi-preparative HPLC was performed on was performed on a Shimadzu prominence HPLC system (Shimadzu Corp., Japan) with a CBM20A communication bus module, a FRC-10A fraction collector, two pumps LC-20AP, and a SPD-20A UV/VIS detector, using a VP250/21 Nucleodur C_{18} HTec Spum column (Macherey-Nagel GmbH & Co. Kg, Germany).

2.4 NMR-Spectroscopy

NMR spectra were recorded with a Bruker Ultrashield 300 MHz spectrometer and a Bruker Avance III 600 MHz spectrometer (Bruker Corp., USA) at ambient temperature. Chemical shifts δ are reported in ppm relative to residual solvent peak ($CDCl_3$: 7.26 [ppm]; DMSO-d_6: 2.50 [ppm]; acetone-d_6: 2.05 [ppm]; CD_3CN 1.94 [ppm]; 4.79 D_2O [ppm] for ^{1}H-spectra and $CDCl_3$: 77.16 [ppm]; DMSO-d_6: 39.52 [ppm]; acetone-d_6: 29.84 [ppm]; CD_3CN 1.32 [ppm]; for ^{13}C-spectra. Coupling constants J are stated in Hz. Signal multiplicities are abbreviated as follows: s: singlet; d: doublet; t: triplet; q: quartet; m: multiplet.

2.5 UPLC-UV/MS

UPLC-UV/MS traces were recorded on either a Waters H-class or an Agilent 1290 Infinity II instrument. The Waters instrument was equipped with a quaternary solvent manager, a Waters autosampler, a Waters TUV detector and a Waters Acquity QDa detector with an Acquity UPLC BEH C18 1.7 µm, 2.1 x 50 mm RP column (Waters Corp., USA). Both instruments use A: 0.1 % TFA in H_2O; B: 0.1% TFA in MeCN and all methods have a flow rate of 0.6 mL/min. If not specified, the following gradient A was used: 5% B 0.0 - 0.5 min, 5-95% B 0.5 - 3.0 min, 95% B 3.0 - 3.9 min, 5% B 3.9 - 5.0 min. Gradient B: 5% B 0.0 - 1.5 min, 5-95% B 1.5 - 13 min, 95% B 13 - 13.9 min, 95-5% B 13.9 - 13.91 min, 5% B 13.91 - 15 min.

The Agilent Instrument was equipped with 1290 High-speed pump (G7120A), 1290 Multisampler (G7167B), 1260 DAD-HS (G7117C), 1290 MCT (G7116B) equipped with a Zorbax SB-C18 2.1x50 mm 1.8-micron column and InfinityLab LC/MSD XT single quadrupole mass spectrometer. Gradient C: 5% B 0.0 - 0.5 min, 5-95% B 0.5 - 3.0 min, 95% B 3.0 - 4.5 min, 95-5% 4.5 - 5.0 min. Gradient D: 5% B 0.0 - 1.5 min, 5-95% B 1.5 - 13.5 min, 95% B 13.5 - 14.5 min, 95-5% B 14.5 - 15.0 min. Gradient E: 5% B 0.0 - 1.5 min, 5-30% B 1.5 - 6.0 min, 30-95% B 6.0 - 6.1 min, 95% B 6.1 - 7.0 min, 95-5% B 7.0 - 8.1 min, 5% B 8.1 - 9.0 min.

2.6 High Resolution Mass Spectrometry

High resolution ESI-MS spectra were recorded on a Waters H-class instrument equipped with a quaternary solvent manager, a Waters sample manager-FTN, a Waters PDA detector and a Waters column manager with an Acquity UPLC protein BEH C18 column (1.7 µm, 2.1 mm x 50 mm). Samples were eluted with a flow rate of 0.3 mL/min. The following gradient was used: **"QTof":** 0.01 % FA in H_2O; B: 0.01 % FA in MeCN. 5 % B: 0-1 min; 5 to 95 % B: 1-7 min; 95 % B: 7 to 8.5 min. Mass analysis was conducted with a Waters XEVO G2-XS QTof analyzer.

2.7 Intact Protein MS

Intact proteins were analyzed using a Waters H-class instrument equipped with a quaternary solvent manager, a Waters sample manager-FTN, a Waters PDA detector and a Waters column manager with an Acquity UPLC protein BEH C4 column (300 Å, 1.7 µm, 2.1 mm x 50 mm). Proteins were eluted with a flow rate of 0.3 mL/min with 80 °C column temperature. The following gradient was used: A: 0.01 % FA in H_2O; B: 0.01 % FA in MeCN. 5-95 % B 0-6 min. Mass analysis was conducted with a Waters XEVO G2-XS QTof analyzer. Raw data was deconvoluted with MaxEnt 1.

2.8 Protein Concentration Determination

Protein concentrations were determined by absorption spectroscopy measurements at 280 nm using the extinction coefficient and molecular weight of the protein on a NanoDrop ND-1000. In addition, or as alternative concentrations were determined by BCA assay (Thermo Fisher Scientific, USA) according to the manufacturer's protocol.

2.9 Deglycosylation of Modified Trastuzumab

5 µl of the crude antibody modification mixture (5 mg/ml) were diluted to a final protein concentration of 1 mg/ml using PBS. 1 µl PNGase-F solution (Promega, Germany, Recombinant, cloned from Elizabethkingia miricola 10 µg/µL) and 2 µL TCEP (50 mM in H_2O) were added and the solution was incubated at 37 °C for >2 h prior to analysis via intact protein MS.

2.10 Size-exclusion Chromatography

Analytical size-exclusion chromatography (A-SEC)

Analytical size-exclusion chromatography (A-SEC) was conducted using an analytical HPLC (Shimadzu LC-20A Prominence System) coupled to a fluorescence detector (Shimadzu RF-10A XL). Upon formation, Tras-**4** (5 mg/mL; 100 µL) was rebuffered into PBS twice using 0.5 mL Zeba™Spin Desalting Columns 7K MWCO (Thermo Fischer Scientific, USA) and further diluted with PBS. Trastuzumab (Herceptin®, Roche; 30 mg/mL solution) was diluted with PBS. Antibody solutions (0.3 mg/mL; 50 µL injection) were loaded onto a SEC column (TSKgel G3000SW_{XL} 7.8 mm I.D. x 30 cm, 5 µm) and analyzed during a 16 minute isocratic gradient using a phosphate buffer at pH 6.8 (100 mM Na_2HPO_4 / NaH_2PO_4, 100 mM Na_2SO_4) as the mobile phase. UV chromatograms were recorded at 280 nm and fluorescence was recorded at λ_{ex} 498 nm, λ_{em} 517. Data was plotted using GraphPad Prism 10.

Preparative size-exclusion chromatography (SEC)

Antibody reaction mixtures were purified by size-exclusion chromatography with a 25 mL Superose™ 6 Increase 10/300GL (GE Healthcare, United States) under a flow rate of 0.5 mL/min using sterile PBS (Merck, Germany) as the mobile phase. Antibody containing fractions were pooled and the final concentration was determined (see 2.8).

2.11 Hydrophobic interaction Chromatography (HIC)

Analytical hydrophobic interaction chromatography (HIC) was conducted using an analytical HPLC (Shimadzu LC-20A Prominence System) coupled to a diode array detector (SPD-M20A Prominence DAD). TCO or BCN-modified antibody solutions (see section 3.1.13, 0.2 mg/mL; 50 µL injection) were loaded onto a HIC column (TOSOH TSKgel Butyl-NPR 4.6 mm I.D. x 10 cm, 2.5 µm) and analyzed during a 20 minute 0 to 100% B gradient at 0.5 mL/min. Mobile phase A is composed of a 25 mM Na_2HPO_4 / NaH_2PO_4 + 1.5 M ($(NH_4)_2SO_4$) buffer and B of 80% 25 mM Na_2HPO_4 / NaH_2PO_4 buffer and 20% Isopropanol V/V. HIC chromatograms were recorded at 280 nm. The peaks were identified by comparison with a standard (Bentuximab vedotin (adcetris), CAS: 914088-09-8, purchased from TargetMol).

3 Experimental Procedures

3.1 Protein Modification:

3.1.1 General Procedure for antibody rebridging

To a reaction tube (Eppendorf, LoBind) containing reaction buffer (7.41 µL, 50 mM Tris, 1 mM EDTA, 300 mM NaCl, pH 8.3) was added Trastuzumab (4.23 µL from a 162 µM stock, 0.687 µmol, 1 equiv.) followed by TCEP (6.87 µL of a 1 mM stock in reaction buffer, 6.87 µmol, 10 equiv.). The reaction mixture was gently mixed using a 10 µL pipette, warmed to 37 °C and then left 1h to react. The reaction mixture was then cooled to 20 °C for 10 min before addition of half the indicated amount of DTPO (from a DMSO stock). The reaction mixture was then gently mixed with a 10 µL pipette, left 0.5 h at 20 °C and then this addition was repeated (final Trastuzumab conc. 5 mg/mL, 20 µL final reaction volume). The reaction was left at 20 °C for the indicated amount of time. Rebridging efficiency was evaluated via intact-protein mass spectrometry and/or reducing SDS-PAGE. Full-to-half-antibody ratios were obtained by in gel densitometry and analysis via Image Lab (v. 6.1.0).

Notes on performing the rebridging reaction:

- Trastuzumab stock concentrations were measured by UV-Vis (section 2.8). 5 separately diluted solutions were each measured 5 times and averaged. The following values were used for the molar extinction coefficient and molecular weight ε = 225000 M^{-1} cm^{-1} and MW = 145531.5 Da.[1]
- DTPO stock concentrations were measured using ^{31}P NMR. 40 µL of a 20 mM stock of the reagent in DMSO (by mass) was mixed with 160 µL of a 5 mM stock of triphenyl phosphine oxide (TPPO) in DMSO and the resulting mixture was analysed by ^{31}P NMR using a 3 mm NMR tube. The concentration of the reagent was then corrected based on the relative integrals of the resulting peaks (δ_{TPPO} = 24.5 ppm).
- TCEP stocks were made fresh before each reaction from TCEP•HCl.
- The DTPO reagent can also be added in one portion at slight cost of product homogeneity.

3.1.2 **Intact protein MS of Trastuzumab rebridged with 10 eq. phosphine oxide 1 overnight:**

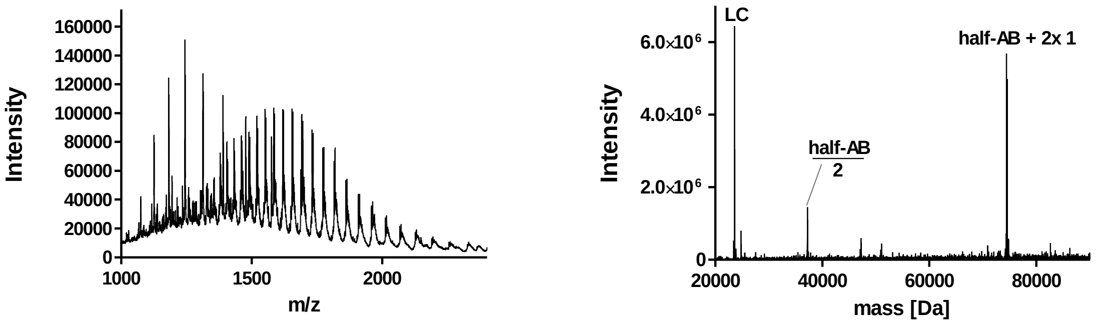

Figure S7: Raw and deconvoluted spectra of Trastuzumab rebridged with 10 eq. **1** for 16 h indicating the presence of free antibody light chain (calc.: 23438 Da, found: 23438) and rebridged half antibody (calc: 74380/74543 Da, found: 74381/74543 Da).

3.1.3 **Intact protein MS of Trastuzumab rebridged with 10 eq. phosphine oxide 2 overnight:**

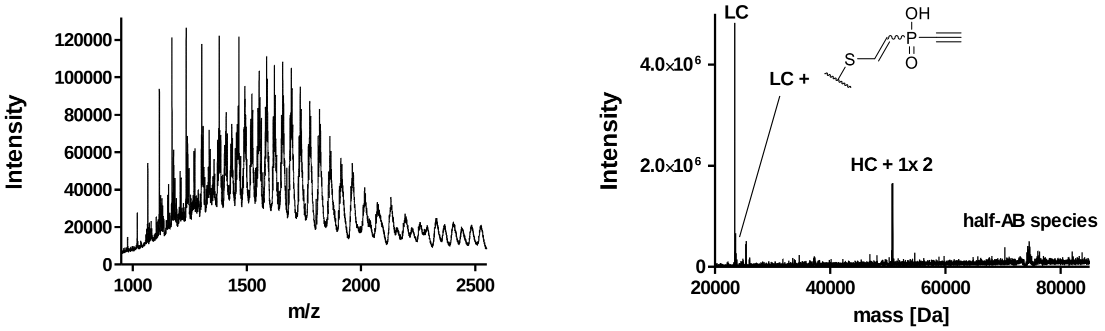

Figure S8: Raw and deconvoluted spectra of Trastuzumab rebridged with 10 eq. **2** for 16. The spectrum indicated inhomogeneous product formation and partial degradation of phosphine oxide **2**.
Light Chain (calc: 23439 Da, found: 23439 Da); Light Chain + decomposition (calc: 23553 Da, found: 23551 Da); Heavy Chain + **2** (calc: 50715/50878 Da, found: 50711/50873 Da).

3.1.4 **Intact protein MS of Trastuzumab rebridged with 10 eq. phosphine oxide 3 overnight:**

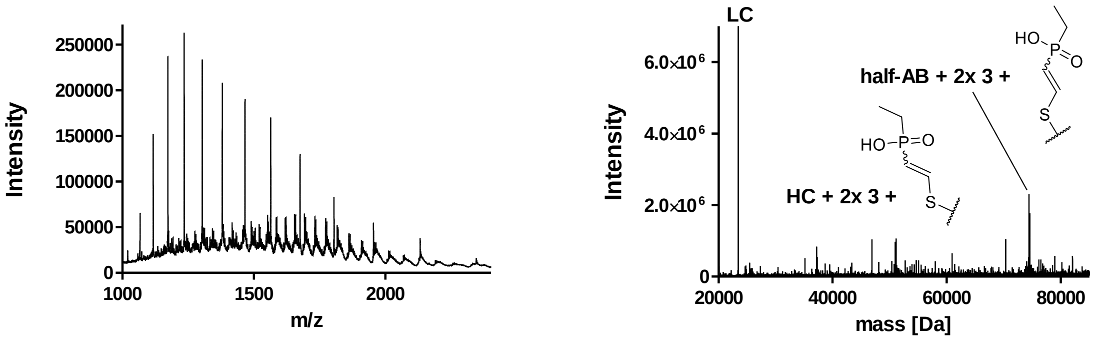

Figure S9: Raw and deconvoluted spectra of Trastuzumab rebridged with 10 eq. **3** for 16. The spectrum indicated incomplete product formation and partial degradation of phosphine oxide **3**.
Light Chain (calc: 23439 Da, found: 23439 Da); Heavy Chain + 2x **3** & decomposition (calc: 50963/51126 Da, found: 50966/51129 Da); half-AB + 2x **3** & decomposition (calc: 74402/74565 Da, found: 74404/74566 Da).

3.1.5 Intact protein MS of Trastuzumab rebridged with 5 eq. DTPO 4 overnight:

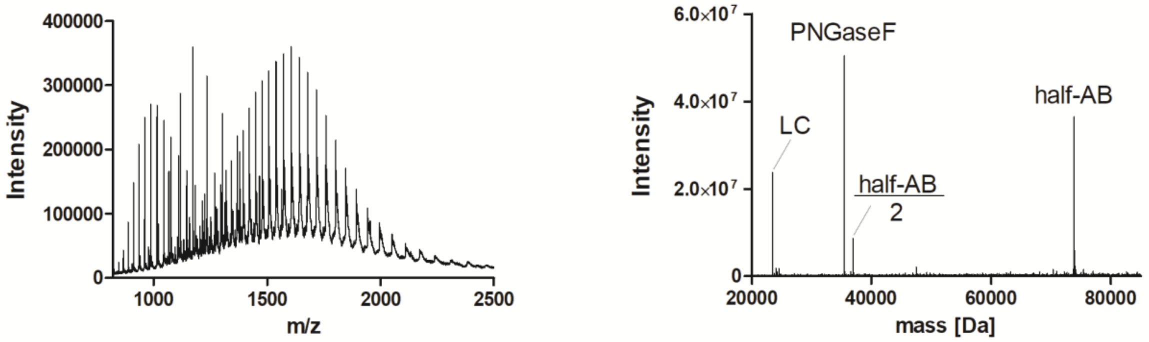

Figure S10: Raw and deconvoluted spectra of deglycosylated Trastuzumab rebridged with **DTPO 4** for 16 h followed by deglycosylation with PNGase F (see 2.9). Rebridged half antibody (calc: 73808 Da, found: 73807 Da).

3.1.6 Intact protein MS of Trastuzumab rebridged with 5 eq. DTPO 4:

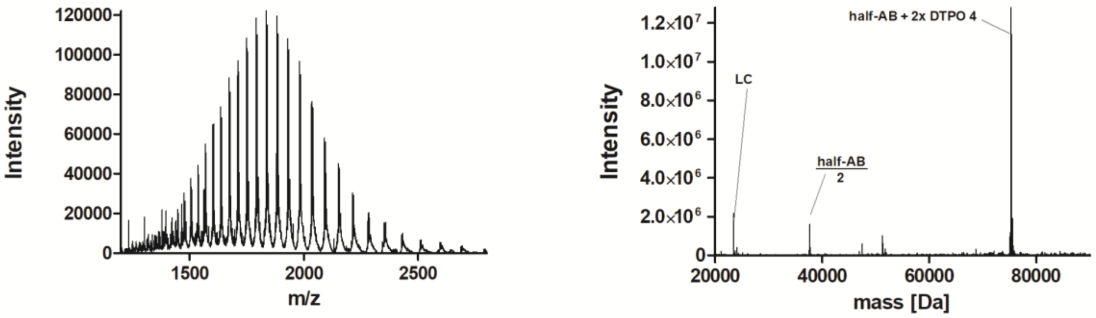

Figure S11: Raw and deconvoluted spectra of Trastuzumab rebridged with **DTPO 4** for 2 h Rebridged half antibody (calc: 74969/75130 Da, found: 73807 Da).

3.1.7 Intact protein MS of Trastuzumab rebridged with 5 eq. DTPO 5:

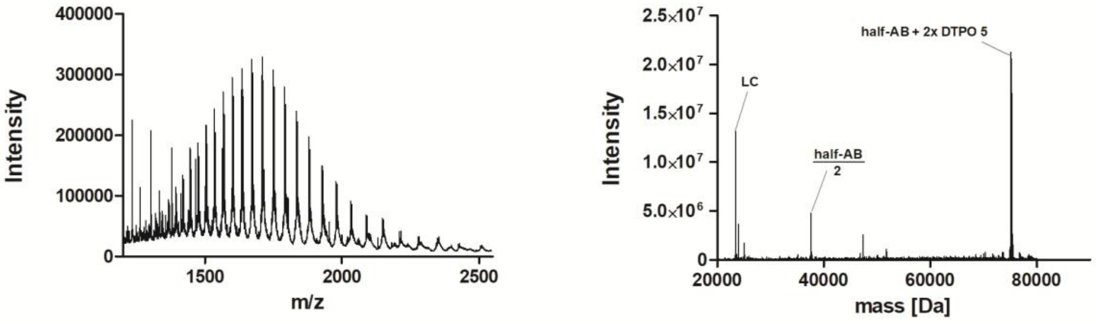

Figure S12: Raw and deconvoluted spectra of Trastuzumab rebridged with **DTPO 5**. Rebridged half antibody (calc: 75144/75307 Da, found: 75136/75298 Da).

3.1.8 Intact protein MS of Trastuzumab rebridged with 5 eq. DTPO 6:

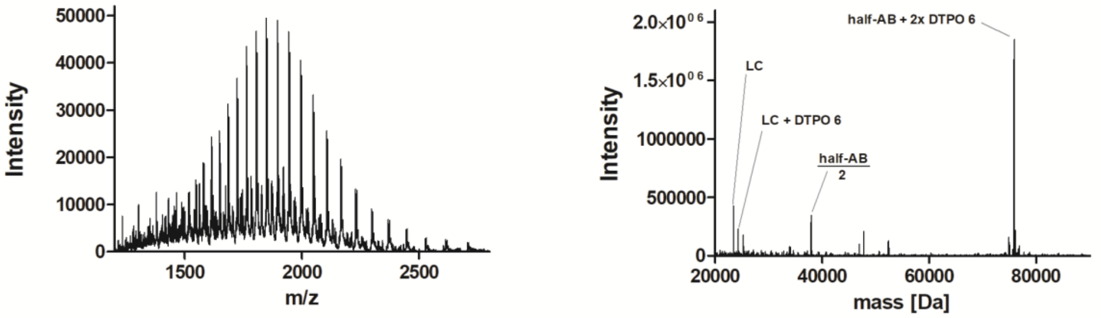

Figure S13: Raw and deconvoluted spectra of Trastuzumab rebridged with **DTPO 6**.
Rebridged half antibody (calc: 75794/75957 Da, found: 75794/75955 Da).

3.1.9 Intact protein MS of Trastuzumab rebridged with 5 eq. DTPO 7:

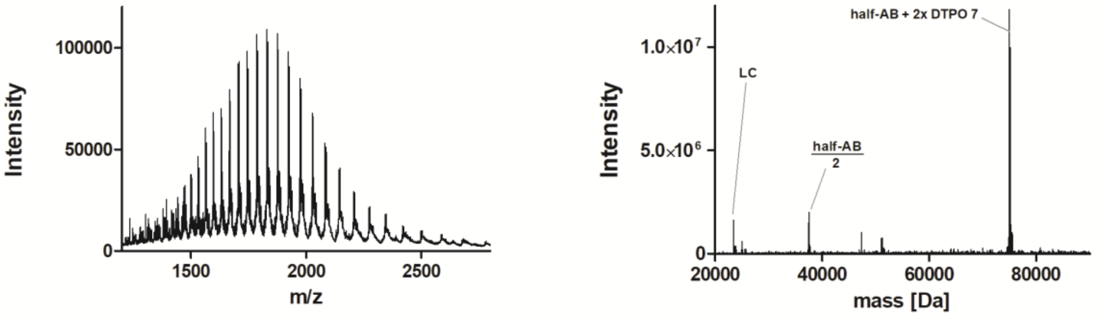

Figure S14: Raw and deconvoluted spectra of Trastuzumab rebridged with **DTPO 7**.
Rebridged half antibody (calc: 74966/75129 Da, found: 74969/75130 Da).

3.1.10 General procedures for IEDDA reactions on Trastuzumab rebridged with DTPO 7

Following the rebridging reaction described by the general procedure in 3.1.1. the reaction mixture was diluted to 1 mg/mL by addition of PBS (80 µL) and purified by spin filtration (Zeba™Spin Desalting Columns 7K MWCO (Thermo Fischer Scientific, USA)). To the reaction mixture was then added the IEDDA reagent (4.5 equiv., from a DMSO stock). The reaction mixture was then gently mixed using a 50 µL pipette then reacted at 37 °C for 1 hour. The reaction mixture was spin filtered a second time (Zeba™Spin Desalting Columns 7K MWCO (Thermo Fischer Scientific, USA)) and then modification efficiency was evaluated via intact-protein mass spectrometry.

3.1.11 **Intact protein MS of Trastuzumab rebridged with 5 eq. DTPO 7 followed by IEDDA with BCN-TAMRA:**

The IEDDA reaction was performed according to the general procedure in section 3.1.10 using endo-BCN-Ala-O2Oc-Lys(TAMRA)-Gly-NH2 (1.55 µL of a 2 mM stock, 3.1 µM, 4.5 equiv.).

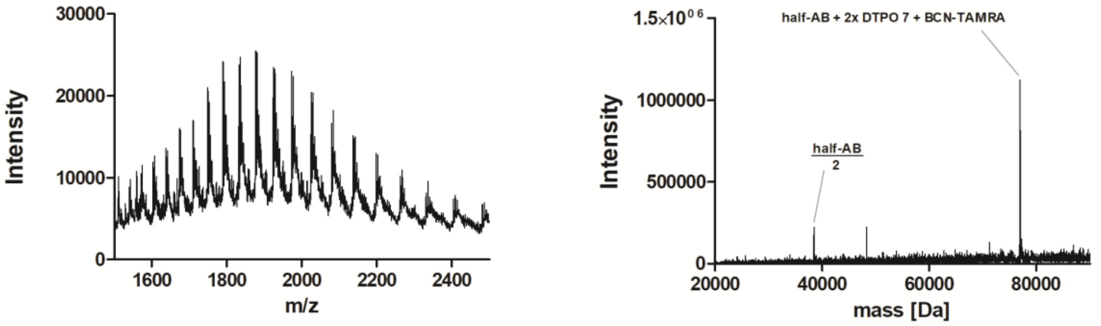

Figure S15: Raw and deconvoluted spectra of Trastuzumab rebridged with **DTPO 7** + BCN-TAMRA. Rebridged half antibody (calc: 76925/77088 Da, found: 76925/77087 Da).

3.1.12 **Intact protein MS and HIC analysis of Trastuzumab rebridged with 5 eq. DTPO 7 followed by IEDDA with BCN-MMAE:**

The IEDDA reaction was performed according to the general procedure in section 3.1.10 using endo-BCN-PEG4-VC-PAB-MMAE (1.55 µL of a 2 mM stock, 3.1 µM, 4.5 equiv.).

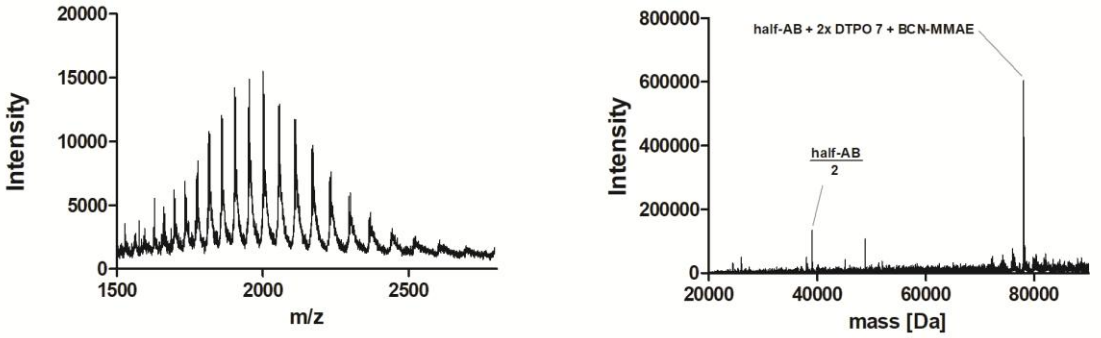

Figure S16: Raw and deconvoluted spectra of Trastuzumab rebridged with **DTPO 7** + BCN-MMAE. Rebridged half antibody (calc: 78002/78165 Da, found: 78002/78164 Da).

HIC chromatogram of BCN-rebridged Tras-MMAE

BCN coupling for HIC analysis was attempted on various Tras-7 mixtures. The best HIC attained (Figure S17) was measured from a reaction mixture of Tras-7 generated by a single addition of DTPO-7 and therefore contains increased DAR 5 product.

The rebridging reaction was carried out as described in 3.1.1 with the difference that the DTPO was added in one portion. The IEDDA reaction was performed according to a modified version of the general procedure in section 3.1.10. the reaction mixture was diluted to 1 mg/mL by addition of PBS (80 µL) and purified by spin filtration (Zeba™Spin Desalting Columns 7K MWCO (Thermo Fischer Scientific, USA)). To this reaction mixture was then added BCN-PEG4-VC-PAB-MMAE (3.4 µL of a 2 mM stock in DMSO, 6.9 µM, 10 equiv.). The reaction mixture was then gently mixed using a 50 µL pipette then reacted at 20 °C for 2 hours. The reaction mixture was then spin filtered a second time (Zeba™Spin Desalting Columns 7K MWCO (Thermo Fischer Scientific, USA)). Modification efficiency was evaluated via HIC (see section 2.11).

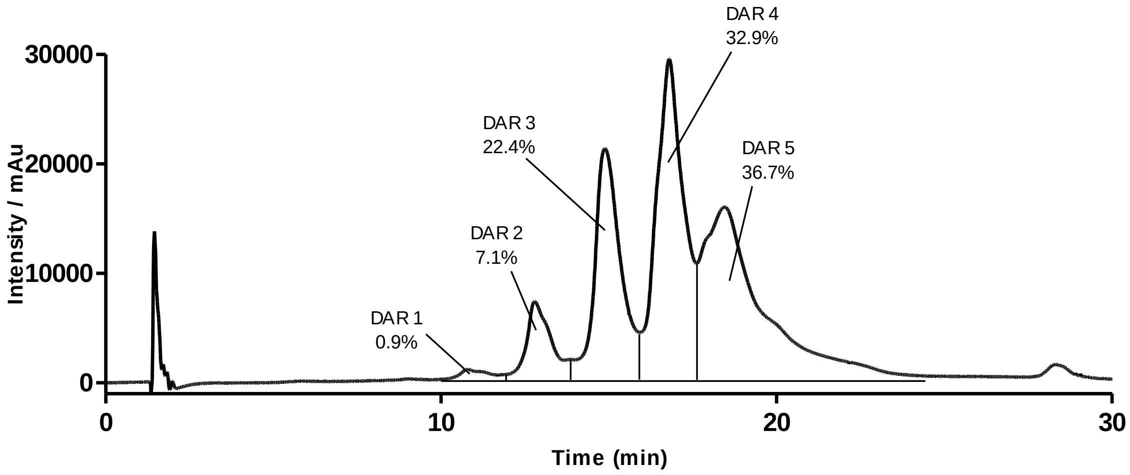

Figure S17: HIC chromatogram of Trastuzumab rebridged with **DTPO 7** followed by an IEDDA reaction with BCN-MMAE to give an overall DAR of 4.0.

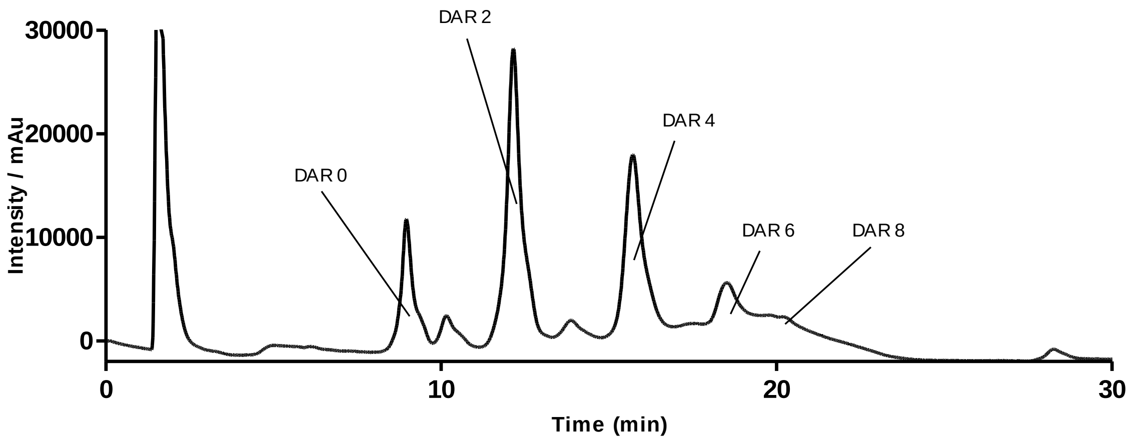

Figure S18: HIC chromatogram of Adcetris, used as a standard by which to assign the peaks in Figure S17.

3.1.13 **Intact protein MS and HIC analysis of Trastuzumab rebridged with 5 eq. DTPO 7 followed by IEDDA with TCO-MMAE:**

The IEDDA reaction was performed according to a modified version of the general procedure in section 3.1.10. Following the rebridging reaction described in 3.1.1. the reaction mixture was diluted to 1 mg/mL by addition of PBS (80 µL) and purified by spin filtration (Zeba™Spin Desalting Columns 7K MWCO (Thermo Fischer Scientific, USA)). To a 20 µL aliquot of this reaction mixture was then added TCO-PEG4-VC-PAB-MMAE (0.69 µL of a 2 mM stock in DMSO, 1.38 µM, 10 equiv.). The reaction mixture was then gently mixed using a 10 µL pipette then reacted at 20 °C for 1 hour. The reaction mixture was then diluted to 0.2 mg/mL with PBS (80 µL) spin filtered a second time (Zeba™Spin Desalting Columns 7K MWCO (Thermo Fischer Scientific, USA)). Modification efficiency was evaluated via HIC (see section 2.11).

Optimization notes:

Tras-7 stocks were kept at 4 °C after rebridging for several days before IEDDA steps without noticeable adverse effects on homogeneity.

Significant effects on homogeneity were seen when even slightly impure dienophile stocks were used.

Using the above procedure, the use of 5 dienophile equivalents resulted in a mildly reduced homogeneity, increasing the equivalents past 10 resulted in no noticeable effect.

Intact protein MS of Trastuzumab rebridged with 5 eq. DTPO 7 followed by IEDDA with TCO-MMAE

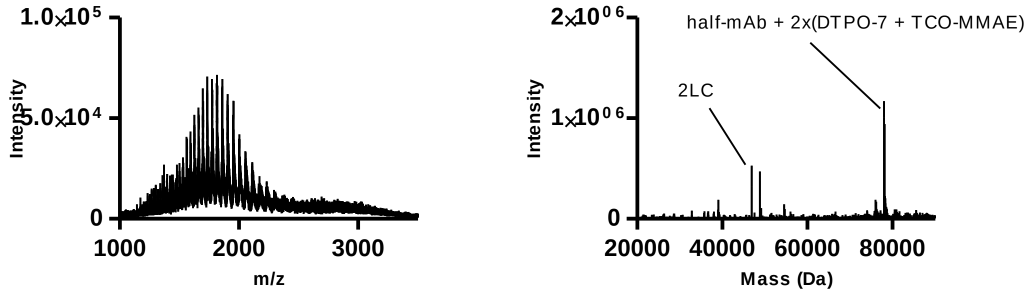

Figure S19: Raw and deconvoluted spectra of Trastuzumab rebridged with **DTPO 7** + TCO-MMAE. Rebridged half antibody (calc: 77957/78119 Da, found: 77958/78120 Da).

HIC chromatogram of TCO-rebridged Tras-MMAE

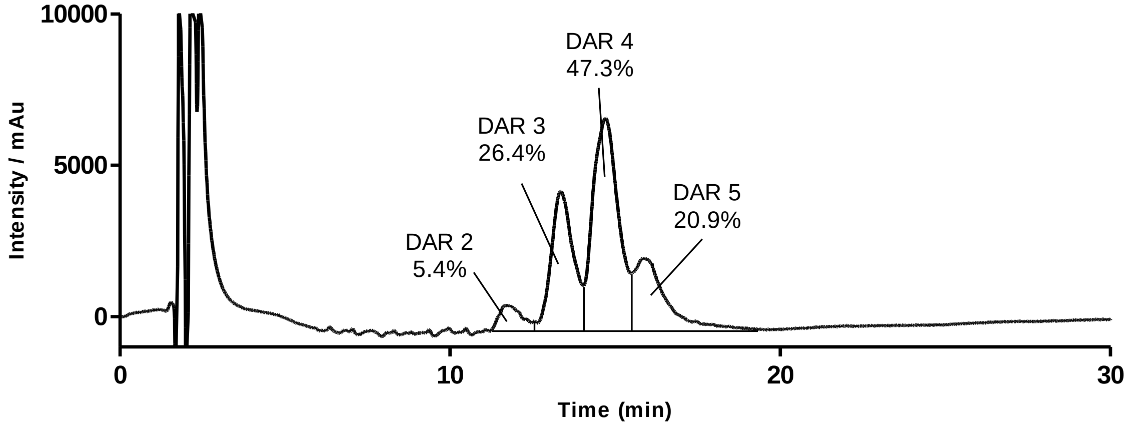

Figure S20: HIC chromatogram of Trastuzumab rebridged with **DTPO 7** followed by an IEDDA reaction with TCO-MMAE demonstrating the DAR 4 species as the major product and an overall DAR of 3.8.

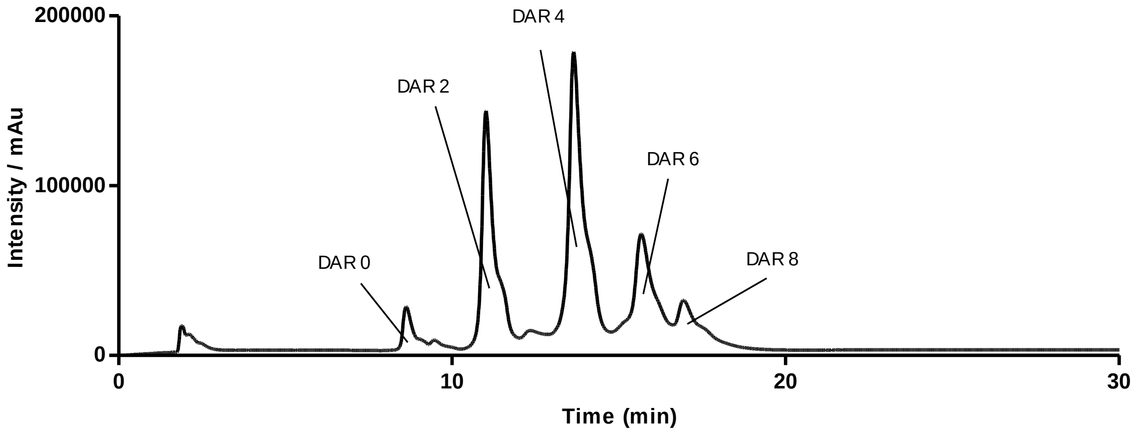

Figure S21: HIC chromatogram of Adcetris, used as a standard by which to assign the peaks in Figure S20.

3.1.14 **Rebridging product analysis and comparison**

To assess the rebridging efficiency of our reagent and to evaluate the range of products formed we have analyzed our reaction mixture using intact protein MS and SDS-PAGE gel densitometry. We have also performed the same analysis to a reaction mixture generated using of 4-(3-tosyl-2-(tosylmethyl)propanoyl)benzoic acid (TTPBA) according to the following procedure based on that described in the literature.[2]

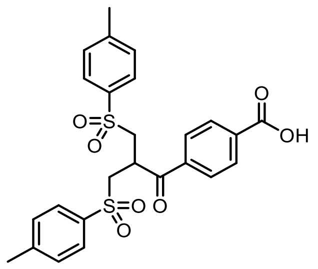

4-(3-tosyl-2-(tosylmethyl)propanoyl)benzoic acid
TTPBA

To a reaction tube (Eppendorf LoBind, 500 µL) containing reaction buffer (22.4 µL, 20 mM Na_2PO_4 buffer, 20 mM EDTA, 150 mM NaCl, pH 7.5) was added Trastuzumab (7.35 µL from a 187 µM stock, 1.37 µmol, 1 equiv.) followed by TCEP (8.24 µL of a 1 mM stock in reaction buffer, 8.25 µmol, 6 equiv.). The reaction mixture was gently mixed using a 20 µL pipette and then left at 40 °C for 1 h to react. The reaction mixture was then cooled to 20 °C for 10 min before addition of TTPBA (1.0 µL from an 8.25 mM stock in DMSO, 8.25 µmol, 6 equiv.). The reaction mixture was then gently mixed with a 20 µL pipette and left at 20 °C for 22 h. Rebridging efficiency was evaluated via intact-protein mass spectrometry and reducing SDS-PAGE.

Intact protein MS analysis

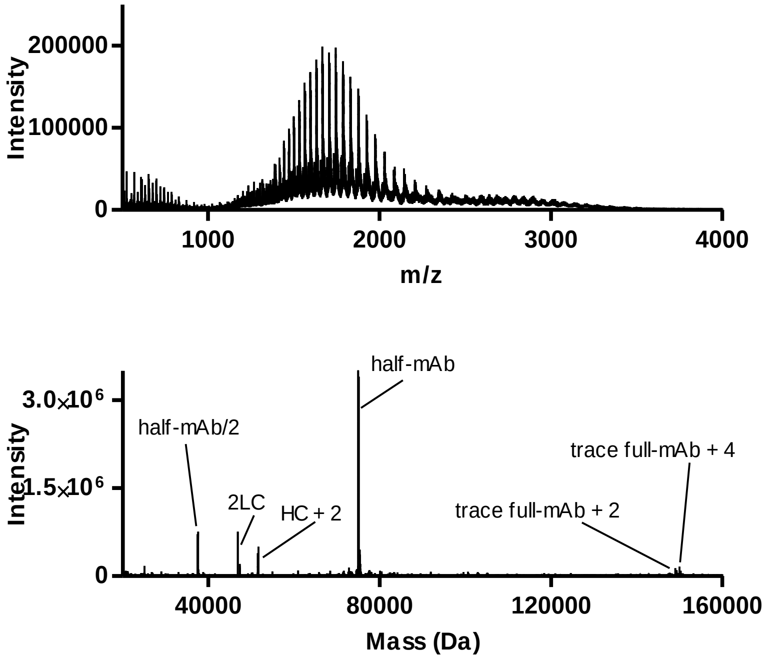

Figure S22: Raw and deconvoluted spectra of Trastuzumab rebridged with **DTPO 7**. half-mAb/2 = half mass of the half-mAb with 2 modifications (calc: 37485/37566 Da, found 37485/37566 Da), 2LC = 2x mass of the light chain (calc: 46878 Da, found 46878 Da), HC + 2 = heavy chain with 2 modifications (calc: 51530/51692 Da, found 51531/51692 Da), half-mAb = half-mAb with 2 modifications (calc: 74969/75131 Da, found 74969/75132 Da), full-mAb + 2 = full-mAb with 2 modifications (calc: 149004/149328 Da, found: 149003/149324 Da), full-mAb + 4 = full-mAb with 4 modifications (calc: 149938/150262 Da, found: 149938/150265 Da).

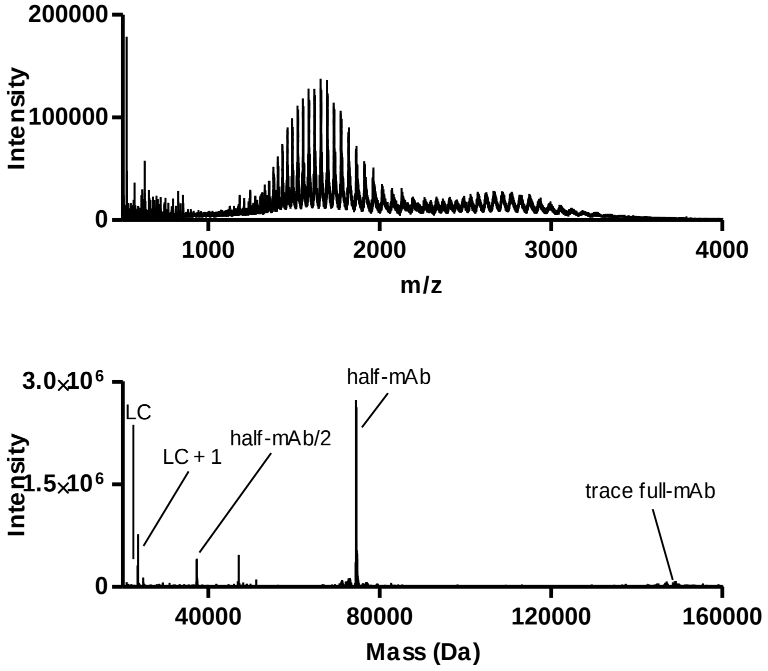

Figure S23: Raw and deconvoluted spectra of Trastuzumab rebridged with TTPBA. LC = light chain (calc: 23439 Da, found: 23440 Da), LC + 1 = light chain with 1 modification (calc: 23627 Da, found 23628 Da), half-mAb/2 = half mass of the half-mAb with 2 modifications (calc: 37206/37287 Da, found 37205/37286 Da), half-mAb = half-mAb with 2 modifications (calc: 74411/74573 Da, found 74410/74573 Da), full-mAb = full-mAb with 4 modifications (calc: 148822/149146 Da, found: 148821/149141 Da).

Intact MS discussion and comparison

Both reaction mixtures demonstrate similar deconvoluted mass spectra. In both cases the rebridged half-mAb is seen to be the major product, only a trace amount of rebridged full-mAb can be seen although the apparent low level of full-mAb can be explained by poor quantification of these large species by MS methods.[3] In both cases there are some signs of undermodification (remaining LC and full-mAb + 2 from the DTPO 7 reaction or just remaining LC in the TTPBA reaction) and overmodification (HC + 2 in the DTPO 7 reaction and LC + 1 in the TTPBA reaction). The raw spectra for DTPO 7 has a decreased ionization envelope at m/z ~2900 compared to that of TTPBA suggesting decreased presence of full-mAb species.

SDS-PAGE gel densitometry analysis

For the gel in Figure S24 a) Reaction crude (1 µL), was mixed with water (MilliQ, 6.5 µL) and reducing Laemlli buffer (4X, 2.5 µL). For the gel in figure S24 b) reaction crude (1 µL) was diluted with water (MilliQ, 14 µL) and reducing Laemlli buffer (4X, 5 µL) and for the trastuzumab lanes was mixed trastuzumab (1 µL of a 5 mg/mL stock), water (MilliQ, 29 µL) and Laemlli buffer (4X, 10 µL). Mixtures were denatured by heating to 95 °C for 8 minutes. 8 µL of mixture was added per lane and 4 µL of ladder solution (ThermoScientific, PageRuler™ Plus). 4-20% density polyacrylamide precast gels (Mini-PROTEAN TGX, 10 well) were used. Staining was performed with FastGene Q-Stain and gel imaging with a Bio-Rad ChemDoc MP imaging system. Gel analysis was performed with ImageLab 6.1.

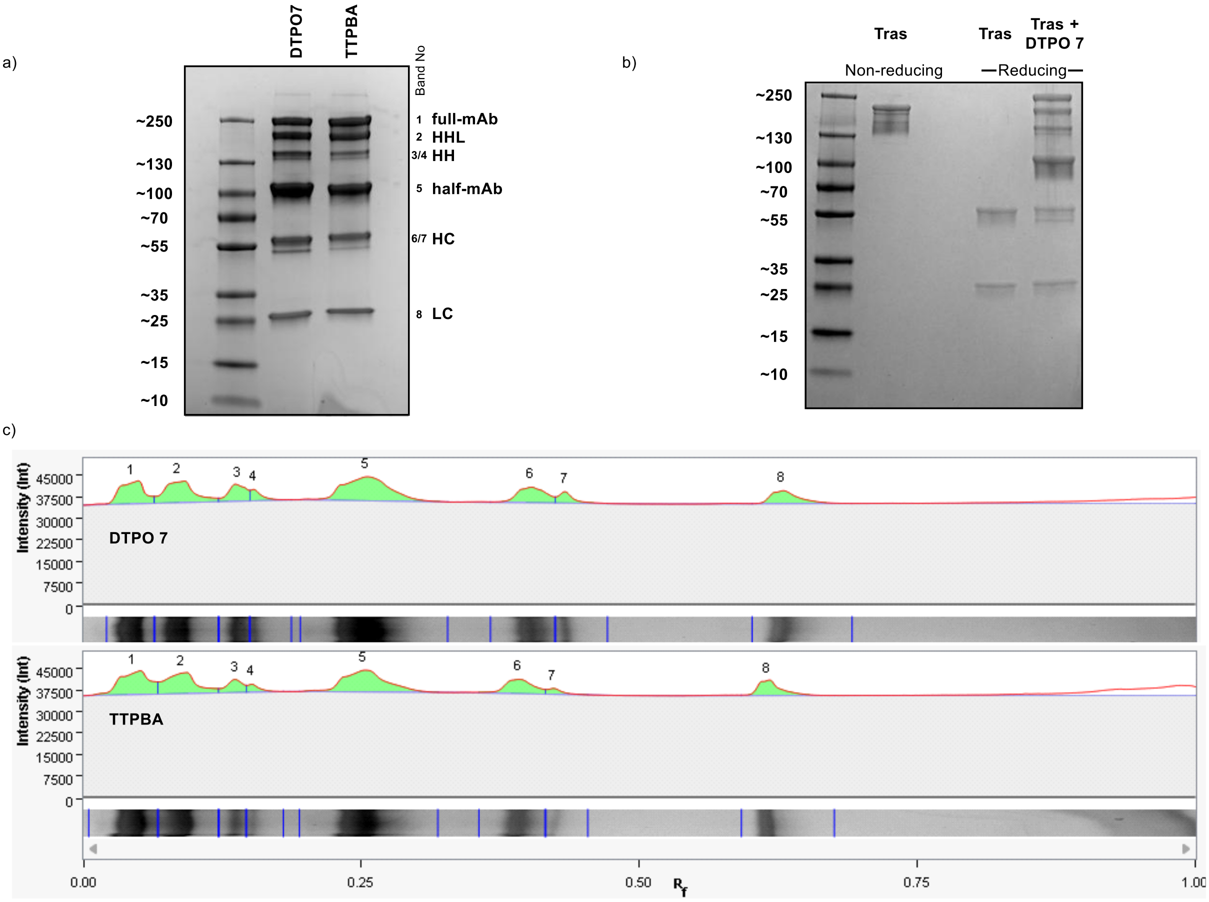

d)

Reagent	Band 1	Band 2	Band 3	Band 4	Band 5	Band 6	Band 7	Band 8	Ratio of full:half-mAb bands
DTPO 7	15.2%	17.3%	8.2%	3.3%	30.1%	12.0%	4.3%	9.7%	1:1.98
TTPBA	20.9%	18.6%	6.0%	2.9%	27.0%	12.0%	2.5%	10.2%	1:1.29

Figure S24: a) SDS-PAGE gel comparing Trastuzumab rebridged with DTPO-7 and TTPBA, Coomassie stain, bands numbered and labeled with presumed identities. HHL = two heavy chains and 1 light chain, HH = two heavy chains, HC = heavy chain, LC = light chain. b) SDS-PAGE gel comparing Trastuzumab under reducing and non-reducing conditions with the products of the reaction of trastuzumab with DTPO 7, Coomassie stain. c) Lane profiles of the DTPO-7 and TTPBA lanes from gel a made in Image Lab 6.1. d) area of each band from the lane profile as percentage of the total integrated area and calculated ratio of rebridged full-mAb band (band 1) to rebridged half-mAb band (band 5).

SDS-PAGE gel densitometry discussion and comparison

Both lanes contain the same bands and therefore very likely analogous products. There remains some presence of HC and LC bands in both reaction mixtures in agreement with the intact MS analysis which showed some unreacted LC and overreacted LC or HC (indistinguishable here). Similarly, the gel agrees with the MS analysis that the half-mAb is the major product in both cases. Where the gel disagrees is in the increased quantity of full-mAb and the presence of rebridged HH and HHL species which are not seen by intact MS. The ratio of the products differs between the two reagents with DTPO 7 being shown to give increased fraction of half-mAb than that provided by TTPBA which correlates with the size of the ionization envelopes in the raw MS data. Rebridged antibodies do not line up with their expected protein ladder mass but do match with unreacted trastuzumab. The HH and HHL species along with the identity of the full and half-mAb bands were assigned based on literature reports.[4]

3.1.15 Recombinant Albumin modification with EDPO 4a

10 equivalents of EDPO 4a

To a solution of recombinant Albumin (50 µL of 10 µM solution in PBS, 1 equiv.) was added EDPO **4a** (0.5 µL of 10 mM stock in DMSO, overall concentration 100 µM, 10 equiv.). The reaction was left at room temperature for 7 hours after which the reaction had reached almost full conversion. Modification efficiency was evaluated via intact-protein mass spectrometry.

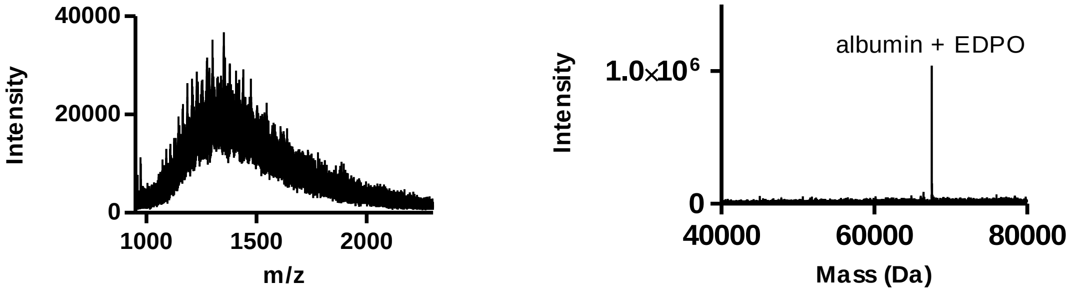

Figure S25: Raw and deconvoluted spectra of recombinant albumin modified with EDPO **4a** indicating the presence of product (calc.: 67536 Da, found: 67535 Da). Only trace starting material peak (66442 Da) remaining.

20 equivalents of EDPO 4a

To a solution of recombinant Albumin (50 µL of 10 µM solution in PBS, 1 equiv.) was added EDPO **4a** (1 µL of 10 mM stock in DMSO, overall concentration 200 µM, 20 equiv.). The reaction was left at room temperature for 3 hours after which the reaction had reached full conversion. Modification efficiency was evaluated via intact-protein mass spectrometry.

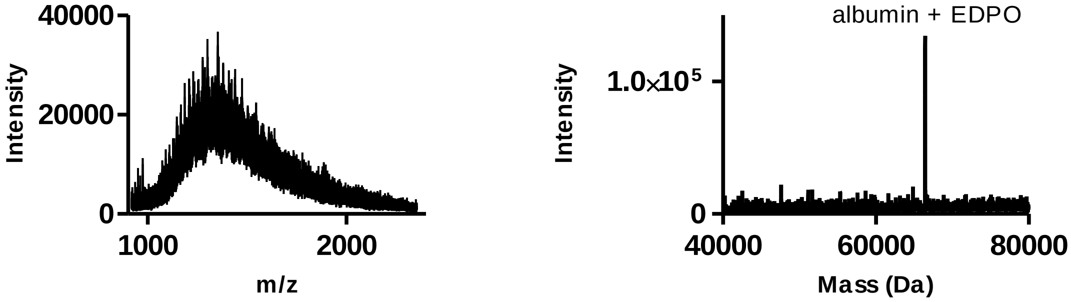

Figure S26: Raw and deconvoluted spectra of recombinant albumin modified with EDPO **4a** indicating the presence of product (calc.: 67536 Da, found: 67535 Da). No starting material peak (66442 Da) remaining.

3.1.16 **mCherry modification with EDPO P6**

To a solution of NLS-mCherry-cysteine (20 µL of 20 µM stock in PBS, 0.4 µmol, 1 equiv.) was added TCEP•HCl (6 µL in 100 µM stock in PBS, 0.6 µmol, 1.5 equiv.) followed by additional PBS (10 µL). The resulting solution was shaken at 37 °C for 30 minutes before addition of EDPO **P6** (mix of diastereoisomers, 4 µL of 2 mM stock in DMSO, 8 µmol, 20 equiv.) after which the reaction was shaken at 20 °C for 3 hours after which full conversion was observed. Modification efficiency was evaluated via intact-protein mass spectrometry.

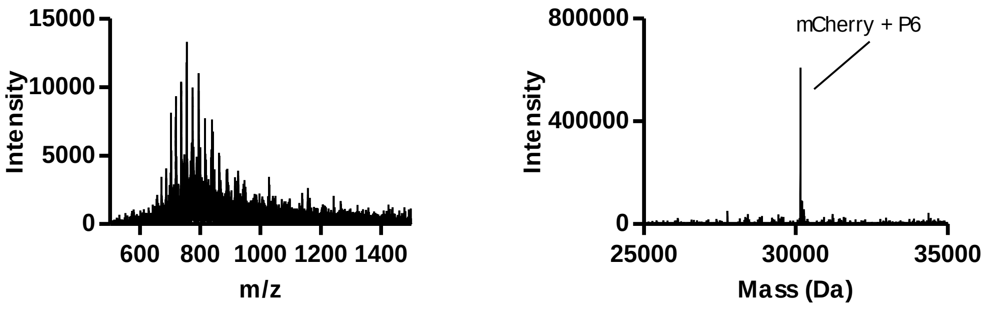

Figure S27: Raw and deconvoluted spectra of NLS-mCherry-cysteine modified with EDPO **P6** indicating the presence of product (calc.: 30149 Da, found: 30150 Da). Starting material peak (28338 Da) is no longer present.

3.1.17 **mCherry modification with EDPO P1'**

To a solution of NLS-mCherry-cysteine (20 µL of 20 µM stock in PBS, 0.4 µmol, 1 equiv.) was added TCEP•HCl (6 µL in 100 µM stock in PBS, 0.6 µmol, 1.5 equiv.) followed by additional PBS (10 µL). The resulting solution was shaken at 37 °C for 30 minutes before addition of EDPO **P1'** (4 µL of 2 mM stock in DMSO, 8 µmol, 20 equiv.) after which the reaction was shaken at 20 °C for 3 hours after which full conversion was observed. Modification efficiency was evaluated via intact-protein mass spectrometry.

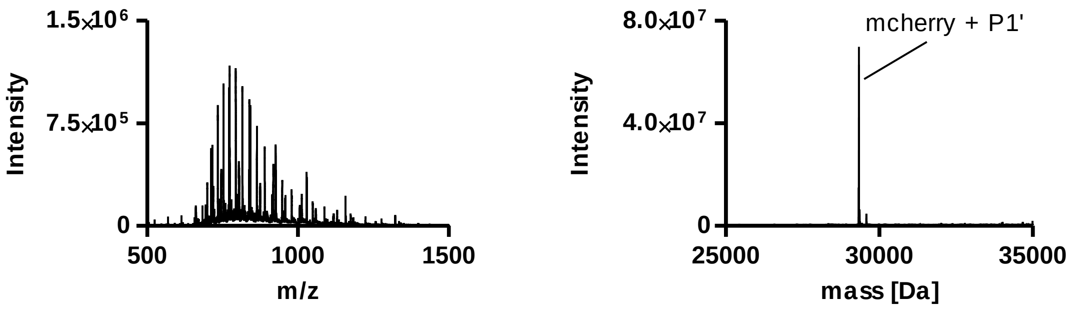

Figure S28: Raw and deconvoluted spectra of NLS-mCherry-cysteine modified with EDPO **P1'** indicating the presence of product (calc.:29333 Da, found: 29334 Da). Starting material peak (28338 Da) is no longer present.

3.1.18 GFP modification with EDPO P1”

To a solution of sf-GFP C69S AAACA (20 µL of 20 µM stock in PBS (pH 8.5), 0.4 µmol, 1 equiv.) was added TCEP•HCl (6 µL in 100 µM stock in pH 8.5 PBS, 0.6 µmol, 1.5 equiv.) followed by additional PBS (10 µL, pH 8.5). The resulting solution was shaken at 37 °C for 30 minutes before addition of EDPO **P1”** (4 µL of 2 mM stock in DMSO, 8 µmol, 20 equiv.) after which the reaction was shaken at 20 °C for 3 hours after which full conversion was observed. Modification efficiency was evaluated via intact-protein mass spectrometry.

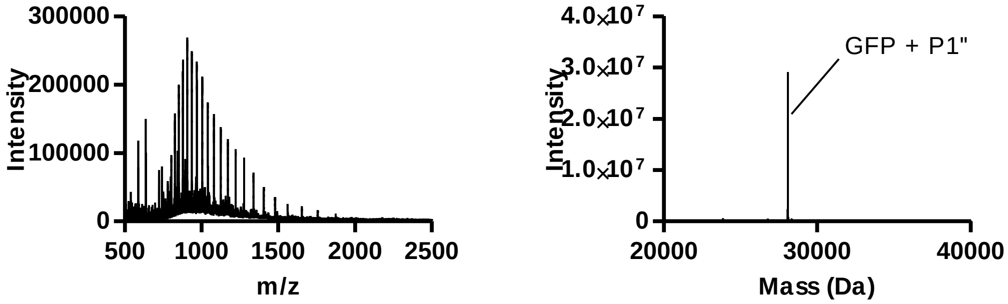

Figure S29: Raw and deconvoluted spectra of sf-GFP C69S AAACA modified with EDPO **P1”** indicating the presence of product (calc.:28079 Da, found: 28080 Da). Starting material peak (27084 Da) is no longer present.

3.1.19 **Ubiquitin modification with EDPO P1’**

To a solution of G76C-Ubiquitin (20 µL of 20 µM stock in PBS (pH 8.5), 0.4 µmol, 1 equiv.) was added TCEP•HCl (14 µL in 100 µM stock in PBS (pH 8.5), 1.4 µmol, 3.5 equiv.). The resulting solution was shaken at 37 °C for 30 minutes before addition of 4-azidobenzoic acid (2 µL of 20 mM stock in DMSO, 40 µmol, 100 equiv.). The reaction was shaken for a further 10 minutes at 37 °C before addition of EDPO **P1’** (4 µL of 2 mM stock in DMSO, 8 µmol, 20 equiv.) after which the reaction was shaken at 20 °C for 3 hours resulting in full conversion. Modification efficiency was evaluated via intact-protein mass spectrometry.

Notes: At 10 mM, TCEP reduction of ubiquitin disulfide dimers was slow so excess TCEP was used. To prevent formation of product containing TCEP adducts, 4-Azidobenzoic acid was used here to quench excess TCEP before the conjugation step.

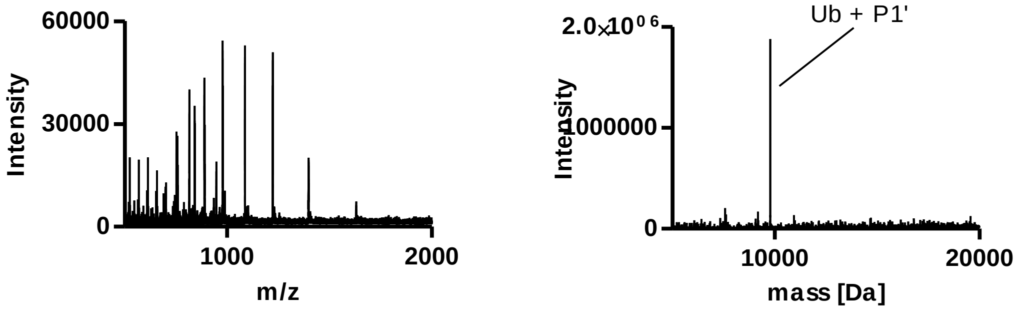

Figure S30: Raw and deconvoluted spectra of G76C-Ubiquitin modified with EDPO **P1’** indicating the presence of product (calc.:9776 Da, found: 9777 Da). Starting material peaks (17562/ 8781 Da, dimer/monomer) are no longer present.

3.2 Protein expression and purification

3.2.1 NLS-mCherry-cysteine

Protein was expressed and purified as reported in previous work from the group. The procedures are reproduced here below for ease of access.[5]

Protein sequence (Sequence after thrombin cleavage underlined, chromophore in red, cysteine in blue):

MGSSHHHHHHSSGLVPRGSHMPAAKRVKLDMVSKGEEDNMAIIKEFMRFKVHME
GSVNGHEFEIEGEGEGRPYEGTQTAKLKVTKGGPLPFAWDILSPQFMYGSKAYVK
HPADIPDYLKLSFPEGFKWERVMNFEDGGVVTVTQDSSLQDGEFIYKVKLRGTNFP
SDGPVMQKKTMGWEASSERMYPEDGALKGEIKQRLKLKDGGHYDAEVKTTYKAK
KPVQLPGAYNVNIKLDITSHNEDYTIVEQYERAEGRHSTGGMDELYKACA*

For the expression, BL21 DE3 cells were transformed with the plasmid. 2 colonies from an agar plate were picked and grown overnight at 37°C in LB medium (1 L) with 40 µg/mL Kanamycin until an OD_{600} of 0.6-0.8 was reached. Induction performed using IPTG (1 mM). Cells were collected by centrifugation at 4000 g for 15 minutes. The cells were washed once in PBS, then resuspended in PBS (30 mL) containing DNase I and Lysozyme before being lysed *via* sonication (6 min, 25% Amplitude), followed by debris centrifugation at 50,000 g for 15 min. For purification the supernatant was reduced using DTT (1 mM, r.t., 30min) before purification over Ni beads (PureCube 100 Ni-INDIGO agarose). To beads equilibrated with PBS containing imidazole (20 mM) was loaded the reduced lysate containing imidazole (20 mM). The beads were washed with 5 CV of PBS containing imidazole (20 mM). Proteins were eluted with PBS containing imidazole (500 mM).Thrombin cleavage was then performed (1:1000 v/v, overnight, r.t.), in a dialyisis bag (3.5 kDa MWCO, 2.5 L PBS). Collected protein was reduced with DTT (1 mM) and concentrated (Vivaspin 20, 10k MWCO) to 4.5 mL and then purified by SEC (Superdex75 HiLoad 16/60 overnight in PBS 7 °C). The pure protein was isolated in a yield of 140 mg. Protein aliquots were stored at -80 °C.

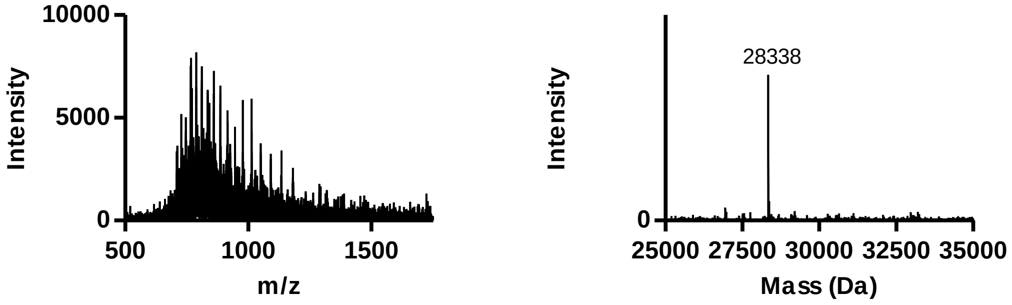

Figure S31: Raw and deconvoluted HR-MS (ESI) spectra of NLS-mCherry-cysteine. Calcd: 28338 Da, found: 28338 Da.

3.2.2 **sf-GFP C69S AAACA**

SpyCatcher003-sfGFP was a gift from Mark Howarth (Addgene plasmid # 133449 ; http://n2t.net/addgene:133449 ; RRID:Addgene_133449). The sfGFP coding region was amplified using the following primer pairs: Forward: 5'-*TGGCTAGCGAAAACCTGTATTTTCAGATGCGTAAAGGCGAAGAGCTGTTCAC*-3'; Reverse: 5'-*TAGATCCTCATTTGTACAGTTCATCCATACCATGC*-3'. and cloned into pET28a by standard restriction enzyme cloning using NheI and BamHI. C69S mutant was generated by classical mutagenesis (with following DpnI digest) using the following prime pairs: Forward sfGFP C69S: 5'-*TATGGTGTTCAGTCTTTTGCTCGTTATCCGG*-3'; Revers sfGFP C69S: 5'-taacgagcaaaAGactgaacaccataagtcagc-3'; and succeeding C-terminal AAACA was inserted by Q5 Site Directed Mutagenesis (NEB) using the following primer pairs: Forward sfGFP AAACAstop Q5SDM: 5'-*CTGCGCGTGAGGATCCGAATTCGAG*; Reverse sfGFP AAACAstop Q5SDM: 5'-*GCCGCCGCTTTGTACAGTTCATCCATAC*-3'. Plasmid sequences were confirmed by standard Sanger Sequencing.

The proteins were expressed in BL21(DE3) *E. coli* strain using Luria-Bertanni (LB) medium containing 30 µg/mL Kanamycin (Roth). *E. coli* were grown at 37 °C, until an OD_{600} of 0.6-0.8 was reached, induced with 0.5 mM IPTG, and incubated with shaking at 18 °C overnight. Lysis was performed via sonication (25% amplitude, T_{max} 14 °C, 6 min) in PBS with DNase I and Lysozyme. Debris was centrifuged at 50,000 g for 15 min at 4 °C. The supernatant was reduced with DTT (2 mM, r.t., 30 min) before purification using Ni beads (PureCube 100 Ni-INDIGO agarose). The reduced lysate was loaded to beads equilibrated with PBS containing imidazole (10 mM). This was washed with 10 CV of PBS containing imidazole (20 mM). Protein was eluted with PBS containing imidazole (500 mM). Eluates were collected and subjected to cleavage of the TEV-cleavage site overnight at r.t. in a dialysis bag (3.5 kDa MWCO) in PBS with TEV protease (ratio 1:16 w/w, TEV S219V mutant, David Waugh, Addgene plasmid #8827). Protein solution was reduced with DTT (2 mM, r.t., 30min) before reverse Histag purification (PureCube 100 Ni-INDIGO agarose), purified protein was eluted with PBS containing imidazole (5 mM). Protein was rebuffered to fresh PBS (Vivaspin 20 10k MWCO). The pure protein was isolated in a yield of 70 mg/L. Protein aliquots were stored at -80 °C.

sf-GFP C69S AAACA Sequence:

ATGGGCAGCAGCCATCATCATCATCATCACAGCAGCGGCCTGGTGCCGCGCGG
CAGCCATATGGCTAGCGAAAACCTGTATTTTCAGGGCCGTAAAGGCGAAGAGCT
GTTCACTGGTGTCGTCCCTATTCTGGTGGAACTGGATGGTGATGTCAACGGTCA
TAAGTTTTCCGTGCGTGGCGAGGGTGAAGGTGACGCAACTAATGGTAAACTGAC
GCTGAAGTTCATCTGTACTACTGGTAAACTGCCGGTACCTTGGCCGACTCTGGT
AACGACGCTGACTTATGGTGTTCAGTCTTTTGCTCGTTATCCGGACCATATGAAG
CAGCATGACTTCTTCAAGTCCGCCATGCCGGAAGGCTATGTGCAGGAACGCAC
GATTTCCTTTAAGGATGACGGCACGTACAAAACGCGTGCGGAAGTGAAATTTGA
AGGCGATACCCTGGTAAACCGCATTGAGCTGAAAGGCATTGACTTTAAAGAAGA
CGGCAATATCCTGGGCCATAAGCTGGAATACAATTTTAACAGCCACAATGTTTAC

ATCACCGCCGATAAACAAAAAAATGGCATTAAAGCGAATTTTAAAATTCGCCACA
ACGTGGAGGATGGCAGCGTGCAGCTGGCTGATCACTACCAGCAAAACACTCCA
ATCGGTGATGGTCCTGTTCTGCTGCCAGACAATCACTATCTGAGCACGCAAAGC
GTTCTGTCTAAAGATCCGAACGAGAAACGCGATCATATGGTTCTGCTGGAGTTC
GTAACCGCAGCGGGCATCACGCATGGTATGGATGAACTGTACAAAGCGGCGGC
CTGCGCGTGA

sf-GFP C69S AAACA protein sequence: His-tag highlighted in green; TEV cleavage site highlighted in blue; sf-GFP in black; AAACA highlighted in red.

MGSSHHHHHHSSGLVPRGSHMASENLYFQ▼ GRKGEELFTGVVPILVELDGDVNG
HKFSVRGEGEGDATNGKLTLKFICTTGKLPVPWPTLVTTLTYGVQSFARYPDHMKQ
HDFFKSAMPEGYVQERTISFKDDGTYKTRAEVKFEGDTLVNRIELKGIDFKEDGNIL
GHKLEYNFNSHNVYITADKQKNGIKANFKIRHNVEDGSVQLADHYQQNTPIGDGPVL
LPDNHYLSTQSVLSKDPNEKRDHMVLLEFVTAAGITHGMDELYKAAACA*

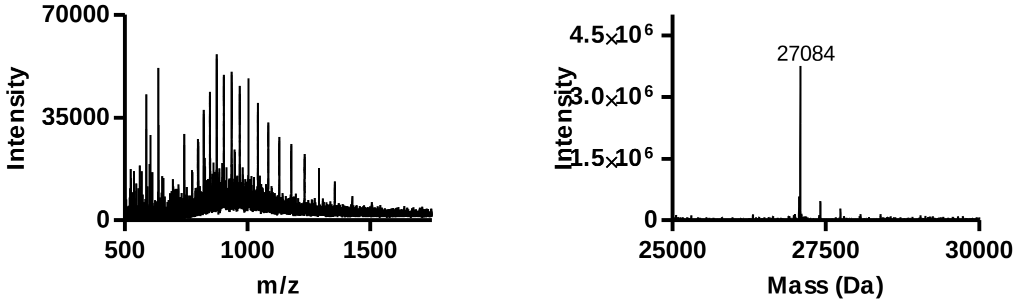

Figure S32: Raw and deconvoluted HR-MS (ESI) spectra of sf-GFP C69S AAACA. Calcd: 27085 Da, found: 27084 Da.

3.2.3 UbG76C expression and purification

Protein was expressed and purified as reported in previous work from the group. The procedure is reproduced here below for ease of access.[6]

The ubiquitin DNA sequence was synthesized by GeneArt AG and cloned into pET28a using NheI and XhoI restriction endonuclease sites. The NdeI restriction site was deleted.

Ubiquitin sequence:
5'GCTAGCCAGATTTTTGTTAAAACCCTGACCGGTAAAACCATTACCCTGGAAGTT
GAACCGAGCGATACCATTGAAAATGTGAAAGCCAAAATCCAGGACAAAGAAGGT
ATTCCGCCTGATCAGCAGCGTCTGATTTTTGCAGGTAAACAGCTGGAAGATGGT
CGTACCCTGAGCGATTATAACATTCAGAAAGAAAGCACCCTGCATCTGGTTCTG
CGTCTGCGTGGTGGT 3'

The mutations were introduced with classical PCR by the use of complementary primer pairs;

UbG76C
fwd: 5' GTGCTCGAGTTAGCAACCACGCAGACG 3'
rev: 5' CGTCTGCGTGGTTGCTAACTCGAGCAC 3'

The proteins were expressed in E. coli B834(DE3) using LB medium containing 30 µg/mL Kanamycin (Roth). Cells were grown at 37 °C, 180 rpm until an OD_{600} of 0.6-0.8 was reached, induced with 1 mM IPTG and incubated at 18 °C for 19 h. Lysis was performed in Dulbecco's PBS pH 7.4 using a high-pressure homogenizer (Microfluidics LM10 Microfluidizer) and debris centrifuged at 50.000 g for 15 min at 4 °C. The protein was purified with a BioRad NGC system (BioRad, USA) using a 5 mL HisTrap FF column (GE Healthcare, USA). Product-containing fractions were collected and dialyzed (MWCO: 3.5 kDa, Spectra/Pro®3 dialysis membrane) into the Thrombin cleavage buffer (20 mM Tris·HCl pH 8.5, 150 mM NaCl, 2.5 mM $CalCl_2$). Then Thrombin was added (5 u/mL) and samples were incubated at 37 °C for 18 h. A second Ni-NTA purification was performed with a BioRad NGC system (BioRad, USA) using a 5 mL HisTrap FF (GE Healthcare, USA) column and the flow-through was collected and dialyzed (MWCO: 3.5 kDa, Spectra/Pro®3 dialysis membrane) into Dulbecco's PBS pH 7.4. The protein concentration was determined by NanoDrop® at 280 nm (ε = 1490 M^{-1} cm^{-1}). The pure protein was isolated in a yield of 20 mg/L. Protein aliquots were stored at -80 °C.

UbG76C Protein sequence: His-tag highlighted in green; protease cleavage site highlighted in blue; UbG76C in black; G76C highlighted in red.
MGSSHHHHHHSSGLVPRGSASQIFVKTLTGKTITLEVEPSDTIENVKAKIQDKEGIPP
DQQRLIFA GKQLEDGR TLSDYNIQKESTLHLVLRLRGC

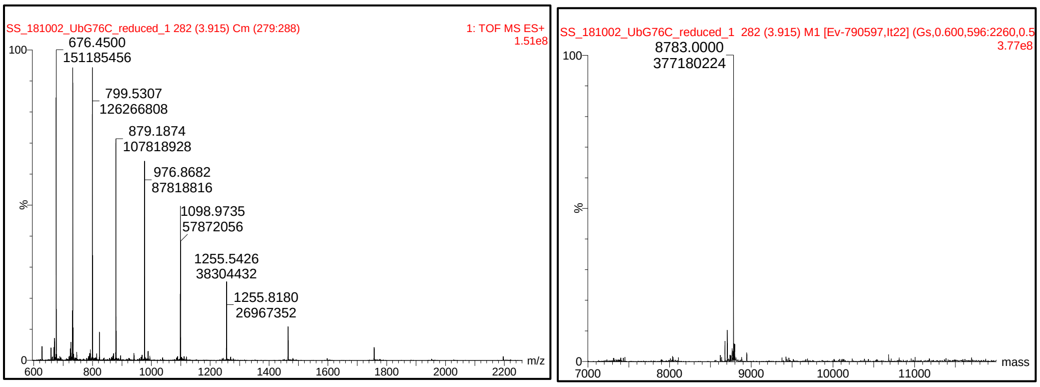

Figure S33: Raw and deconvoluted HR-MS (ESI) spectra of UbG76C 15 after DTT reduction. Calcd: 8782Da, found: 8783 Da.

3.3 Fluorescence Microscopy

3.3.1 Cell culture

SKBR-3 and MDA-MB-468 cells were cultivated in DMEM/ F12 supplemented with 10 % fetal bovine serum (FBS) and 2 mM glutamine, and HeLa cells were cultured in DMEM high glucose with 10% FCS at 37 °C in a humidified atmosphere with 5 % CO2 and used for experiments in passages 3-15. For the immunofluorescence experiment, 30’000 cells of each cell line were seeded in 4 wells each of an 8-well glass bottom chambered coverslip (ibidi GmbH).

3.3.2 Microscopy experiments

Cells were incubated with 50 nM labeled antibody (**Tras-4**) in 150 µL of Fluorobrite DMEM without FCS and glutamine. For each cell line, one well was incubated with Fluorobrite DMEM without FCS and glutamine as a control. After 1 h incubation at 37 °C cells were washed thrice with Fluorobrite DMEM with 10% FCS and 2 mM glutamine and counterstained with Hoechst 33342. After 15 min cells were washed and left in Fluorobrite DMEM with 10% FCS and 2 mM glutamine.

For the cell uptake experiment, HeLa cells were seeded onto 8-well glass bottom chambered slides (IBIDI) at 30,000 cells/well density. After allowing the cells to attach for 48 h, the cells were washed twice with PBS, and treated for 1 h with mCherry or mCherry-**P6** at the indicated concentration in main text Figure 4c in the presence of 5 µM TNB-R10-ILFF. Treated cells were washed twice with PBS and counterstained the nucleus with Hoechst 33342

Live cell microscopy images of the labeled antibody (green) were acquired on a Nikon-CSU spinning disc microscope with a CSU-X1 (Andor) and live cell incubation chamber (OKOlab). All live cell images were acquired using a PlanApo 60x NA 1.4 oil objective (Nikon) and an EMCCD (AU888, Andor). Brightfield images were acquired along with fluorescence images. Standard laser, a quad Dicroic (400-410, 486-491, 560-570, 633-647, AHF) and Emission filters were used for the acquisition of confocal fluorescence images (BFP (Hoechst 33342) ex.: 405 nm em.: 450/50 nm, GFP (antibody), ex.: 488 nm em.: 525/50 nm, RFP (mCherry), ex.: 561 em.:600/50 nm).

3.4 Cell Based Anti-Proliferation Assays

SKBR-3 and MDA-MB-468 cell lines were cultured in DMEM/F12 medium supplemented with 10 % FCS, 2 mM glutamine and 1X Penicillin-Streptomycin (Penicillin 10^5 units/L; Streptomycin 100 mg/L). Cells were seeded (100 µL) at a density of $5*10^3$ cells/well (SKBR3) or $2*10^3$ cells/well (MDA-MB-468) in black 96-well cell culture microplates (Costar). Plates were incubated for 24 h at 37 °C, 5 % CO_2. Subsequently, the cells were aspirated and respective wells on the microplate were directly subjected to 1:4 serial dilutions of ADCs / antibodies in medium (5 % PBS; 100 µL) starting at 50 nM final concentration. Plates were incubated for 96 h at 37 °C, 5 % CO_2. Subsequently, the cells were aspirated and resazurin (100 µM in medium; 100 µL) was added, followed by incubation for 4 h at 37 °C, 5 % CO_2. Metabolic conversion of resazurin to resorufin was quantified by the fluorescent signal of resorufin (λ_{EX} = 560 nm, λ_{EM} = 590 nm, excitation bandwidth = 9 nm, emission bandwidth = 20 nm, gain (manual) = 50, number of flashes = 25, integration time = 20 µs, lag time = 0 µs, settle time = 0 ms, Z-position (manual) = 20000 µm) on a Tecan Infinite 200 Pro microplate reader. Data analysis was performed with Graphpad Prism 10 software. Raw data was normalized to 0 % viability (cells treated with 10 µM MMAE in medium with 5 % PBS; 100 µL) and 100 % viability (cells treated with medium with 5 % PBS; 100 µL). Mean and standard error of the mean (SEM) were calculated from four biological replicates which all contained triplicate datasets (N = 4, n = 3) and plotted against ADC/antibody concentration. IC_{50} values were calculated using a nonlinear regression (log inhibitor vs response).

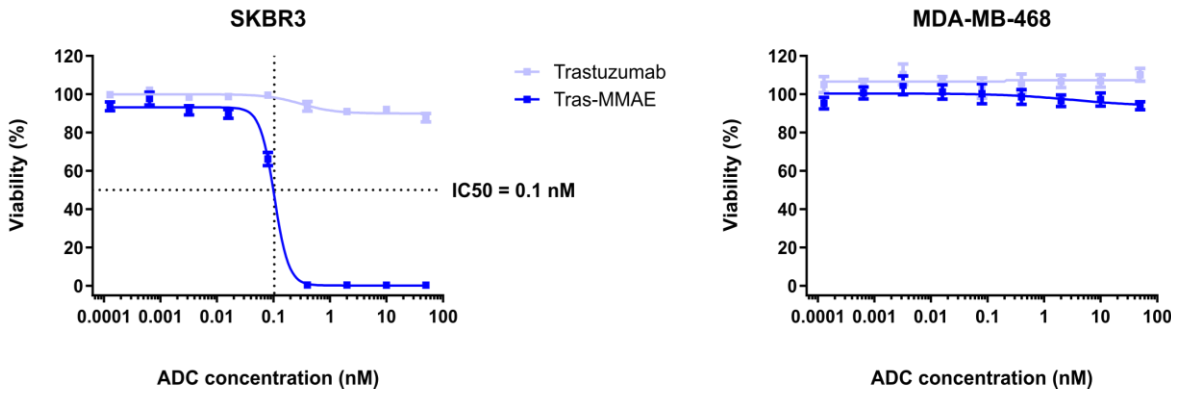

3.5 Determination of the reaction kinetics of substituted ethynyl-ditriazolyl-phosphinates (EDPO):

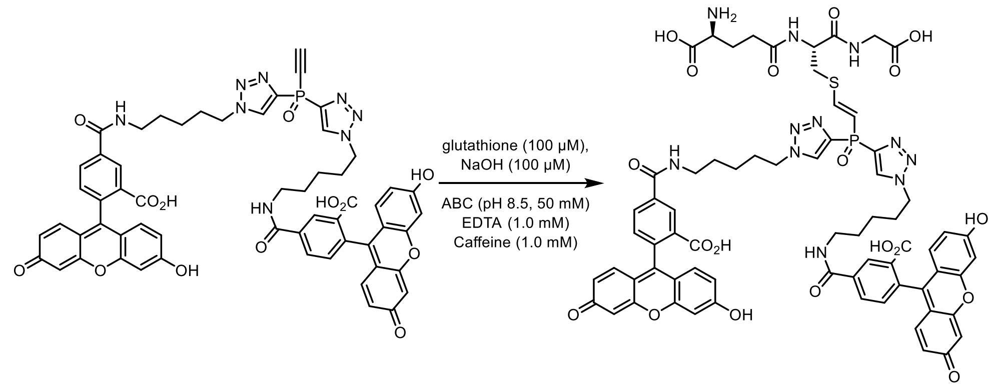

To a shaken solution of EDPO **4a** (2 µL of 5 mM stock in DMSO, 10 µmol, 1 equiv.) in ABC buffer (88 µL, 50 mM, pH 8.5) containing EDTA (1 mM) and caffeine (1 mM) at 25 °C was added a solution of reduced glutathione (10 µL of 1 mM stock in ABC buffer (50 mM, pH 8.5), 10 µmol, 1 equiv.) containing NaOH (1 mM, 10 µmol, 1 equiv.). The timer was started immediately upon glutathione addition. Samples were prepared by diluting drawn reaction mixture (5 µL), with aqueous TFA solution (30 µL, 20 mM) to stop the reaction. Samples were then submitted to UPLC analysis (gradient A). The first sample (t = 0) was drawn before glutathione addition. The following samples were drawn after 1, 3, 5, 10, 25 and 40 minutes respectively.

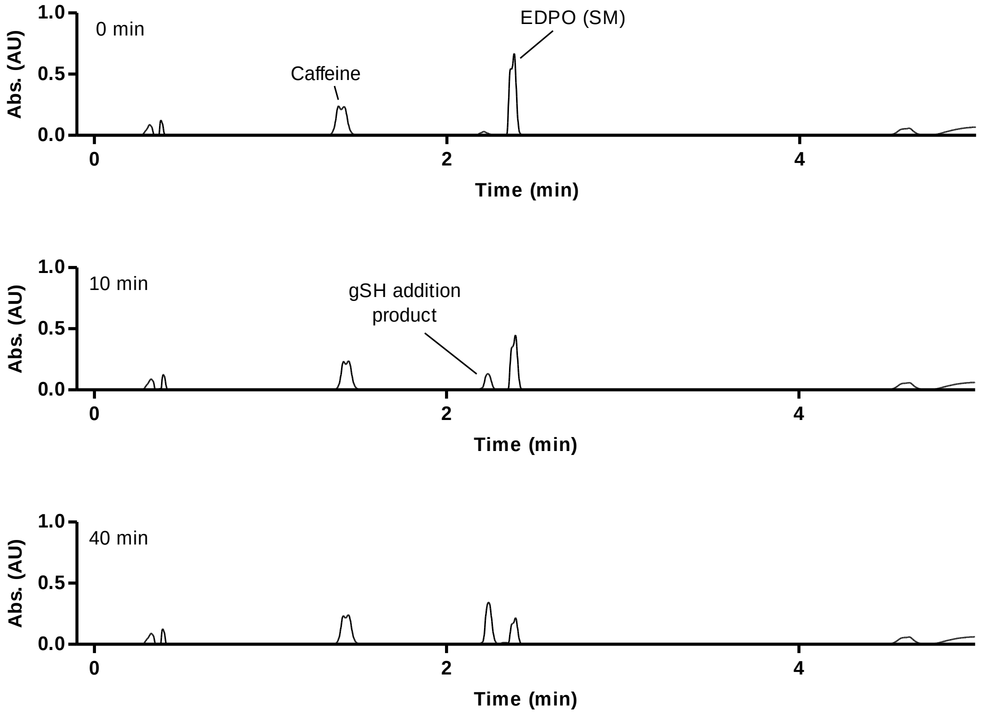

Figure S34: Exemplary HPLC traces of the reaction at pH 8.5. Starting material **4a** converts cleanly into the glutathione addition product.

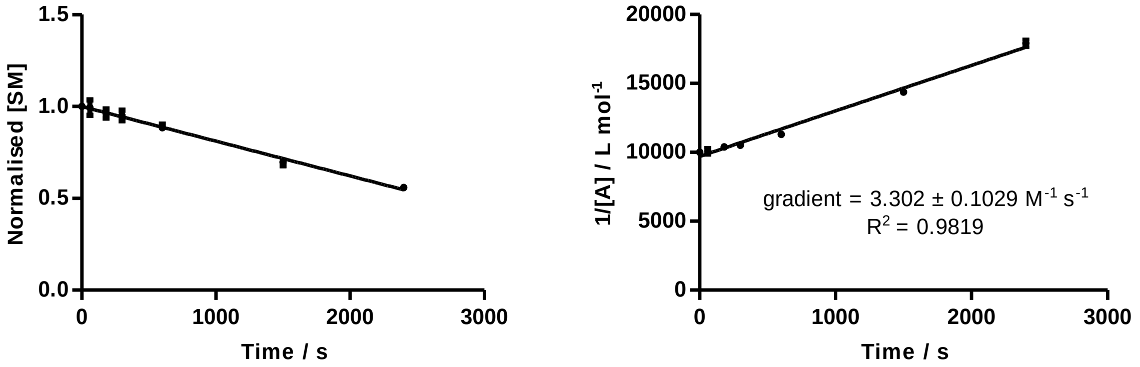

Figure S35: Concentration of **4a** over time, calculated by integration of the UV-peaks in relation to the internal standard (*caffeine*). Shown are mean and error (SD) values of three independent measurements (n = 3). Right: 1/concentration of **4a** over time for reaction and linear plot. The slope of the linear plot is the second order rate constant according to the second order integrated rate law $1/[A] = 1/[A]_0 + kt$ ([A] = [B]).

3.6 Determination of EDPO-conjugate stability

To a solution of caffeine (2 µL of 20 mM water stock, 40 µmol) or triphenylphosphine oxide (1 µL of 2 mM stock in DMSO, in the case of the glutathione experiment) in the tested buffer (98 µL) was added **4a-LYRCAK** (1 µL in 1 mM DMSO stock, 1 µmol). The timer was started immediately upon addition of **4a-LYRCAK**. Aliquots (5 µL) of the reaction mixture were directly injected into the UPLC (gradient C) at t = 0, 30, 60, 120, 240, 480, 960 and 1440 minutes. The normalized ratio of **4a-LYRCAK** to caffeine was plotted against time for each condition tested. The conditions tested were as follows: pH 7.4 PBS, pH 8.5 PBS, 0.1% TFA (v/v) and 10 mM glutathione in pH 7.4 PBS.

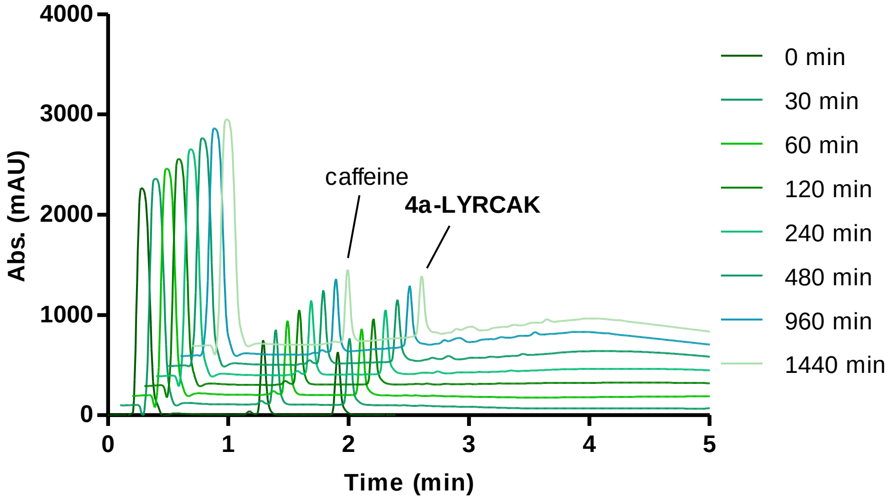

Figure S36: Representative UPLC plot showing the caffeine and **4a-LYRCAK** peaks of one representative replicate in PBS (pH 8.5).

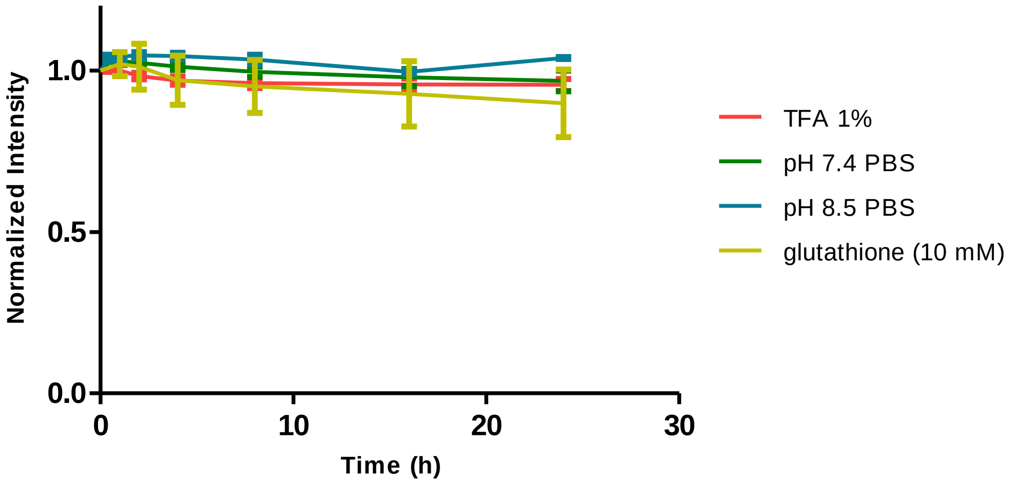

Figure S37: Plot of the remaining starting material vs time for various conditions. Shown are mean and error (SD) values of three independent measurements (n = 3).

4 Organic Synthesis

4.1 General synthesis

4.1.1 Bis-ethynyl(phenyl)phosphine oxide (Compound 1):

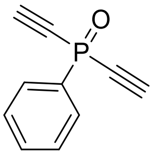

180 mg dichloro phenyl-phosphine were dissolved in 2 ml dry THF under argon. The solution was cooled -78 °C, 2.2 eq. ethynyl magneisum bromide (0.5 M in THF, 1.1 eq.) were added dropwise and the reaction was allowed to warm to room temperature. Then the mixture was cooled to 0 °C and 3 eq. hydrogen peroxide (30 % aq. solution) were added dropwise. The reaction mixture was allowed to warm to room temperature. Subsequently the mixture was extracted from H_2O/DCM. Combined organic layers were dried and evaporated under reduced pressure. The crude product was purified over silica (EtOAc) to afford the title compound in 40 % yield (70 mg).

^{1}H NMR (600 MHz, DMSO-d_6) δ 7.98 – 7.83 (m, 2H), 7.80 – 7.70 (m, 1H), 7.66 (td, *J* = 7.6, 3.7 Hz, 2H), 5.00 (d, *J* = 11.1 Hz, 2H).
^{13}C NMR (151 MHz, DMSO-d_6) δ 133.98 (d, *J* = 3.2 Hz), 131.77 (d, *J* = 140.6 Hz), 130.29 (d, *J* = 12.7 Hz), 129.77 (d, *J* = 14.8 Hz), 97.72 (d, *J* = 34.0 Hz), 78.85 (d, *J* = 189.8 Hz).
^{31}P NMR {^{1}H} (243 MHz, DMSO-d_6) δ -22.79.
HRMS for $C_{10}H_8OP^+$ $[M+H]^+$ calc.: 175.0307 Da; found: 175.0316 Da

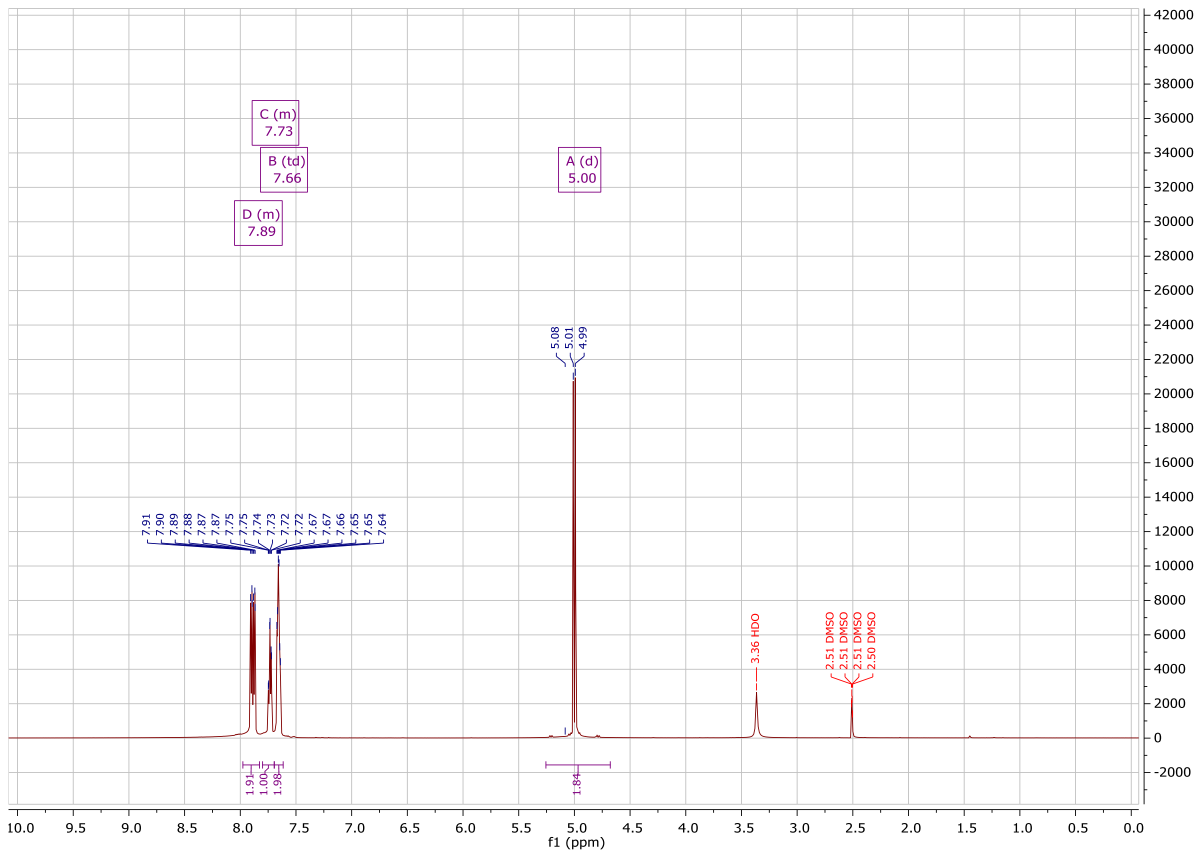
C (m)
7.73
B (td)
7.66
D (m)
7.89
A (d)
5.00
3.36 HDO
2.51 DMSO
2.51 DMSO
2.51 DMSO
2.50 DMSO
f1 (ppm)

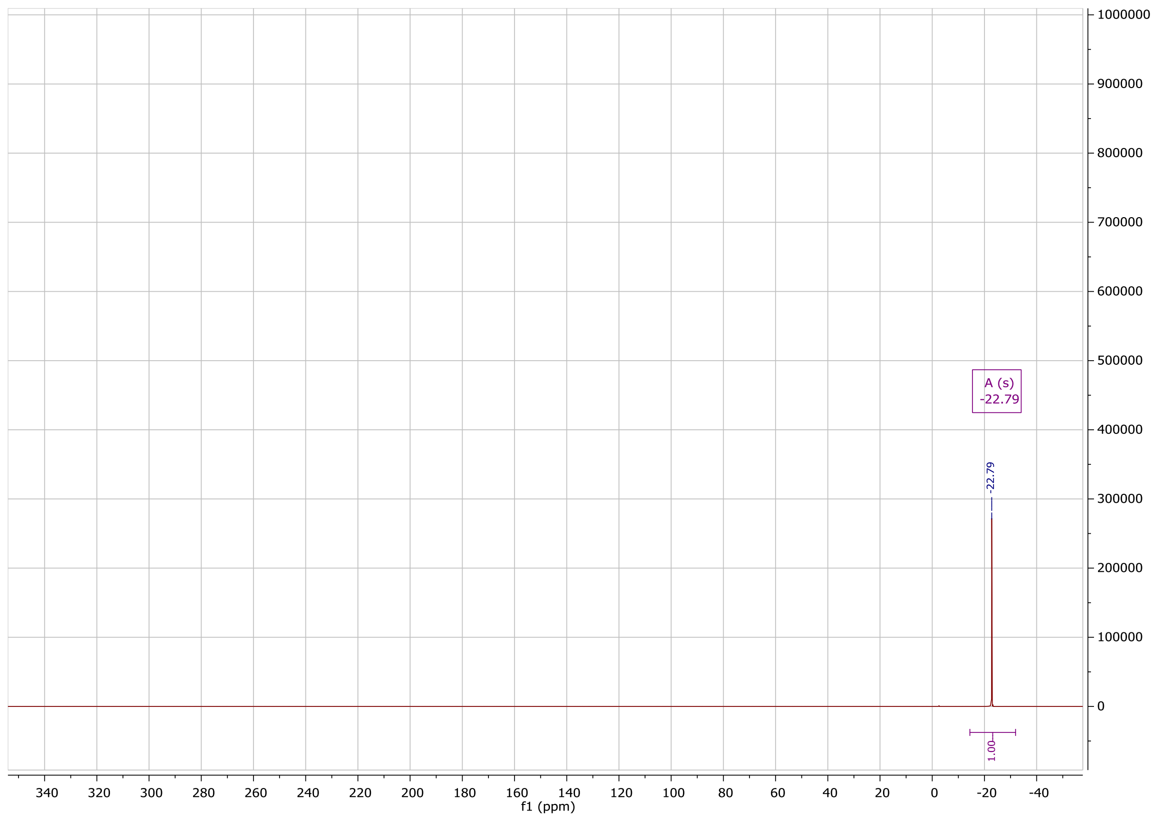
A (s)
-22.79
f1 (ppm)

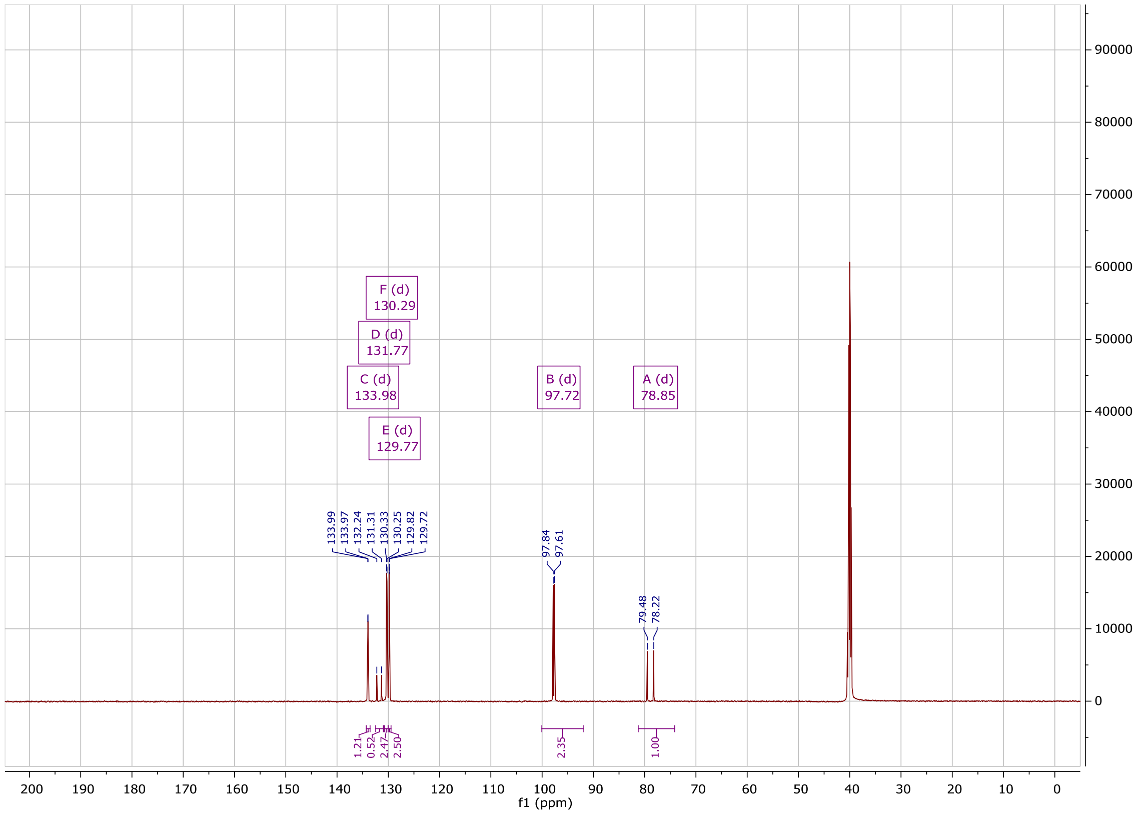

F (d)
130.29
D (d)
131.77
C (d)
133.98
E (d)
129.77
B (d)
97.72
A (d)
78.85
133.99
133.97
132.24
131.31
130.33
130.25
129.82
129.72
97.84
97.61
79.48
78.22
1.21
0.52
2.47
2.50
2.35
1.00
f1 (ppm)

4.1.2 **Triethynylphosphine oxide (Compound 2):**

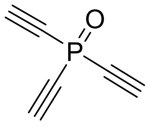

To a 0 °C solution of TMS-acetylene (969 µL, 7 mmol, 3.5 equiv.) in THF (5 mL, anhydrous, degassed) under argon was added dropwise EtMgBr (1 M in THF, 6 mL, 6 mmol, 3 equiv.). The reaction was allowed to warm to room temperature to form the TMS-acetylene Grignard reagent. In a separate flask phosphorylchloride (182 µL, 2 mmol, 1 equiv.) was dissolved in THF (4 mL, anhydrous, degassed) under argon and cooled to -78 °C. The Grignard reagent from flask 1 was added dropwise to the second flask and the reaction was allowed to warm to room temperature. The crude reaction mixture was diluted with distilled water (50 mL) and extracted with DCM (3x30 mL) Combined organic layers were dried over $MgSO_4$, filtered and evaporated under reduced pressure until approx. 10% of the solvent remained. THF (20 mL) and H_2O (2 mL) were then added, followed by silica-loaded ammonium fluoride (250 mg, 6.7 mmol, 3.4 equiv. on 4.5 g silica, 1.5 mmol/g). When TLC-analysis indicated complete TMS-removal, the crude reaction mixture was purified over a silica plug, washing first with pentane and then eluting with pentane/EtOAc (1:1) to obtain the title compound as a colorless solid. (170 mg, 69.1%).

^{1}H NMR (600 MHz, $CDCl_3$) δ 3.34 (d, *J* = 12.5 Hz, 3H).
^{13}C NMR (151 MHz, $CDCl_3$) δ 92.3 (d, *J* = 45.6 Hz), 77.6 (d, *J* = 234.0 Hz).
^{31}P-NMR {^{1}H} (243 MHz, $CDCl_3$) δ -55.3.
HRMS for $C_6H_4OP^+$ [M+H$^+$] calc.: 122.9994 Da; found: 123.0009 Da.

Notes:

- The compound was colorless immediately after purification but slowly turns light pink and then brown over time.
- The compound seems to decompose slightly during the purification (likely from the heat of rotary evaporation).
- The compound was stored at 5 °C when not in use where it remained in reasonable purity after several months.

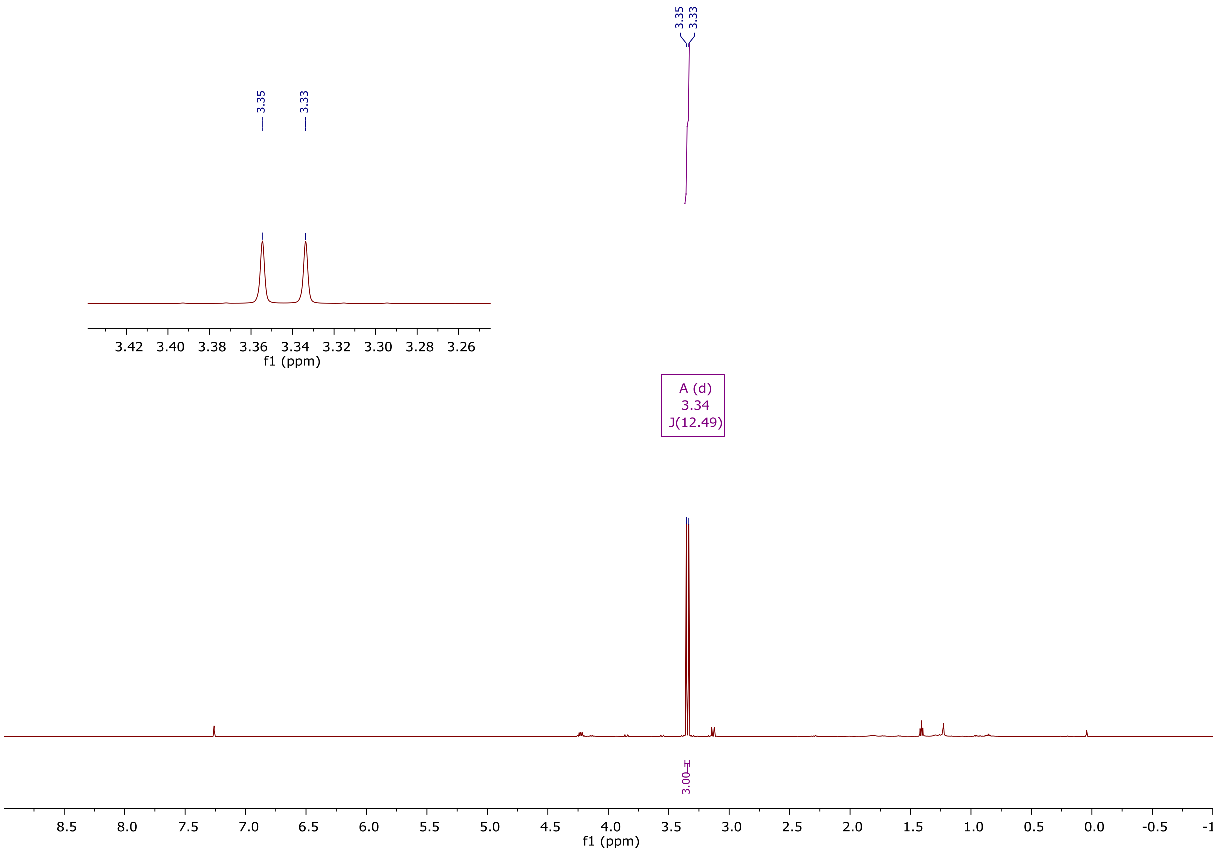

3.35
3.33
3.42 3.40 3.38 3.36 3.34 3.32 3.30 3.28 3.26
f1 (ppm)
A (d)
3.34
J(12.49)
3.00
8.5 8.0 7.5 7.0 6.5 6.0 5.5 5.0 4.5 4.0 3.5 3.0 2.5 2.0 1.5 1.0 0.5 0.0 -0.5
f1 (ppm)

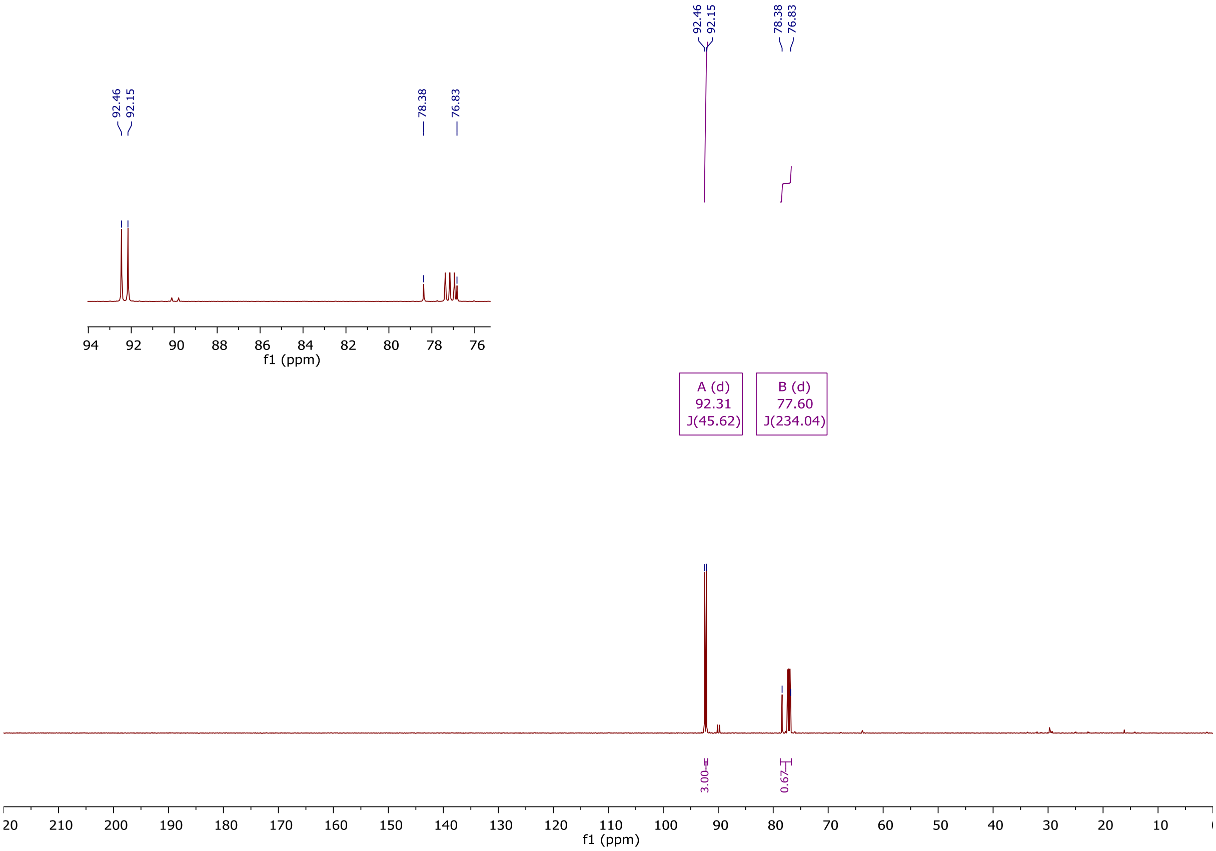

92.46
92.15
78.38
76.83
94 92 90 88 86 84 82 80 78 76
f1 (ppm)
A (d)
92.31
J(45.62)
B (d)
77.60
J(234.04)
3.00
0.67
210 200 190 180 170 160 150 140 130 120 110 100 90 80 70 60 50 40 30 20 10
f1 (ppm)

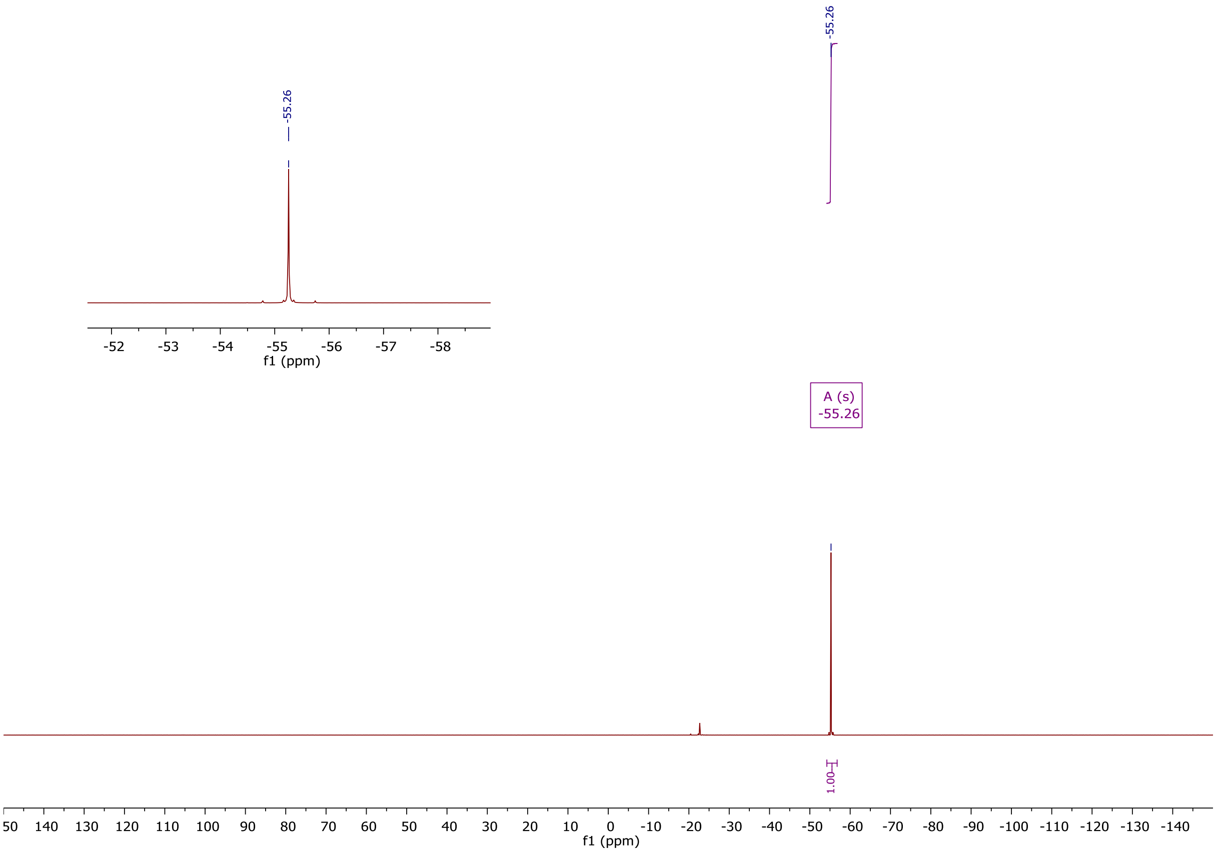

-55.26
-52
-53
-54
-55
-56
-57
-58
f1 (ppm)
A (s)
-55.26
1.00
150
140
130
120
110
100
90
80
70
60
50
40
30
20
10
0
-10
-20
-30
-40
-50
-60
-70
-80
-90
-100
-110
-120
-130
-140
f1 (ppm)

4.1.3 Ethyl diethynyl phosphine oxide (Compound 3):

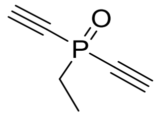

^{1}H NMR (600 MHz, Chloroform-*d*) δ 3.17 (d, *J* = 10.5 Hz, 2H), 2.11 (dq, *J* = 15.3, 7.6 Hz, 2H), 1.33 (dt, *J* = 23.7, 7.6 Hz, 3H).
^{13}C NMR (151 MHz, Chloroform-*d*) δ 91.56 (d, *J* = 32.8 Hz), 77.12 (d, *J* = 31.9 Hz), 27.70 (d, *J* = 97.6 Hz), 5.63 (d, *J* = 4.8 Hz).
^{31}P NMR {^{1}H} (243 MHz, Chloroform-*d*) δ -7.04.
HRMS for $C_6H_8OP^+$ [M+H$^+$] calc.: 127.0307 Da; found: 127.0310 Da.

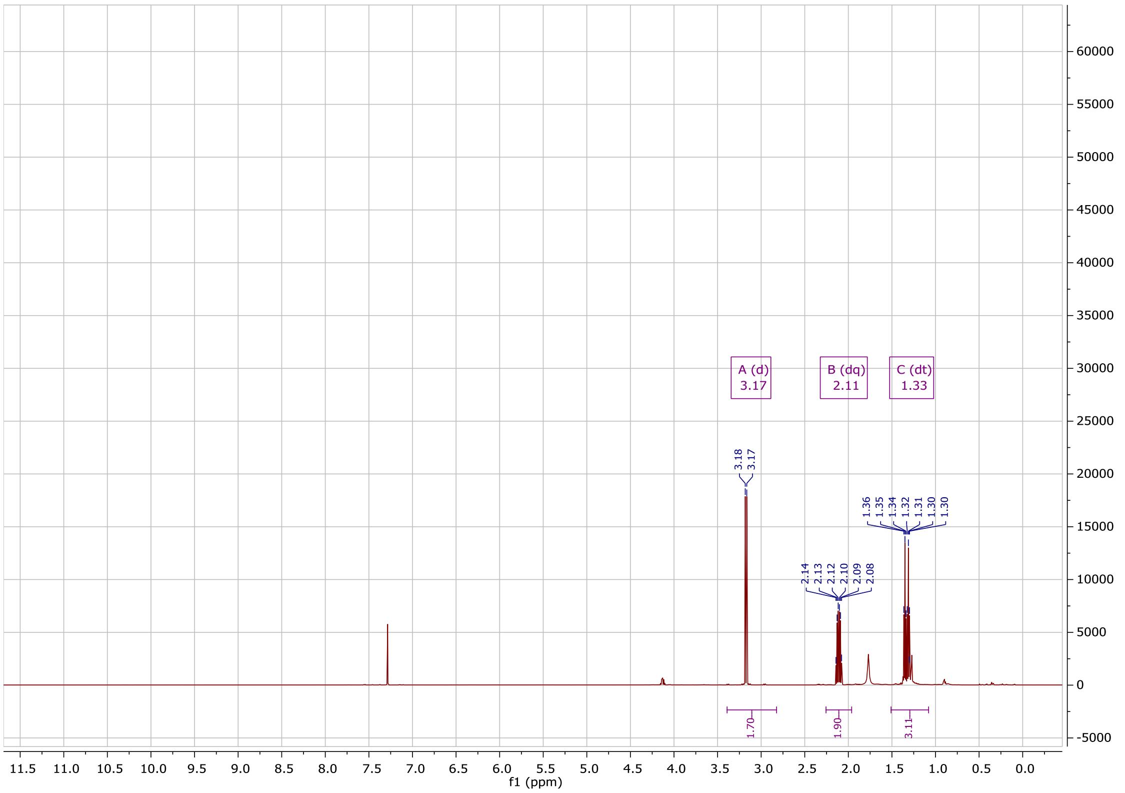

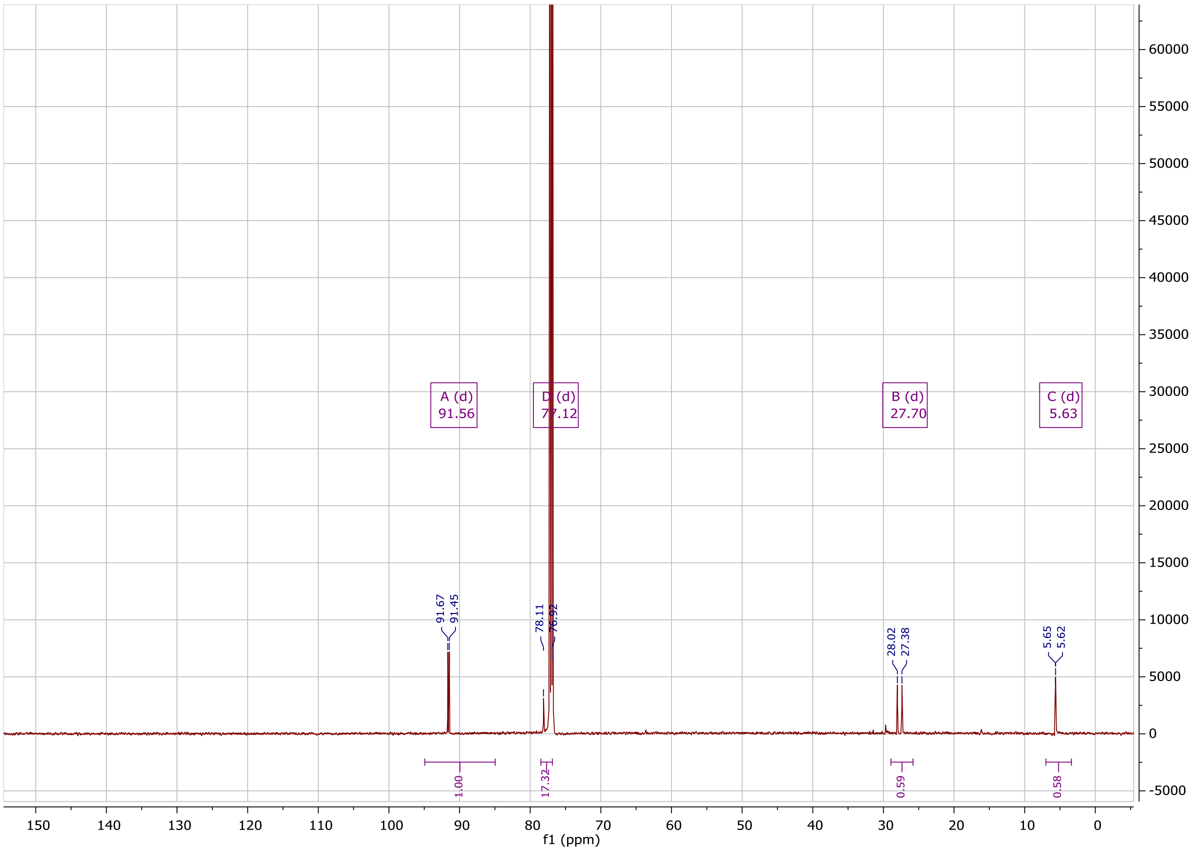
A (d)
91.56
D (d)
77.12
B (d)
27.70
C (d)
5.63
91.67
91.45
78.11
76.92
28.02
27.38
5.65
5.62
1.00
17.32
0.59
0.58
f1 (ppm)

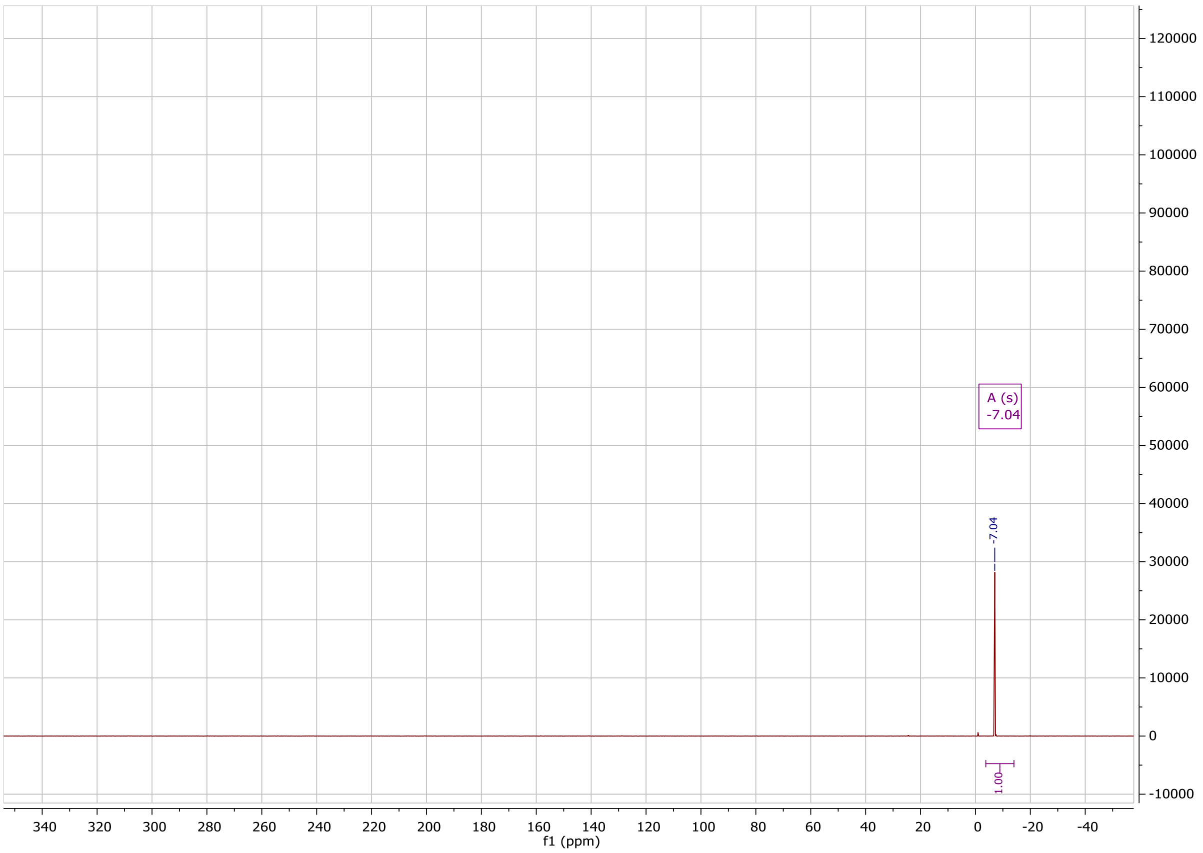
A (s)
-7.04
-7.04
1.00
f1 (ppm)

4.1.4 Sulfo-Rhodamine-B-PEG$_4$-N$_3$ (Compound 6a):

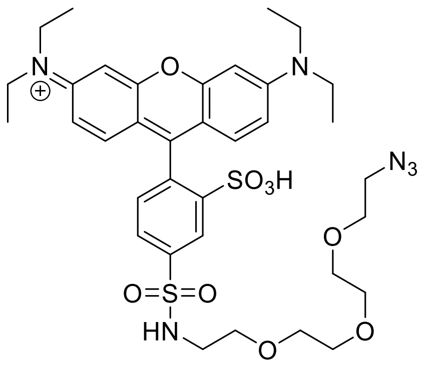

^{1}H NMR (600 MHz, Acetonitrile-d_3) δ 8.57 (d, J = 1.9 Hz, 1H), 8.04 (dd, J = 7.9, 1.9 Hz, 1H), 7.14 (d, J = 9.5 Hz, 2H), 6.95 (dd, J = 9.5, 2.5 Hz, 2H), 6.84 (d, J = 2.4 Hz, 2H), 3.70 – 3.57 (m, 16H), 3.60 – 3.51 (m, 4H), 3.37 (t, J = 5.0 Hz, 2H), 3.18 (t, J = 5.4 Hz, 2H), 1.27 (t, J = 7.2 Hz, 12H).
^{13}C NMR (151 MHz, Acetonitrile-d_3) δ 157.82, 157.33, 155.64, 147.19, 142.00, 133.82, 132.71, 130.73, 127.47, 126.24, 113.96, 113.55, 95.53, 70.18, 70.15, 70.08, 69.96, 69.51, 69.16, 50.47, 45.57, 42.86, 11.75.
HRMS for $C_{35}H_{47}N_6O_9S_2^+$ [M]$^+$ calc.: 759.2840 Da; found: 759.2658.

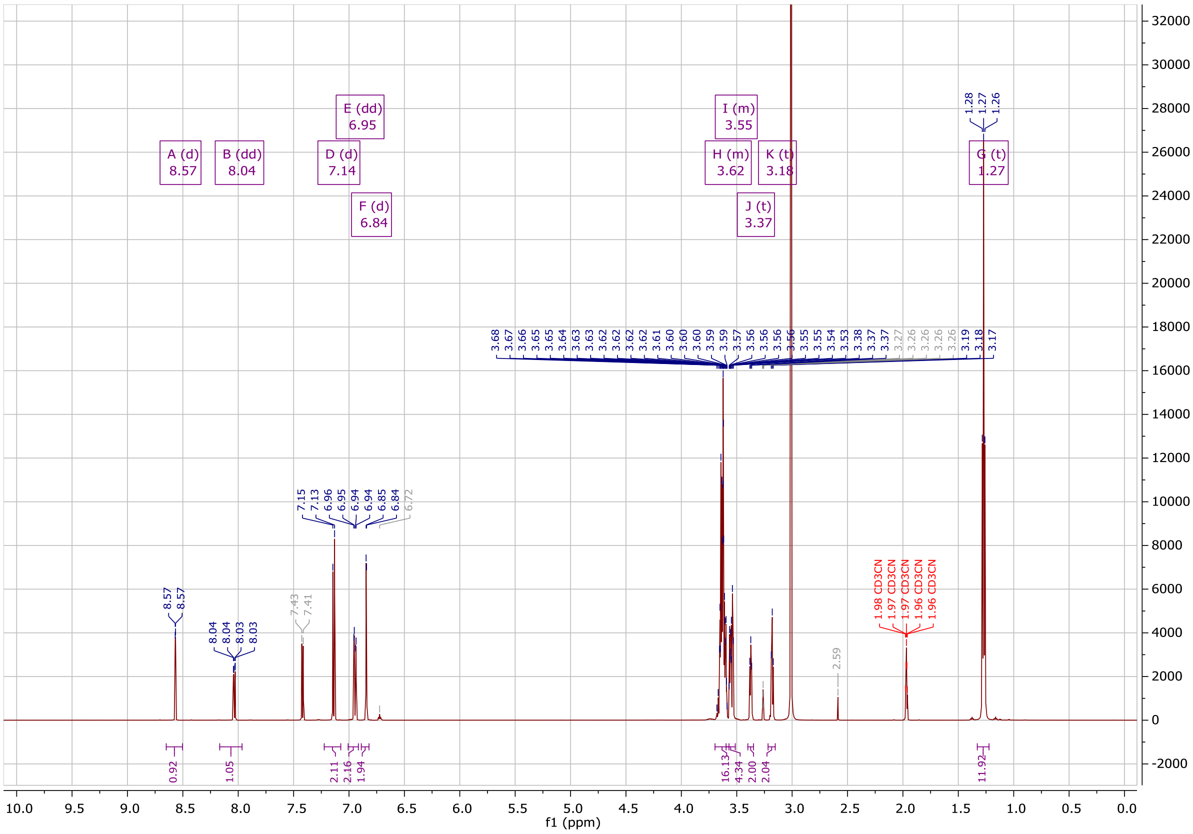
A (d) 8.57
B (dd) 8.04
D (d) 7.14
E (dd) 6.95
F (d) 6.84
G (t) 1.27
H (m) 3.62
I (m) 3.55
J (t) 3.37
K (t) 3.18
1.98 CD3CN
1.97 CD3CN
1.97 CD3CN
1.96 CD3CN
1.96 CD3CN
f1 (ppm)

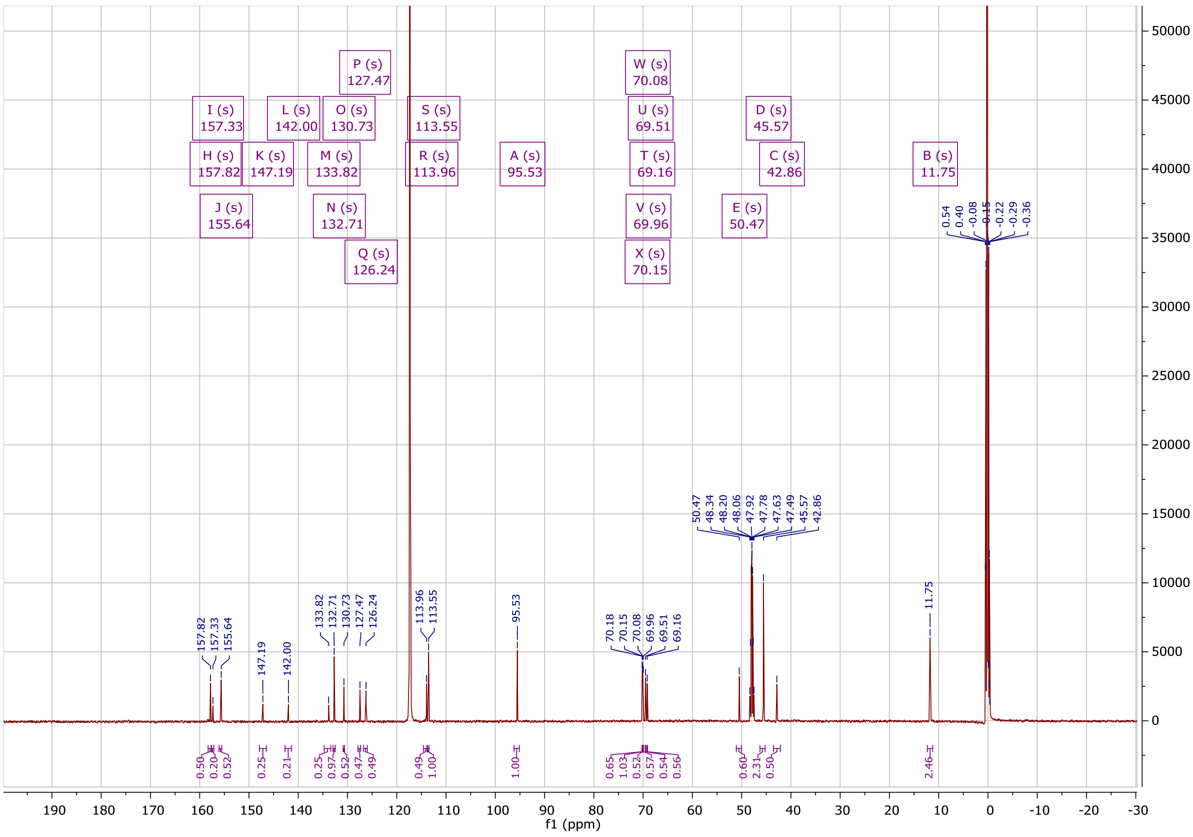
A (s) 95.53
B (s) 11.75
C (s) 42.86
D (s) 45.57
E (s) 50.47
H (s) 157.82
I (s) 157.33
J (s) 155.64
K (s) 147.19
L (s) 142.00
M (s) 133.82
N (s) 132.71
O (s) 130.73
P (s) 127.47
Q (s) 126.24
R (s) 113.96
S (s) 113.55
T (s) 69.16
U (s) 69.51
V (s) 69.96
W (s) 70.08
X (s) 70.15
f1 (ppm)

4.1.5 **4-(6-methyl-1,2,4,5-tetrazin-3-yl)phenol (Compound 7a):**

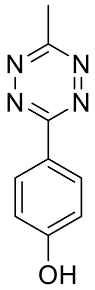

Synthesized according to Mao *et al.*[7] from 4-hydroxy-benzonitrile (4 mmol, 476 mg) and acetonitrile (1.7 ml, 32 mmol). The compound was purified via column chromatography over silica (EtOAc) to obtain the title compound as a pink solid in 20% yield (152 mg).

^{1}H NMR (600 MHz, Acetonitrile-d_3) δ 8.46 – 8.38 (m, 2H), 7.07 – 6.98 (m, 2H), 2.99 (s, 3H).
^{13}C NMR (151 MHz, Acetonitrile-d_3) δ 166.77, 163.66, 160.90, 129.55, 126.45 – 120.85 (m), 116.60 – 114.40 (m), 20.27.

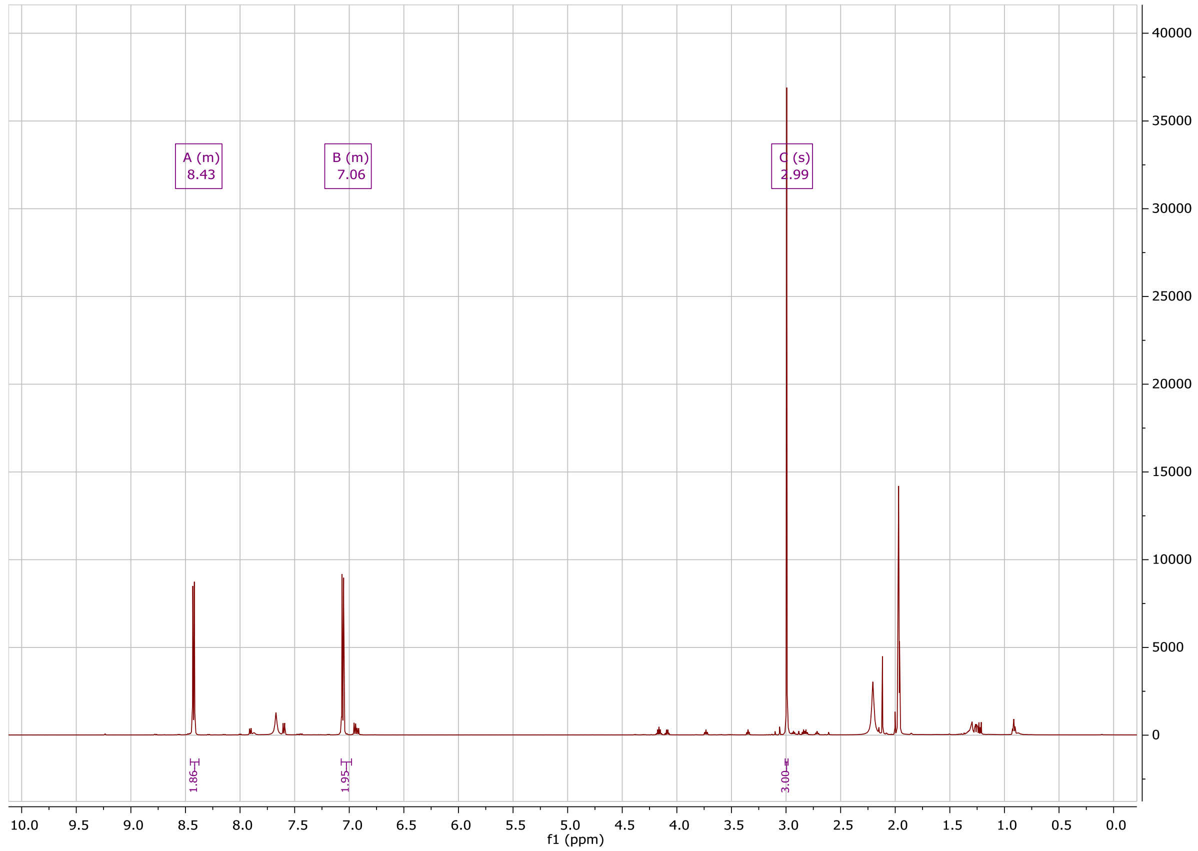
A (m)
8.43
B (m)
7.06
C (s)
2.99
1.86
1.95
3.00
f1 (ppm)

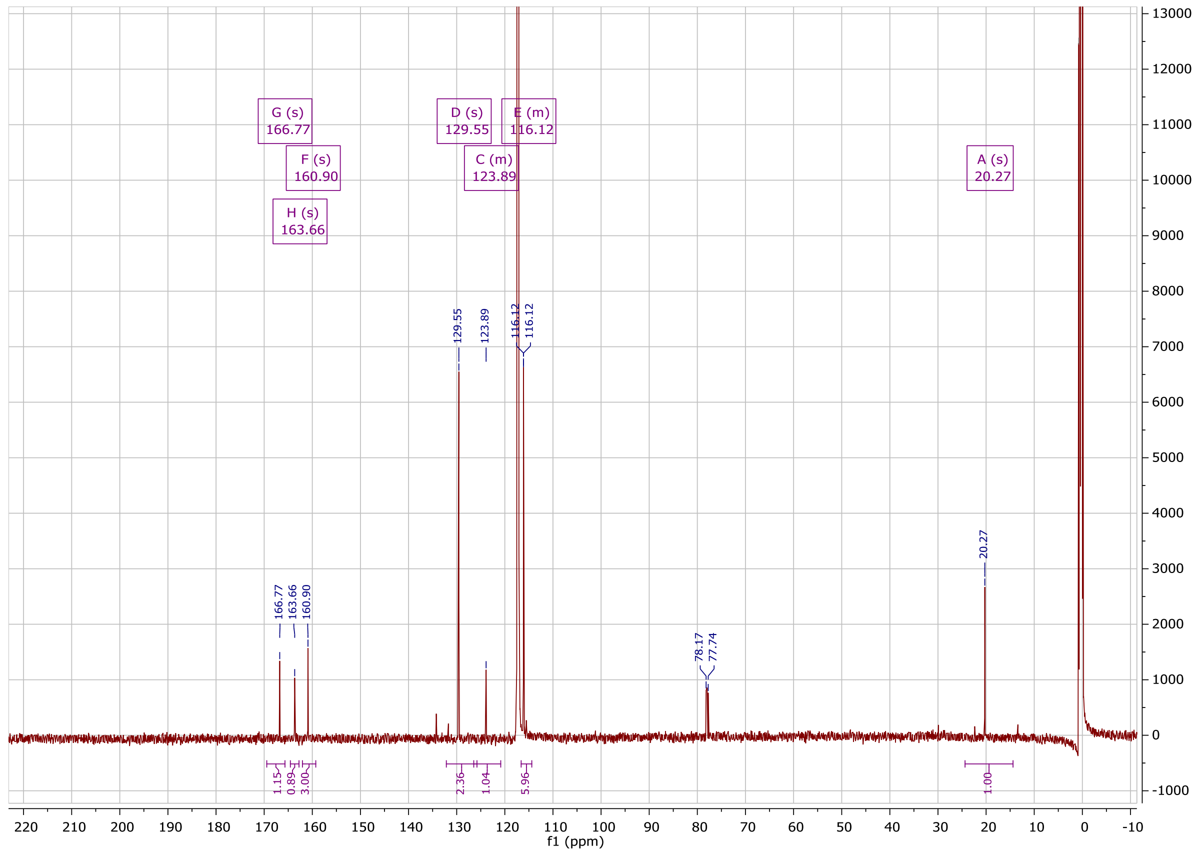
G (s)
166.77
F (s)
160.90
H (s)
163.66
D (s)
129.55
E (m)
116.12
C (m)
123.89
A (s)
20.27
166.77
163.66
160.90
129.55
123.89
116.12
116.12
78.17
77.74
20.27
1.15
0.89
3.00
2.36
1.04
5.96
1.00
f1 (ppm)

4.1.6 **3-(4-(2-(2-(2-azidoethoxy)ethoxy)ethoxy)phenyl)-6-methyl-1,2,4,5-tetrazine (Compound 7b):**

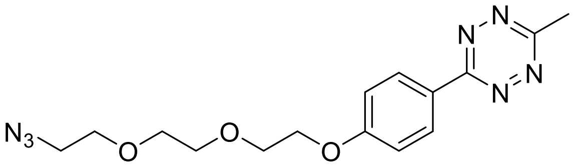

0.7 mmol **7a** were dissolved in 15 ml DMF/MeCN (5:1). 2 eq. K_2CO_3 and 1.2 eq. Tosyl-PEG_3-N_3 were added and the reaction was stirred at 60 °C for 2 h. The mixture was extracted from 1M HCl (100 ml) and EtOAc (3x 50 ml), the combined organic extracts were dried over $MgSO_4$, filtered and evaporated. The obtained crude was purified over silica (Hexane/EtOAC 2:1) to obtain 183 mg of a pink solid. (76% yield).

^{1}H NMR (600 MHz, Acetonitrile-d_3) δ 8.48 (dt, J = 8.9, 2.0 Hz, 2H), 7.19 – 7.13 (m, 2H), 4.28 – 4.22 (m, 2H), 3.89 – 3.84 (m, 2H), 3.71 – 3.68 (m, 2H), 3.68 – 3.64 (m, 4H), 3.39 (q, J = 4.4, 3.9 Hz, 2H), 3.00 (s, 3H).
^{13}C NMR (151 MHz, Acetonitrile-d_3) δ 166.88, 163.58, 162.42, 129.37, 124.68, 115.33 (d, J = 19.0 Hz), 70.39 (d, J = 4.2 Hz), 70.19, 69.57, 69.19, 67.82, 50.53, 20.29.

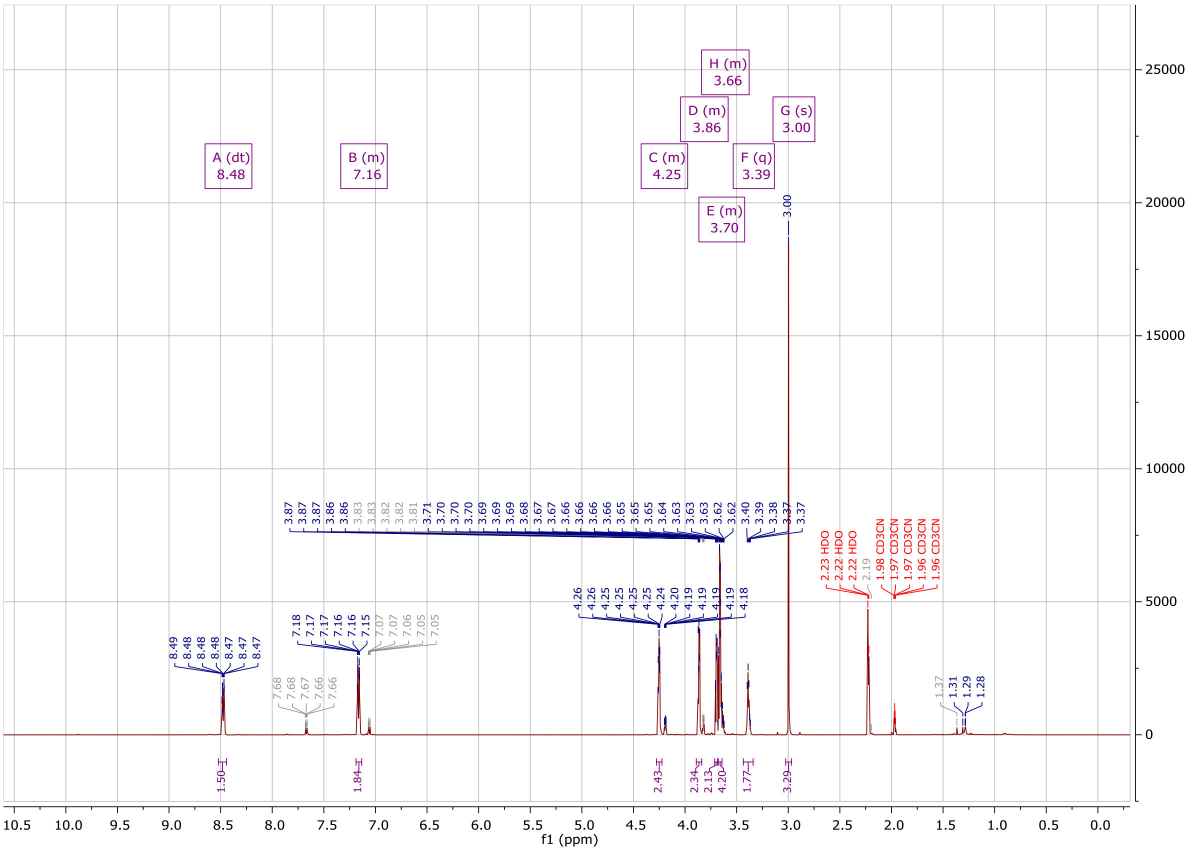

H (m)
3.66
D (m)
3.86
G (s)
3.00
A (dt)
8.48
B (m)
7.16
C (m)
4.25
F (q)
3.39
E (m)
3.70
2.23 HDO
2.22 HDO
2.22 HDO
1.98 CD3CN
1.97 CD3CN
1.97 CD3CN
1.96 CD3CN
1.96 CD3CN
f1 (ppm)

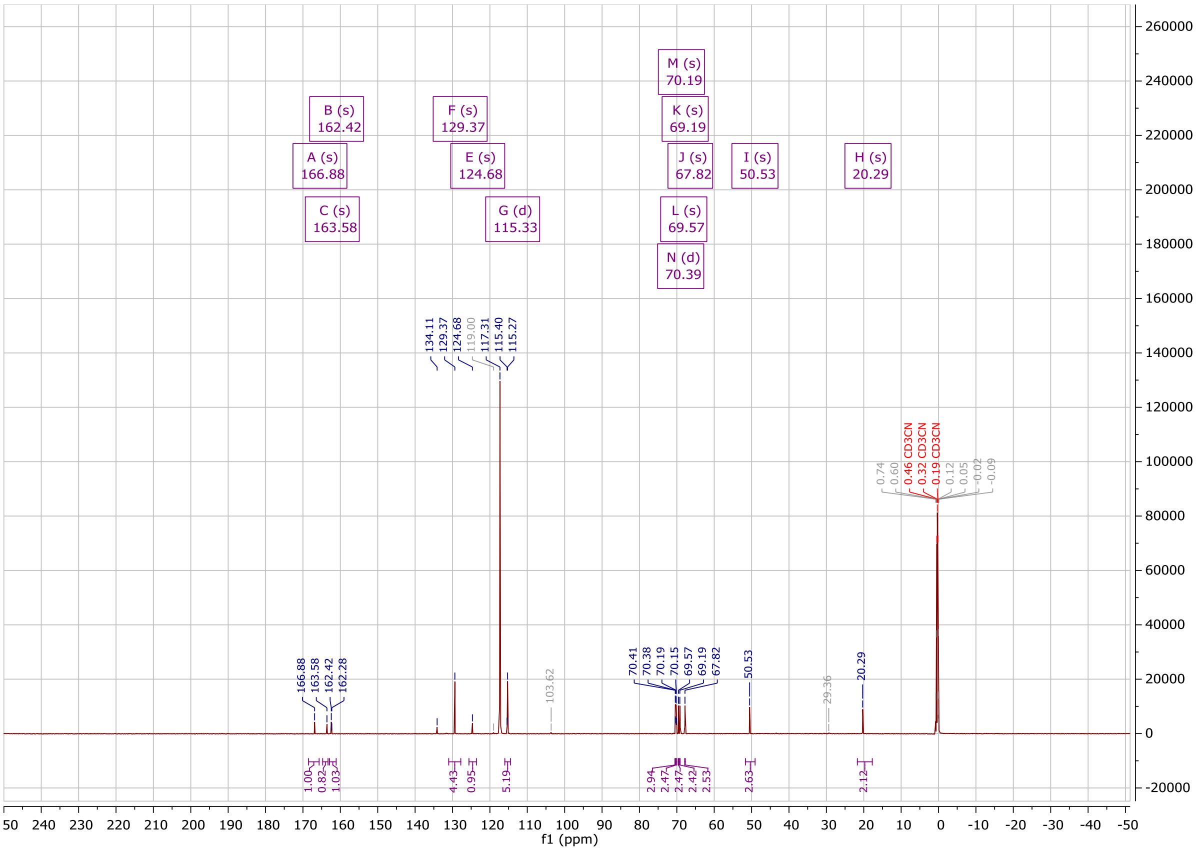

M (s)
70.19
B (s)
162.42
F (s)
129.37
K (s)
69.19
A (s)
166.88
E (s)
124.68
J (s)
67.82
I (s)
50.53
H (s)
20.29
C (s)
163.58
G (d)
115.33
L (s)
69.57
N (d)
70.39
0.46 CD3CN
0.32 CD3CN
0.19 CD3CN
f1 (ppm)

4.1.7 **3-(2-azidoethyl)-6-phenyl-1,2,4,5-tetrazine (Compound 8a):**

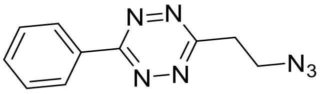

Compound **8a** was synthesized and characterized as described by Stieger *et al*.[8]

4.1.8 **Tetrazine-diethynyl-phosphinate**

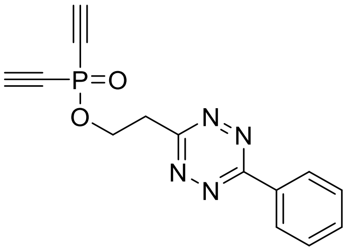

Synthesized according to Stieger *et al.*[9] from 50 mg 2-(6-phenyl-1,2,4,5-tetrazin-3-yl)ethan-1-ol. The desired product was obtained after column chromatography over silica, oxidation and lyophilization as a pink solid. (49 mg, 67%).

^{1}H NMR (600 MHz, DMSO-d_6) δ 8.61 – 8.45 (m, 2H), 7.78 – 7.65 (m, 3H), 4.87 (dd, *J* = 13.0, 2.5 Hz, 2H), 4.76 (dt, *J* = 9.7, 6.2 Hz, 2H), 3.79 (dd, *J* = 7.4, 5.0 Hz, 2H).
^{31}P NMR {^{1}H} (243 MHz, DMSO-d_6) δ -22.93.
^{13}C NMR (151 MHz, DMSO-d_6) – at the time point where the ^{13}C-NMR was recorded, already another species has formed (see **4.16**) - δ 166.98, 164.27, 158.32 (d, *J* = 54.6 Hz), 133.15, 132.11, 129.94, 128.08, 127.51, 125.38, 97.71 (d, *J* = 40.1 Hz), 95.40 (d, *J* = 45.6 Hz), 76.85 (d, *J* = 253.5 Hz), 66.35 (d, *J* = 6.2 Hz), 64.81 (d, *J* = 5.6 Hz), 35.60 (d, *J* = 8.4 Hz), 31.12.
UPLC-MS for $C_{14}H_{12}N_4O_2P^+$ [M+H]$^+$ calc.: 299.07; found: 299.10

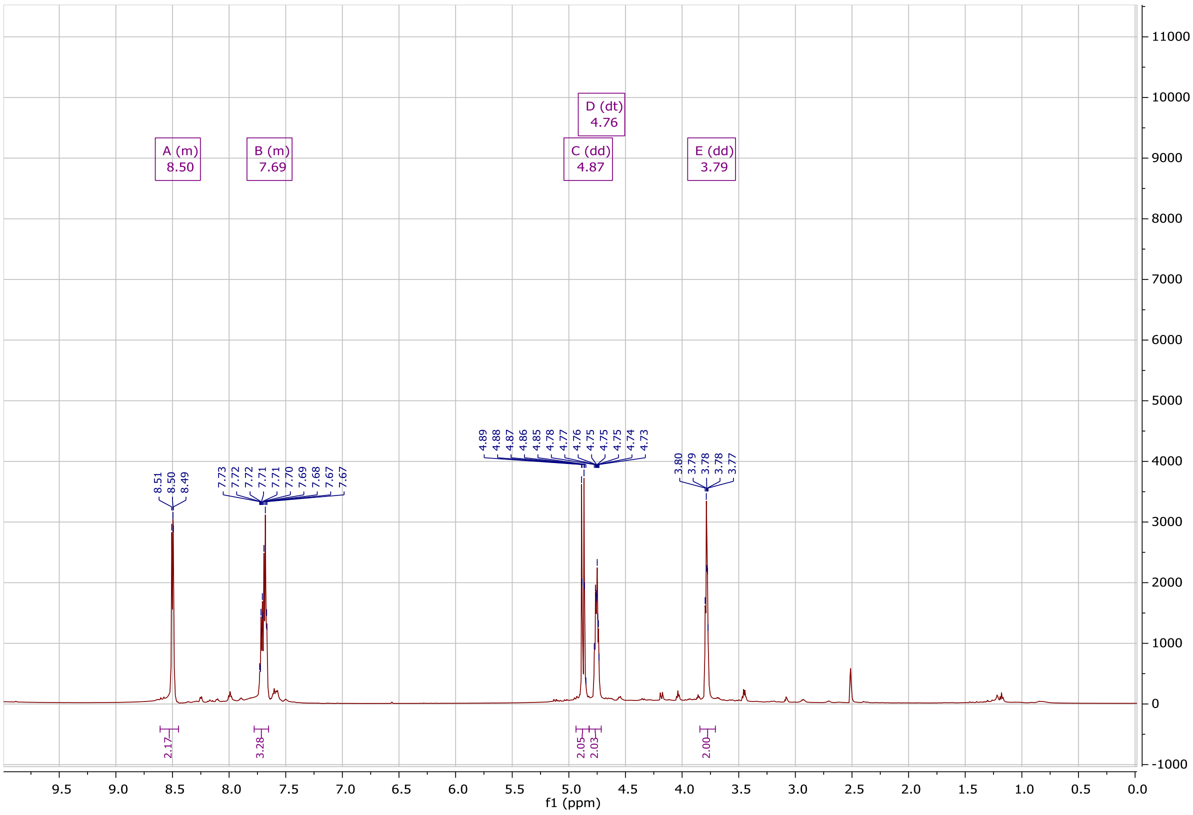

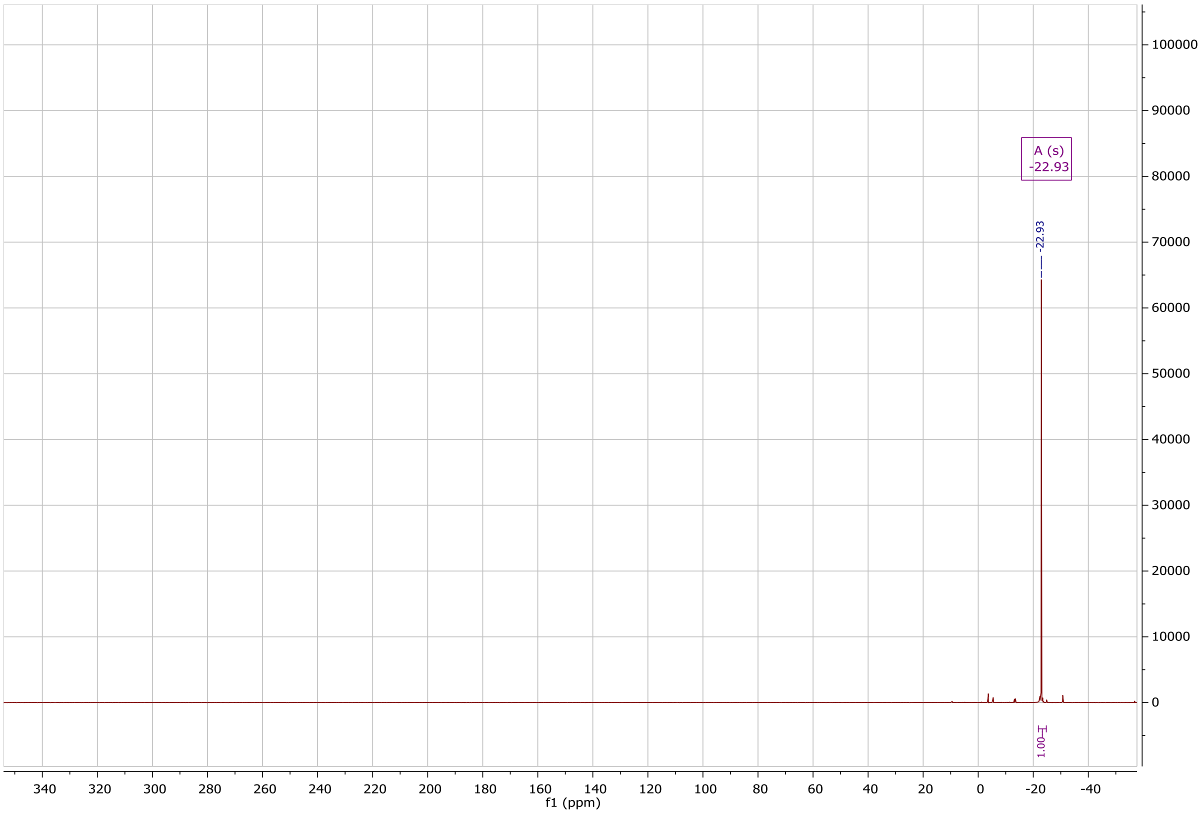
A (s)
-22.93
-22.93
1.00
f1 (ppm)

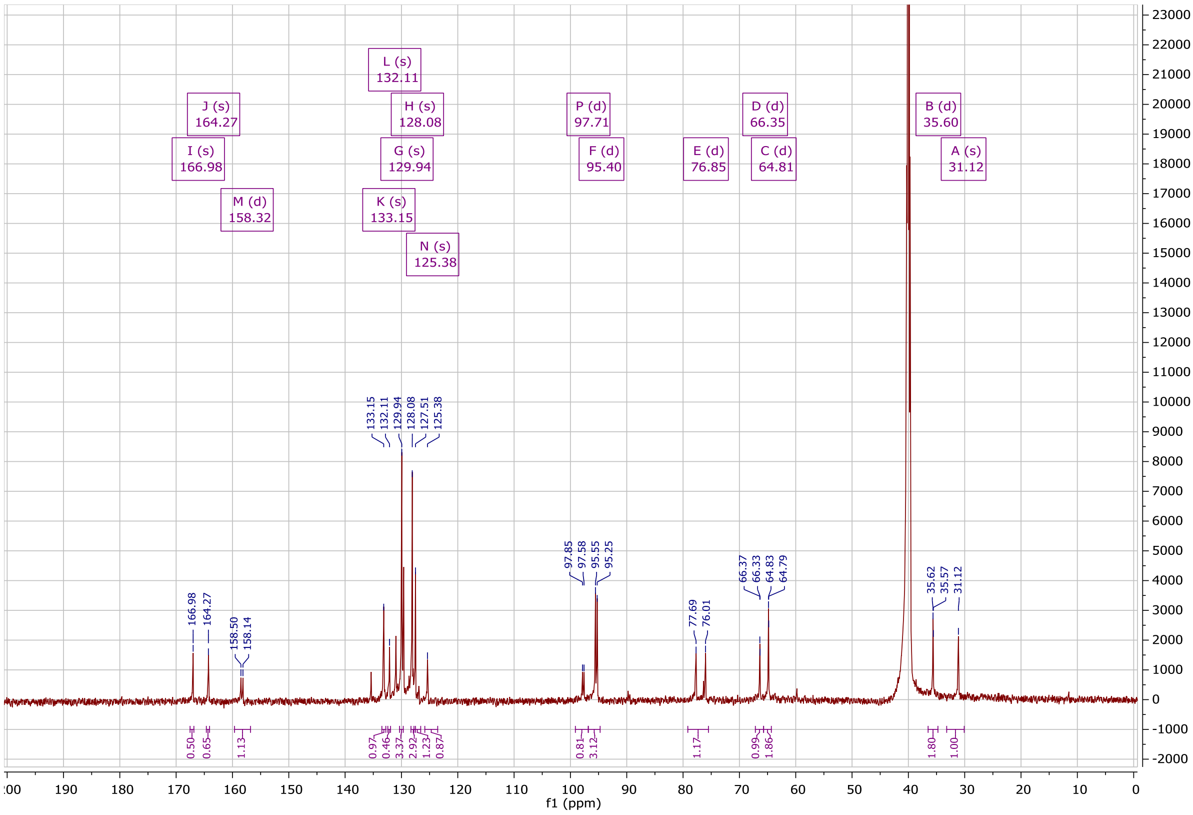
L (s)
132.11
J (s)
164.27
H (s)
128.08
P (d)
97.71
D (d)
66.35
B (d)
35.60
I (s)
166.98
G (s)
129.94
F (d)
95.40
E (d)
76.85
C (d)
64.81
A (s)
31.12
M (d)
158.32
K (s)
133.15
N (s)
125.38
166.98
164.27
158.50
158.14
133.15
132.11
129.94
128.08
127.51
125.38
97.85
97.58
95.55
95.25
77.69
76.01
66.37
66.33
64.83
64.79
35.62
35.57
31.12
0.50
0.65
1.13
0.97
0.46
3.37
2.92
1.23
0.87
0.81
3.12
1.17
0.99
1.86
1.80
1.00
f1 (ppm)

4.1.9 **5-ethynyl-3-phenyl-7,8-dihydro-[1,2]oxaphosphinino[4,3-c]pyridazine 5-oxide**

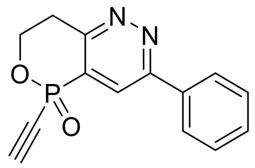

Compound **10** was obtained after prolonged storage of **9** in DMSO at room temperature. It was purified via semi preparative HPLC for analytical purposes.

^{1}H NMR (600 MHz, DMSO-d_6) δ 8.62 (d, J = 16.4 Hz, 1H), 8.27 (dd, J = 7.0, 2.7 Hz, 2H), 7.59 (dd, J = 5.2, 2.0 Hz, 3H), 4.96 (d, J = 11.9 Hz, 1H), 4.75 (dt, J = 13.3, 5.4 Hz, 2H), 3.69 – 3.38 (m, 2H).
^{31}P NMR {^{1}H} (243 MHz, DMSO-d_6) δ -3.71.
^{13}C NMR (151 MHz, DMSO-d_6) δ 158.53 (d, J = 4.4 Hz), 158.17 (d, J = 5.3 Hz), 135.40, 131.01, 129.62, 128.21 (d, J = 140.9 Hz), 127.53, 125.36 (d, J = 8.0 Hz), 97.73 (d, J = 40.0 Hz), 77.12 (d, J = 224.5 Hz), 66.34 (d, J = 6.1 Hz), 31.11.
UPLC-MS for $C_{14}H_{12}N_2O_2P^{+}$ [M+H]$^{+}$ calc.: 271.06; found: 271.11

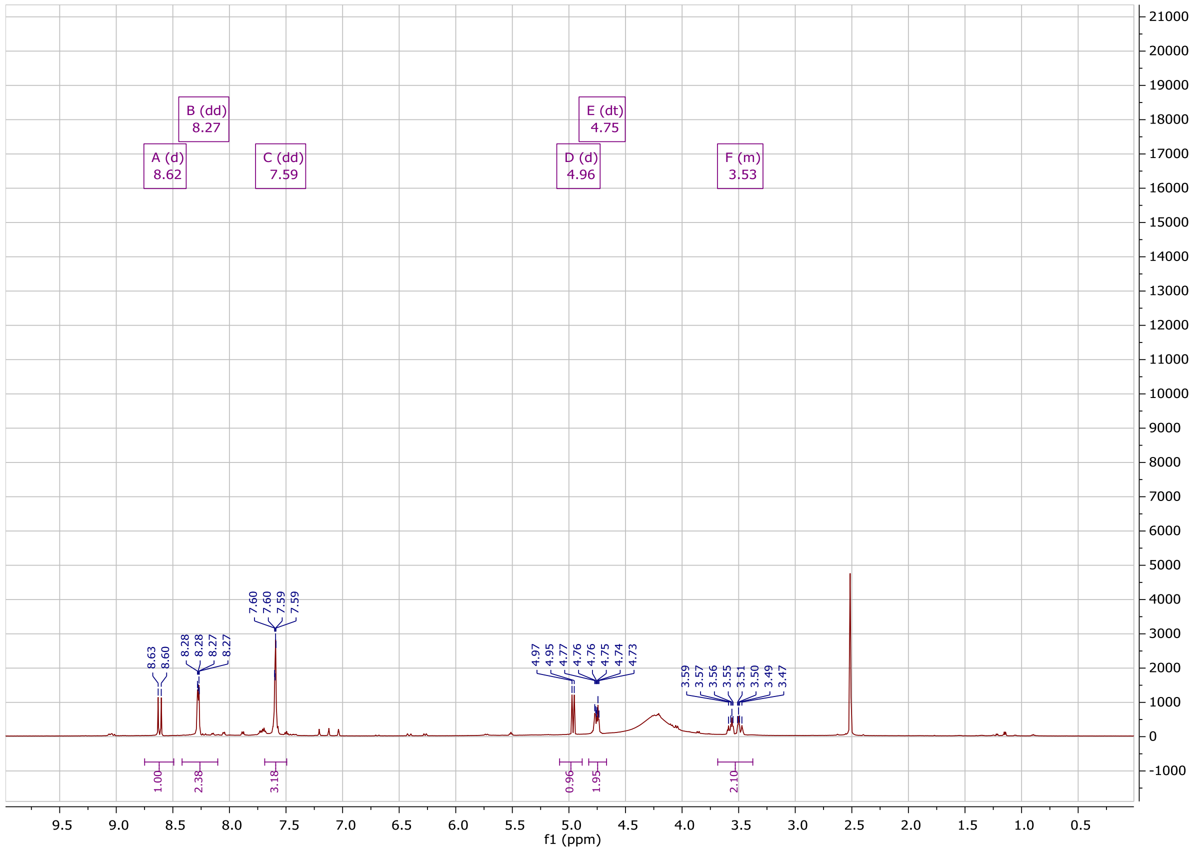

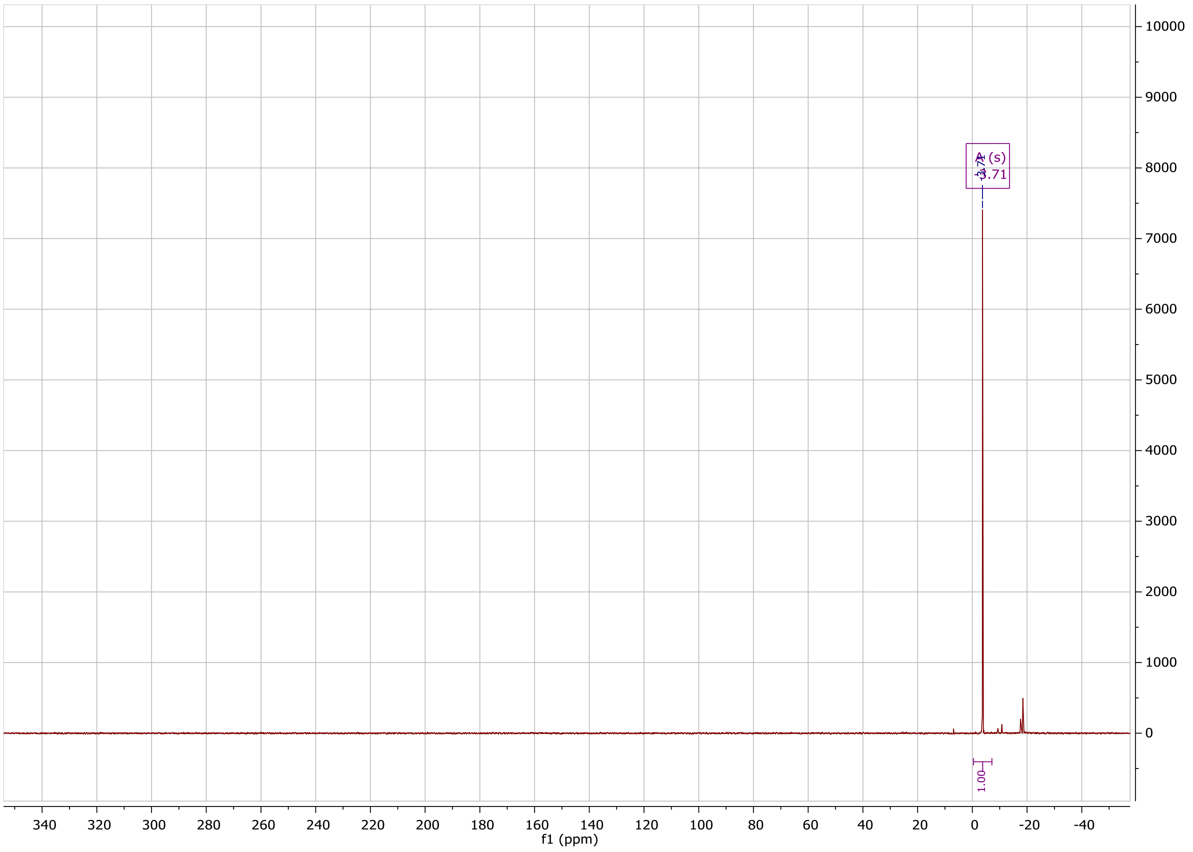
A (s)
-3.71
1.00
f1 (ppm)

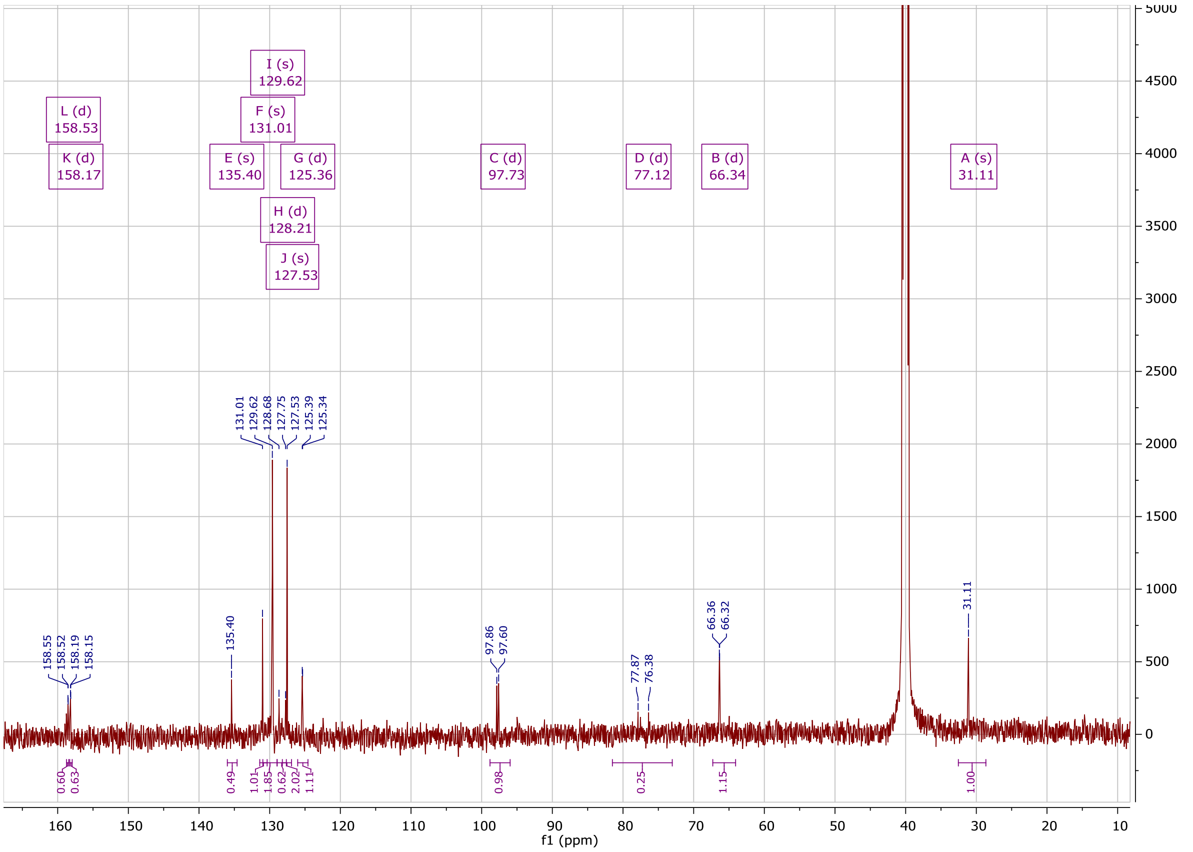
I (s)
129.62
L (d)
158.53
F (s)
131.01
K (d)
158.17
E (s)
135.40
G (d)
125.36
C (d)
97.73
D (d)
77.12
B (d)
66.34
A (s)
31.11
H (d)
128.21
J (s)
127.53
158.55
158.52
158.19
158.15
135.40
131.01
129.62
128.68
127.75
127.53
125.39
125.34
97.86
97.60
77.87
76.38
66.36
66.32
31.11
0.60
0.63
0.49
1.01
1.85
0.62
2.02
1.11
0.98
0.25
1.15
1.00
f1 (ppm)

4.1.10 **4(/5)-((5-azidopentyl)carbamoyl)-2-(6-hydroxy-3-oxo-3H-xanthen-9-yl)benzoic acid**

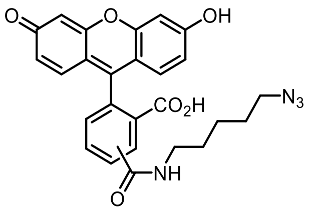

To fluorescein carboxylic acid (mix of 5 and 6 isomers, 165 mg, 0.43 mmol, 1.0 equiv.) in DMF (8 mL, 0.05 M) was added EDC (166 mg, 0.87 mmol, 2.0 equiv.), HOBt (117 mg, 0.87 mmol, 2 equiv.), 5-azidopentamine (56 mg, 0.43 mmol, 1.0 equiv.) and Et_3N (121 µL, 0.87 mmol, 2.0 equiv.). The reaction mixture was left stirring overnight. The reaction mixture was then freeze dried and purified by preparatory HPLC (30-95% MeCN/Water) to give the product as a mixture of 2 regioisomers (50 mg, 0.24 mmol, 24%). HRMS (ESI) for $C_{26}H_{23}N_4O_6^+$ [M+H$^+$] calc.: 487.1613, found 487.1608.

UPLC UV trace (gradient D, 220 nm):

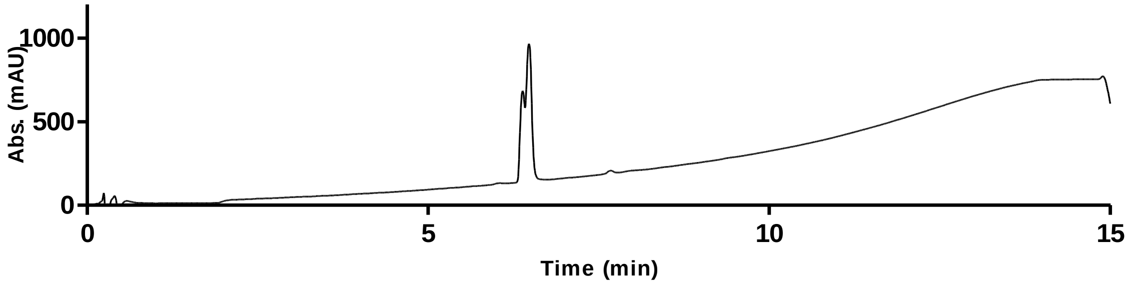

4.2 Synthesis of DTPOs

4.2.1 Initial synthetic route towards DTPOs:

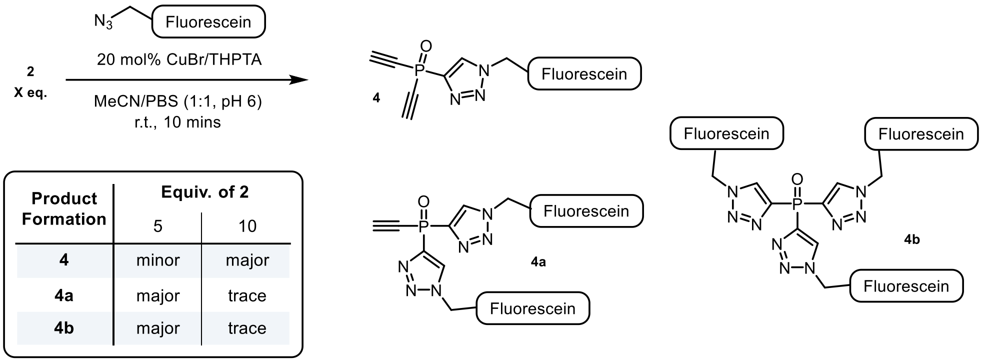

Product Formation	Equiv. of 2: 5	Equiv. of 2: 10
**4**	minor	major
**4a**	major	trace
**4b**	major	trace

Figure S38: Observed product formation for the initial and the adjusted reaction conditions towards DTPOs.

4.2.2 General Procedure A for the synthesis of DTPOs:

The azide was dissolved in MeCN/PBS (1:1, 50 mM, pH 6). 10 eq. triethynyl-phosphine oxide and 20 mol% CuBr (preformed THPTA-complex) were added and the mixture was vigorously stirred at room temperature. After full consumption of the starting material, the reaction was terminated by the addition of water/MeCN containing 0.1 % TFA and purified via semi-preparative HPLC.

4.2.3 Fluorescein-DTPO (DTPO 4):

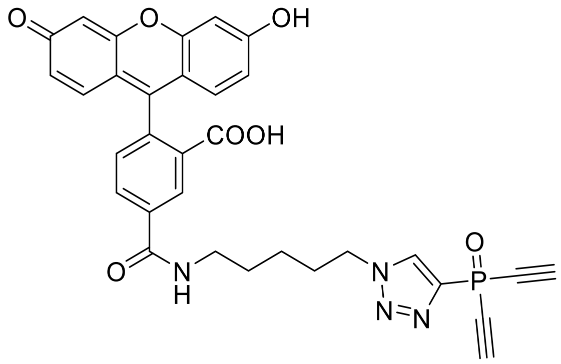

Was synthesized according to **general procedure A** from 15 mg FAM-N_3. (9.5 mg, 51% yield)

^{1}H NMR: (600 MHz, DMSO-d_6) δ 8.89 (s, 1H), 8.81 (t, J = 5.7 Hz, 1H), 8.46 (d, J = 1.5 Hz, 1H), 8.24 (dd, J = 8.0, 1.6 Hz, 1H), 7.37 (d, J = 8.0 Hz, 1H), 6.70 (d, J = 2.3 Hz, 2H), 6.59 (d, J = 8.7 Hz, 2H), 6.56 (dd, J = 8.7, 2.3 Hz, 2H), 5.01 (d, J = 11.6 Hz, 2H), 4.50 (t, J = 7.2 Hz, 2H), 3.40 – 3.26 (m, 2H), 2.00 – 1.87 (m, 2H), 1.61 (p, J = 7.2 Hz, 3H), 1.42 – 1.28 (m, 2H).
^{31}P NMR {^{1}H}: (243 MHz, DMSO-d_6) δ -37.44.
^{13}C NMR: (151 MHz, DMSO-d_6) δ 168.66, 164.99, 160.09, 158.67 (d, J = 36.1 Hz), 155.01, 152.30, 136.83, 135.11, 131.68 (d, J = 35.8 Hz), 129.61, 126.93, 124.67, 123.67, 113.14, 109.59, 102.74, 97.48 (d, J = 37.5 Hz), 78.40 (d, J = 203.1 Hz), 50.29, 40.53, 29.67, 28.78, 23.77.
HRMS for $C_{32}H_{26}N_4O_7P^+$ $[M+H]^+$ calc.: 609.1534 found: 609.1494.

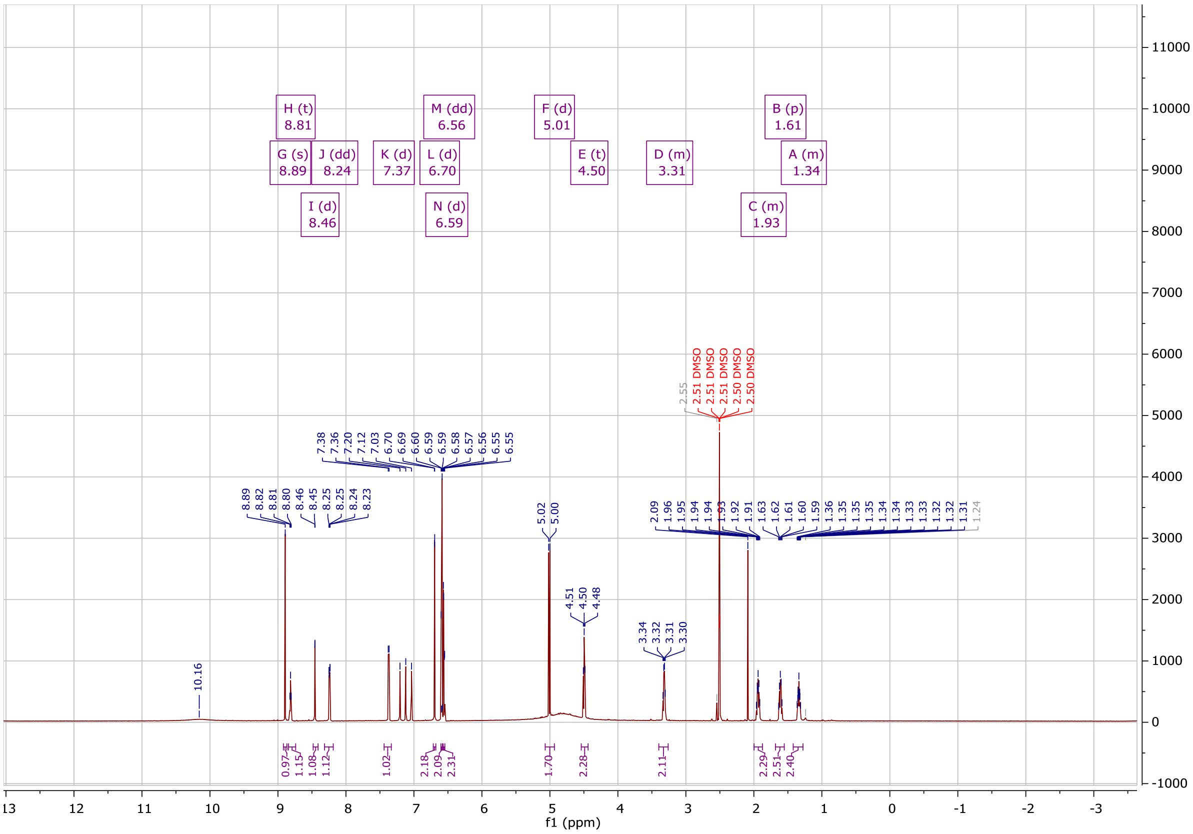

H (t) 8.81
G (s) 8.89
J (dd) 8.24
I (d) 8.46
K (d) 7.37
M (dd) 6.56
L (d) 6.70
N (d) 6.59
F (d) 5.01
E (t) 4.50
D (m) 3.31
C (m) 1.93
B (p) 1.61
A (m) 1.34
2.51 DMSO
2.51 DMSO
2.51 DMSO
2.50 DMSO
2.50 DMSO
f1 (ppm)

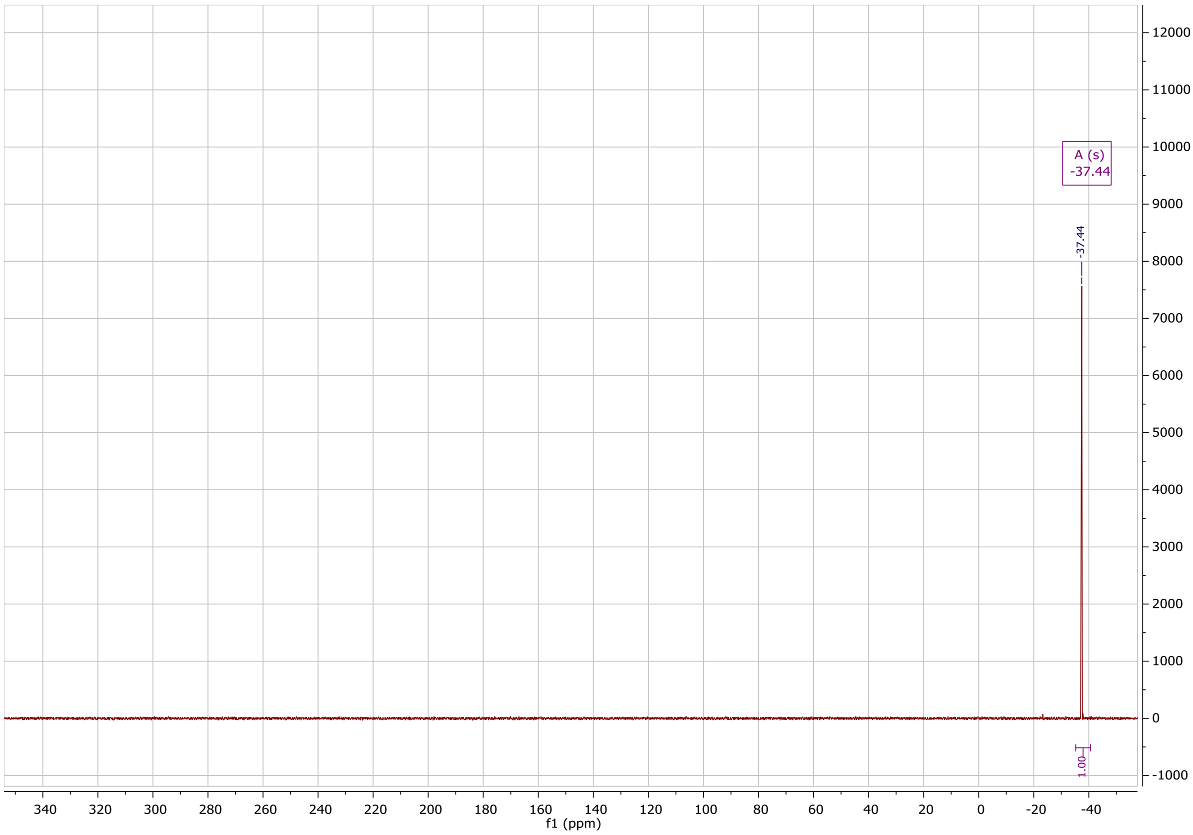

A (s) -37.44
-37.44
1.00
f1 (ppm)

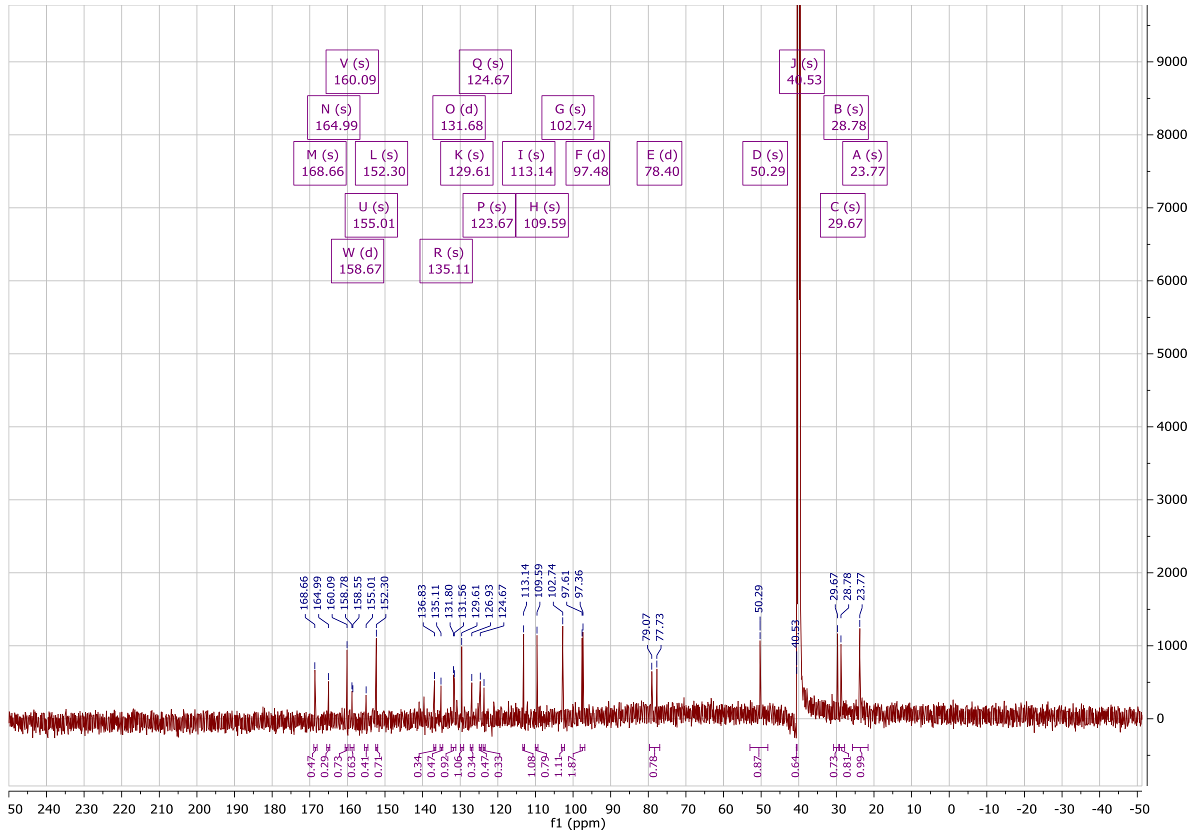

V (s) 160.09
Q (s) 124.67
J (s) 40.53
N (s) 164.99
O (d) 131.68
G (s) 102.74
B (s) 28.78
M (s) 168.66
L (s) 152.30
K (s) 129.61
I (s) 113.14
F (d) 97.48
E (d) 78.40
D (s) 50.29
A (s) 23.77
U (s) 155.01
P (s) 123.67
H (s) 109.59
C (s) 29.67
W (d) 158.67
R (s) 135.11
168.66
164.99
160.09
158.78
158.55
155.01
152.30
136.83
135.11
131.80
131.56
129.61
126.93
124.67
113.14
109.59
102.74
97.61
97.36
79.07
77.73
50.29
40.53
29.67
28.78
23.77
0.47
0.29
0.73
0.63
0.41
0.71
0.34
0.47
0.92
1.06
0.34
0.47
0.33
1.08
0.79
1.11
1.87
0.78
0.87
0.64
0.73
0.81
0.99
f1 (ppm)

4.2.4 **Coumarin-PEG$_4$-N$_3$ (Compound 5a)**

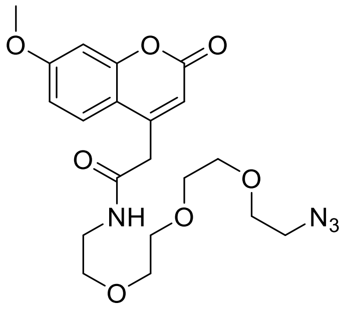

15 mg of 7-Methoxycoumarin-4-acetic acid and 24 mg HATU (0.98 eq.) were dissolved in 500 µl DMF followed by the addition of 30 µl DIPEA. After 5 minutes, 14 mg of 2-(2-(2-(2-azidoethoxy)ethoxy)ethoxy)ethan-1-amine were added and the reaction was allowed to proceed for 2 h at room temperature. The product was purified via semi preparative HPLC and obtained in 72 % yield.

^{1}H NMR (600 MHz, DMSO-d_6) δ 8.29 – 8.18 (m, 1H), 7.66 (dd, J = 8.8, 3.2 Hz, 1H), 6.84 (dt, J = 8.8, 3.0 Hz, 1H), 6.81 (t, J = 2.9 Hz, 1H), 6.19 (d, J = 3.1 Hz, 1H), 3.83 (d, J = 3.2 Hz, 3H), 3.63 (d, J = 2.9 Hz, 2H), 3.62 – 3.58 (m, 2H), 3.58 – 3.50 (m, 8H), 3.46 (d, J = 2.6 Hz, 2H), 3.33 (td, J = 5.0, 3.2 Hz, 2H), 3.27 (qd, J = 5.6, 2.9 Hz, 2H).
^{13}C NMR (151 MHz, DMSO-d_6) δ 168.08, 162.69, 160.55, 155.41, 150.97, 126.66, 113.07, 112.99, 112.21, 100.95, 70.38 (3C), 70.15, 69.82, 69.52, 55.99, 50.55, 39.44, 39.42.
HRMS for $C_{20}H_{27}N_4O_7^+$ [M+H]$^+$ calc.: 435.1874; found: 435.1972.

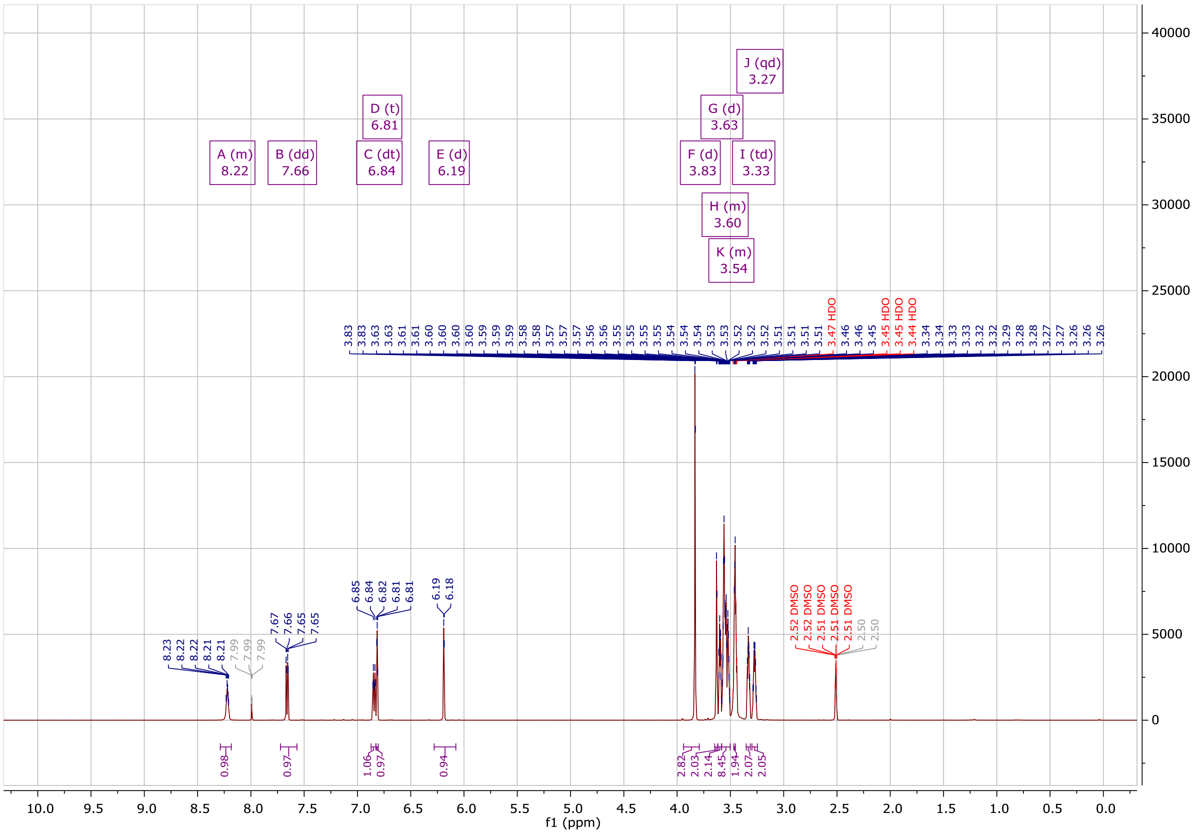

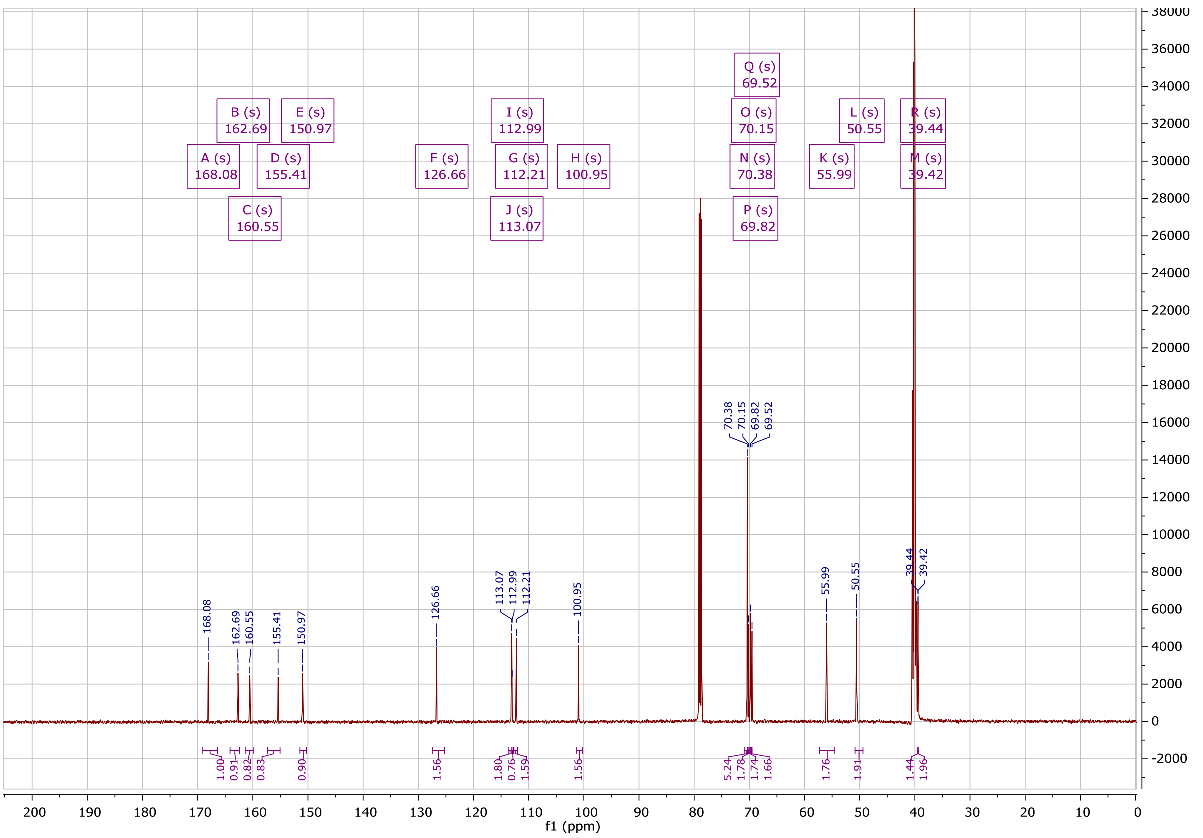

4.2.5 **Coumarin-PEG$_4$-DTPO (DTPO 5)**

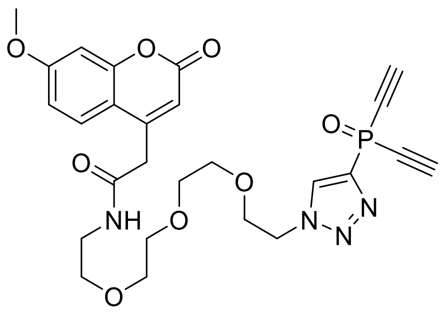

Was synthesized according to **general procedure A** from 4 mg **5a**. (2.5 mg, 49 %)

^{1}H NMR (600 MHz, DMSO-d_6) δ 8.79 (d, J = 2.0 Hz, 1H), 8.32 (t, J = 5.6 Hz, 1H), 7.69 (d, J = 8.9 Hz, 1H), 7.00 (d, J = 2.5 Hz, 1H), 6.96 (dd, J = 8.8, 2.5 Hz, 1H), 6.25 (s, 1H), 5.03 (d, J = 11.6 Hz, 2H), 4.65 (t, J = 5.1 Hz, 2H), 3.89 – 3.86 (m, 2H), 3.86 (s, 3H), 3.69 (s, 2H), 3.56 – 3.51 (m, 2H), 3.49 – 3.45 (m, 8H), 3.28 – 3.18 (m, 2H).
^{31}P NMR {^{1}H} (243 MHz, DMSO-d_6) δ -37.33.
^{13}C NMR (151 MHz, DMSO-d_6) δ 168.20, 162.82, 160.60, 155.39, 151.62, 140.08 (d, J = 190.3 Hz), 132.15 (d, J = 35.1 Hz), 126.98, 113.03 (2C), 112.59, 101.30, 97.56 (d, J = 37.4 Hz), 78.33 (d, J = 203.7 Hz), 70.14, 70.03 (2C), 69.94, 69.38, 68.65, 56.41, 50.25, 39.34, 39.22.
HRMS for $C_{26}H_{30}N_4O_8P^+$ $[M+H]^+$ calc.: 557.1796 Da; found: 557.1794 Da.

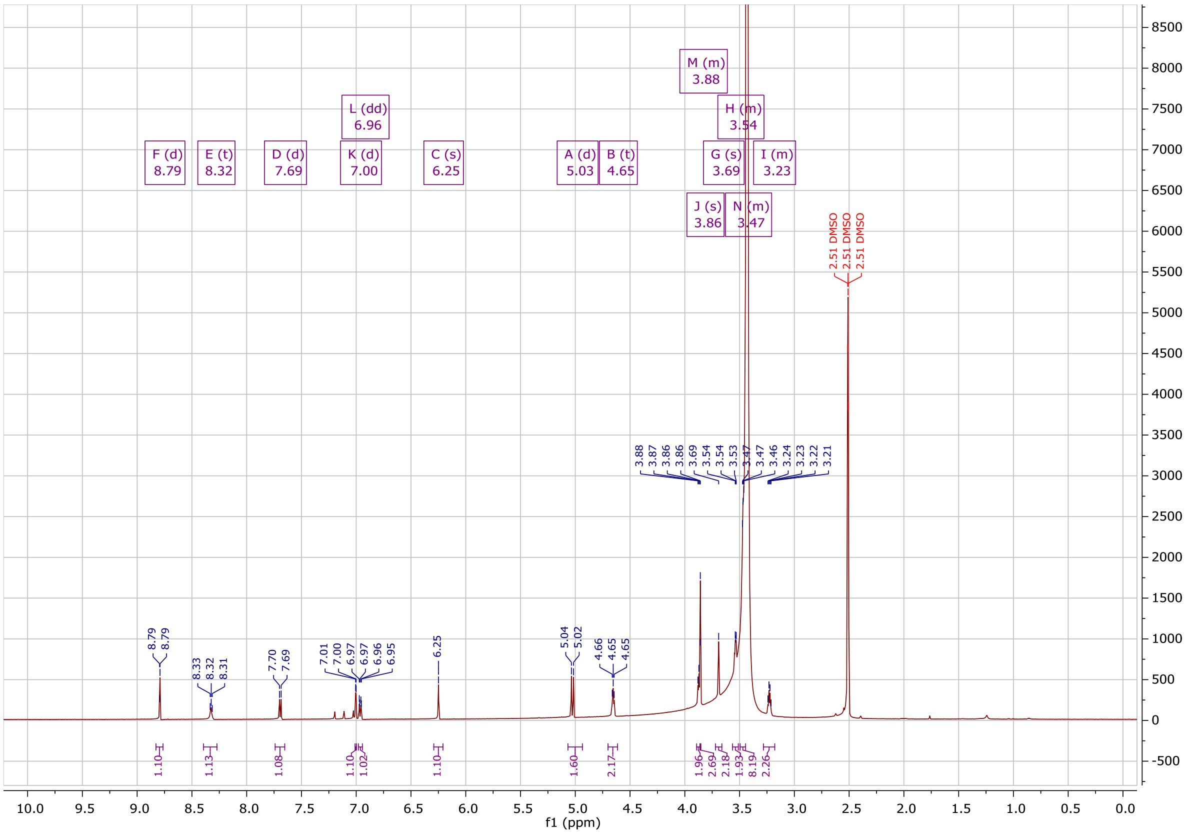

F (d) 8.79
E (t) 8.32
D (d) 7.69
L (dd) 6.96
K (d) 7.00
C (s) 6.25
A (d) 5.03
B (t) 4.65
M (m) 3.88
H (m) 3.54
G (s) 3.69
I (m) 3.23
J (s) 3.86
N (m) 3.47
2.51 DMSO
f1 (ppm)

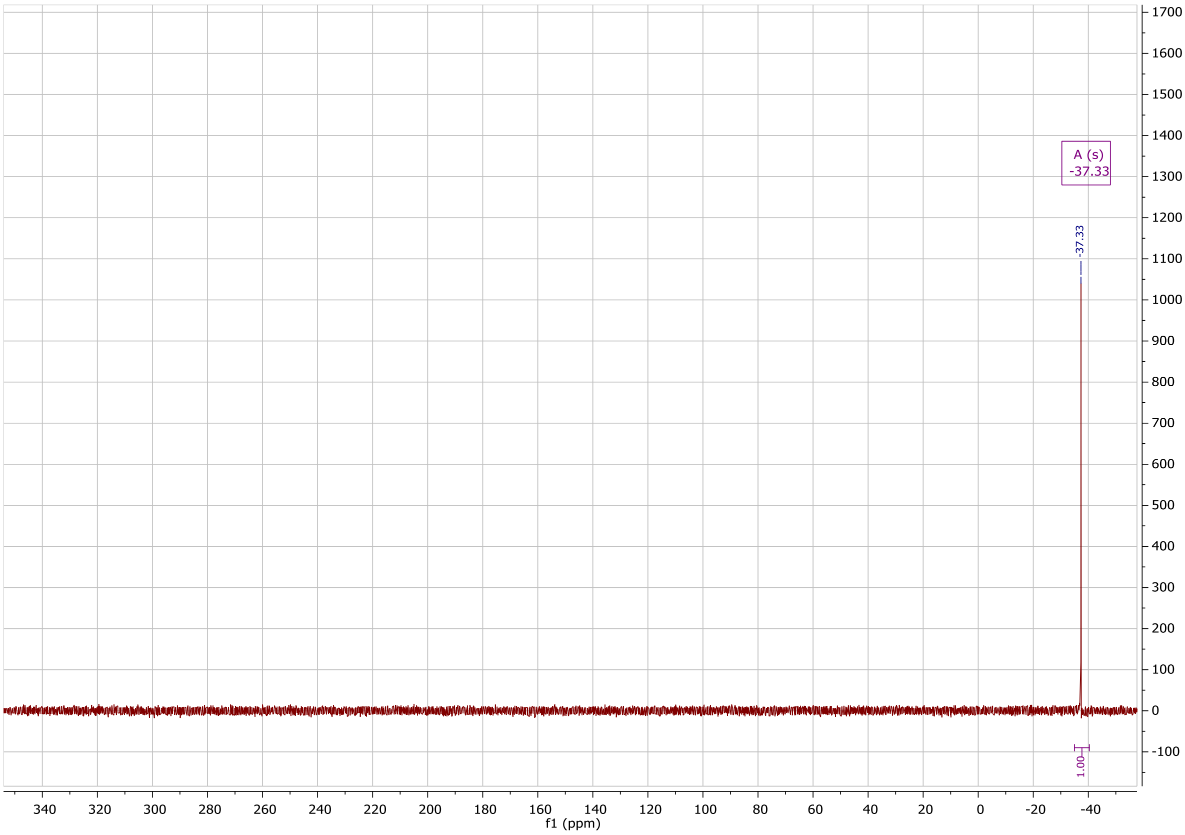

A (s) -37.33
f1 (ppm)

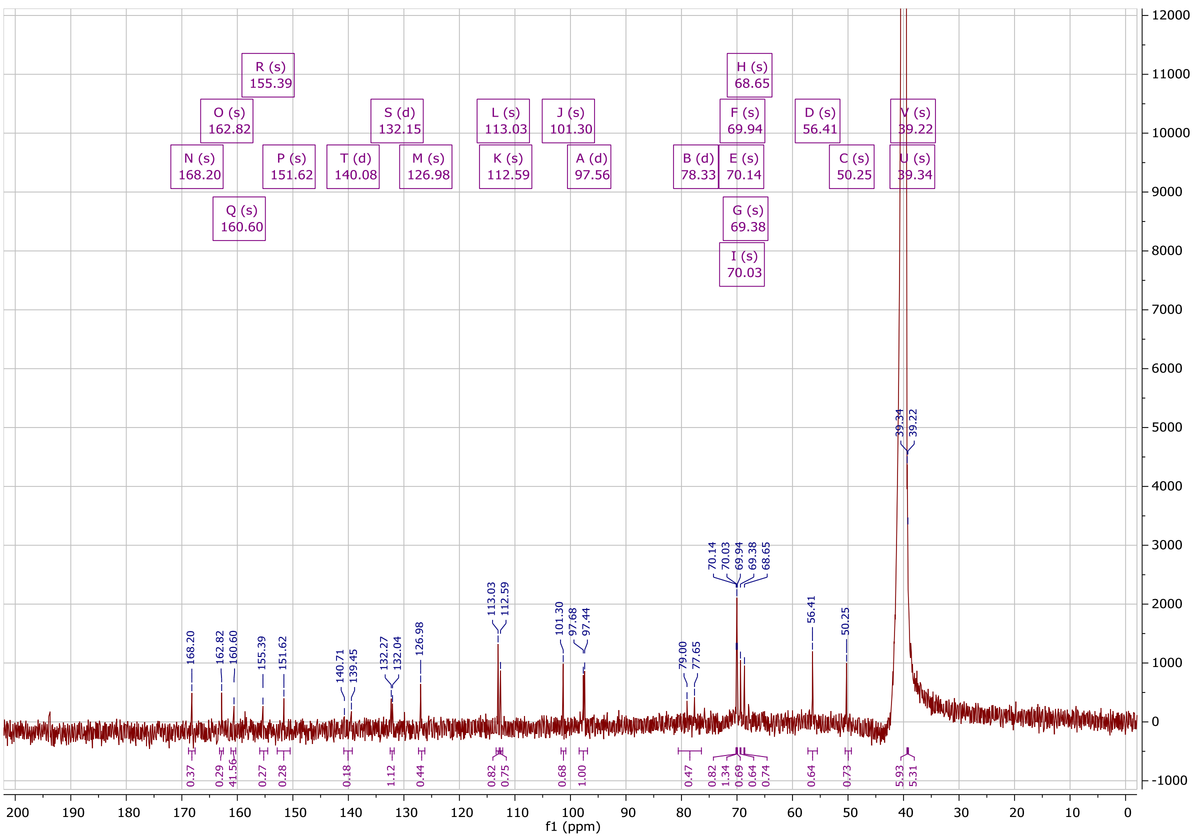

R (s) 155.39
O (s) 162.82
N (s) 168.20
P (s) 151.62
Q (s) 160.60
T (d) 140.08
S (d) 132.15
M (s) 126.98
L (s) 113.03
K (s) 112.59
J (s) 101.30
A (d) 97.56
B (d) 78.33
H (s) 68.65
F (s) 69.94
E (s) 70.14
G (s) 69.38
I (s) 70.03
D (s) 56.41
C (s) 50.25
V (s) 39.22
U (s) 39.34
f1 (ppm)

4.2.6 **Sulfo-Rhodamine-B-DTPO (DTPO 6):**

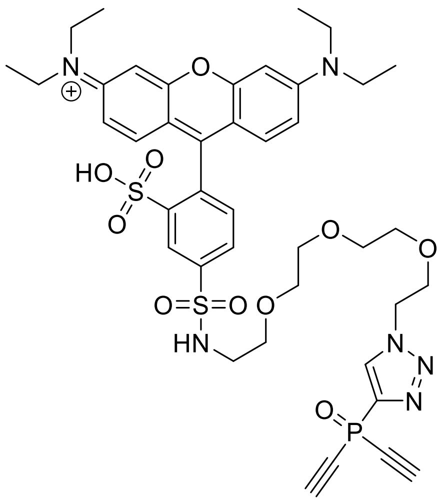

Was synthesized according to **general procedure A** from 8 mg **6a**. (5.66 mg, 61 % yield)

^{1}H NMR (600 MHz, DMSO-*d*6) δ 8.78 (s, 1H), 8.43 (d, *J* = 2.0 Hz, 1H), 8.03 (t, *J* = 6.0 Hz, 1H), 7.95 (dd, *J* = 7.9, 2.0 Hz, 1H), 7.47 (d, *J* = 7.9 Hz, 1H), 7.23 (s, 2H), 7.14 (s, 2H), 7.08 – 7.02 (m, 3H), 6.99 (s, 1H), 6.98 (s, 1H), 6.94 (d, *J* = 2.4 Hz, 2H), 5.01 (d, *J* = 11.6 Hz, 2H), 4.65 (t, *J* = 5.2 Hz, 2H), 3.87 (t, *J* = 5.2 Hz, 2H), 3.69 – 3.61 (m, 8H), 3.54 (dd, *J* = 5.9, 3.4 Hz, 2H), 3.50 – 3.43 (m, 8H), 3.03 (q, *J* = 5.8 Hz, 2H), 1.21 (t, *J* = 7.1 Hz, 12H).
^{31}P NMR {^{1}H} (243 MHz, DMSO-*d*6) δ -37.42.
^{13}C NMR (151 MHz, DMSO-*d*6) δ 158.68, 158.46, 157.96, 157.58, 155.49, 148.40, 142.10, 133.45, 133.11, 132.25, 132.01, 131.07, 127.03, 126.14, 114.10, 113.94, 97.52 (d, *J* = 37.5 Hz), 95.86, 78.37 (d, *J* = 203.1 Hz), 70.13, 70.12, 70.05, 69.94, 69.57, 68.66, 50.25, 45.72, 42.93, 12.93.
HRMS for $C_{41}H_{50}N_6O_{10}PS_2^+$ $[M]^+$ calc.: 881.2762 Da; found: 881.2770 Da.

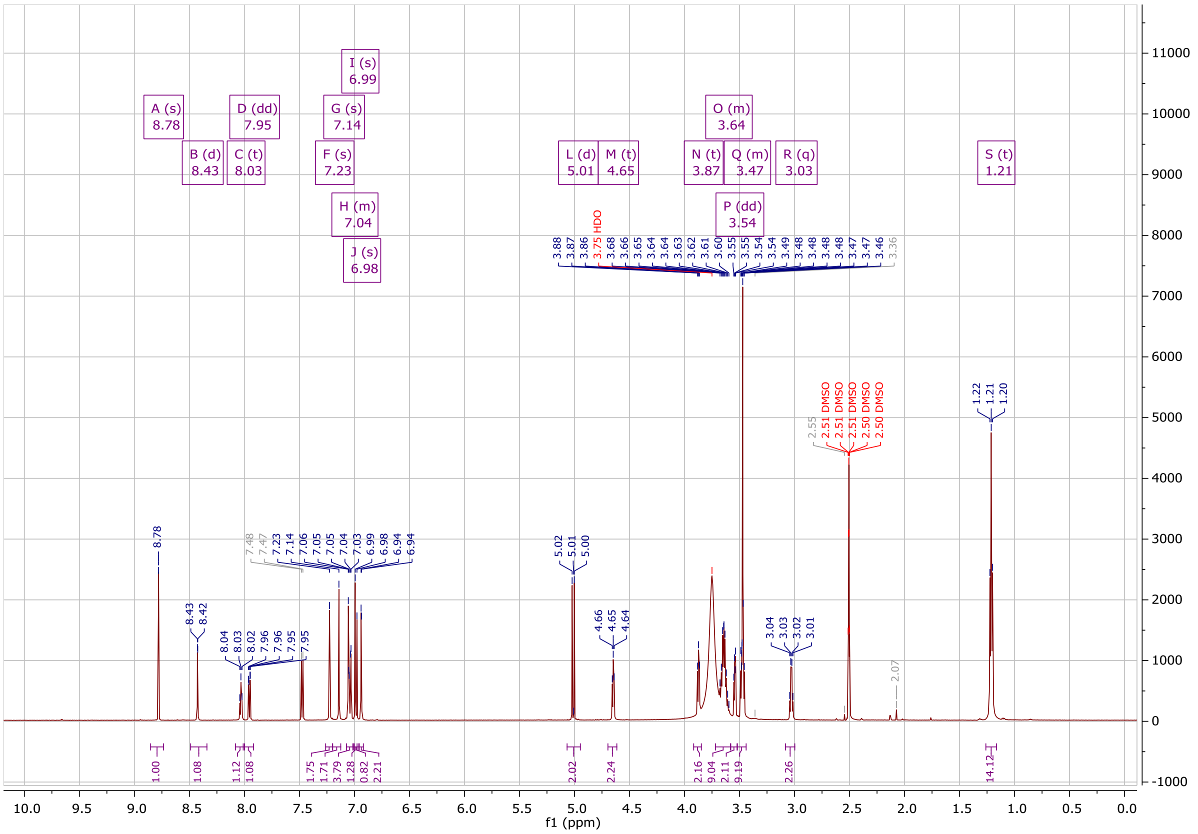

A (s) 8.78
B (d) 8.43
C (t) 8.03
D (dd) 7.95
F (s) 7.23
G (s) 7.14
H (m) 7.04
I (s) 6.99
J (s) 6.98
L (d) 5.01
M (t) 4.65
N (t) 3.87
O (m) 3.64
P (dd) 3.54
Q (m) 3.47
R (q) 3.03
S (t) 1.21
3.75 HDO
2.51 DMSO
2.50 DMSO
f1 (ppm)

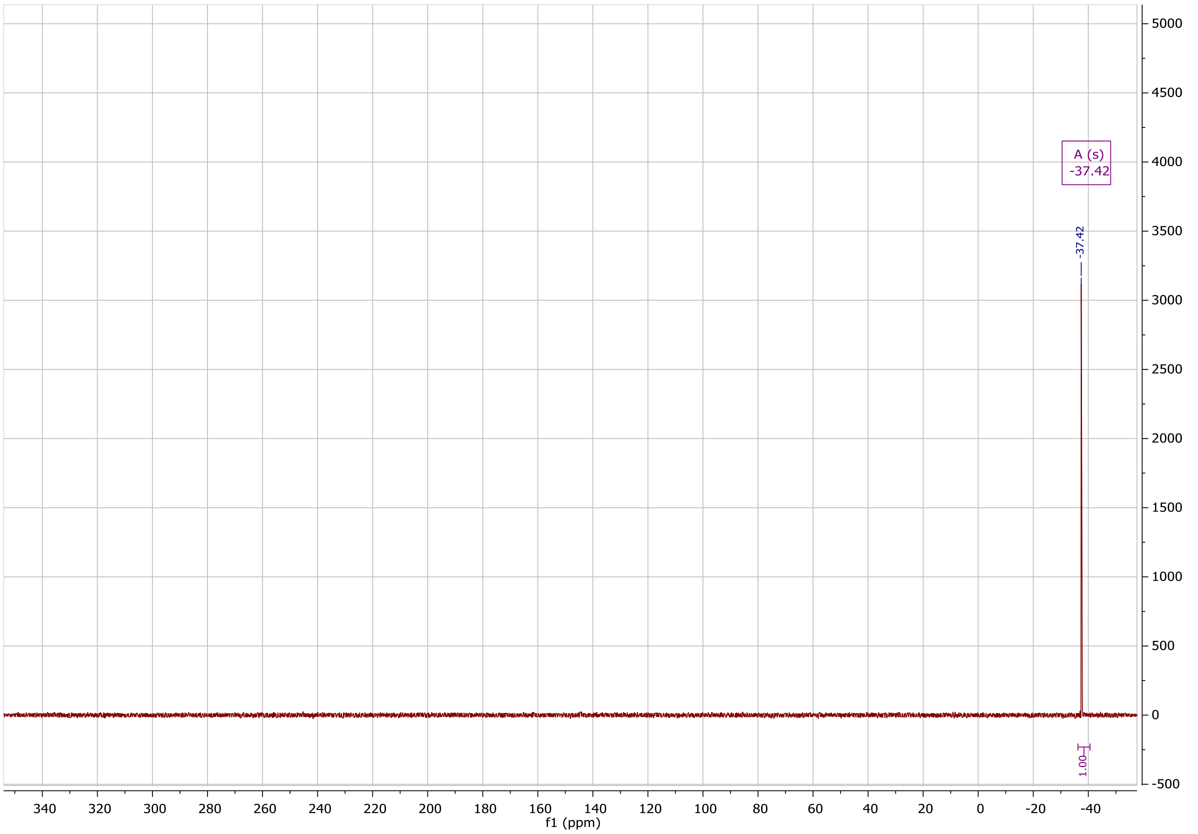

A (s) -37.42
-37.42
1.00
f1 (ppm)

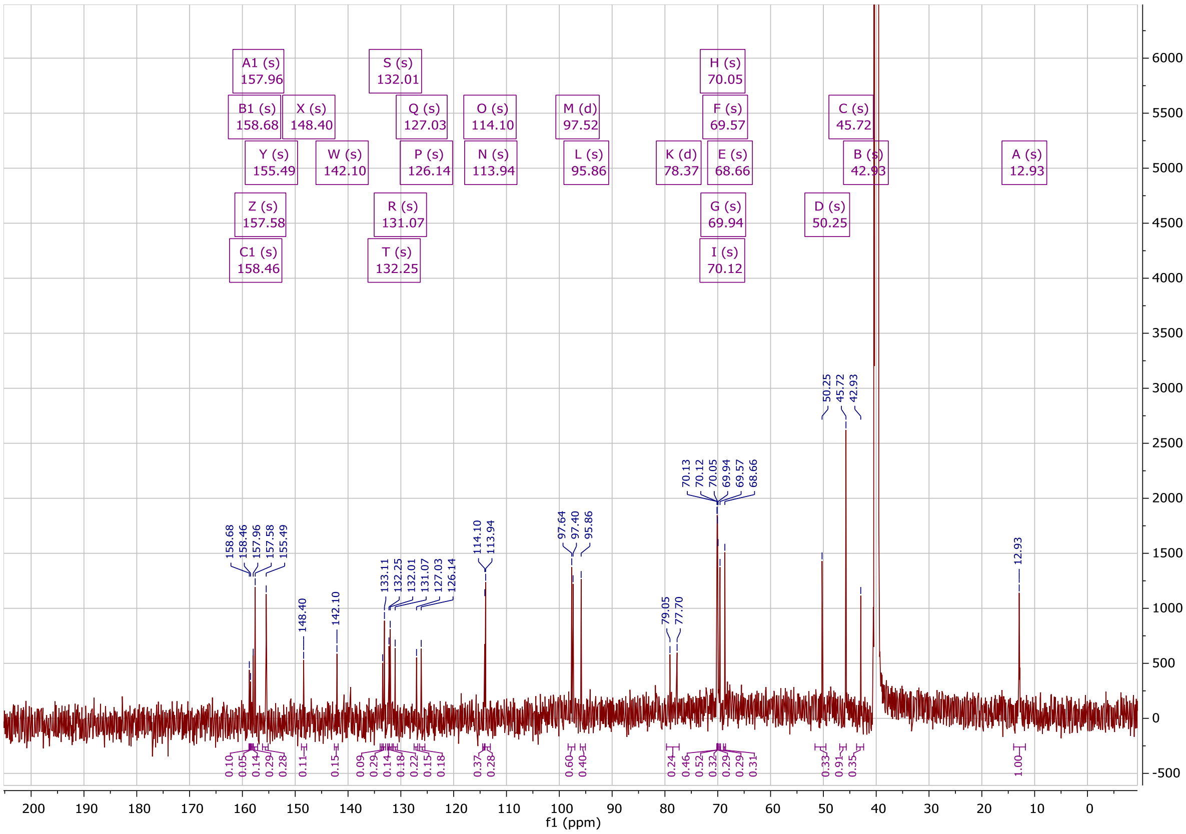

A1 (s) 157.96
S (s) 132.01
H (s) 70.05
B1 (s) 158.68
X (s) 148.40
Q (s) 127.03
O (s) 114.10
M (d) 97.52
F (s) 69.57
C (s) 45.72
Y (s) 155.49
W (s) 142.10
P (s) 126.14
N (s) 113.94
L (s) 95.86
K (d) 78.37
E (s) 68.66
B (s) 42.93
A (s) 12.93
Z (s) 157.58
R (s) 131.07
G (s) 69.94
D (s) 50.25
C1 (s) 158.46
T (s) 132.25
I (s) 70.12
f1 (ppm)

4.2.7 **Tetrazine-PEG$_3$-DTPO (DTPO 7):**

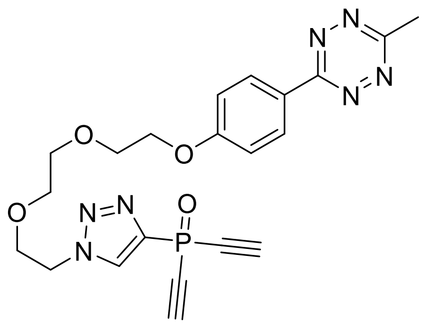

Was synthesized according to **general procedure A** from 17 mg **7b**. (13.07 mg, 56% yield).

^{1}H NMR (600 MHz, DMSO-d_6) δ 8.81 (s, 1H), 8.45 – 8.38 (m, 2H), 7.23 – 7.15 (m, 2H), 5.02 (d, J = 11.6 Hz, 2H), 4.67 (t, J = 5.2 Hz, 2H), 4.23 – 4.19 (m, 2H), 3.90 (t, J = 5.2 Hz, 2H), 3.80 – 3.72 (m, 2H), 3.59 (q, J = 1.5 Hz, 4H), 2.97 (s, 3H).
^{31}P NMR {^{1}H} (243 MHz, DMSO-d_6) δ -37.38.
^{13}C NMR (151 MHz, DMSO-d_6) δ 166.97, 163.42, 162.42, 140.15 (d, J = 190.3 Hz), 132.17 (d, J = 36.0 Hz), 129.66, 124.54, 115.86, 97.54 (d, J = 37.5 Hz), 78.38 (d, J = 203.2 Hz), 70.22, 70.00, 69.22, 68.70, 67.95, 50.27, 21.18.
HRMS for $C_{21}H_{23}N_7O_4P^+$ $[M+H]^+$ calc.: 468.1544 Da; found: 468.1564 Da.

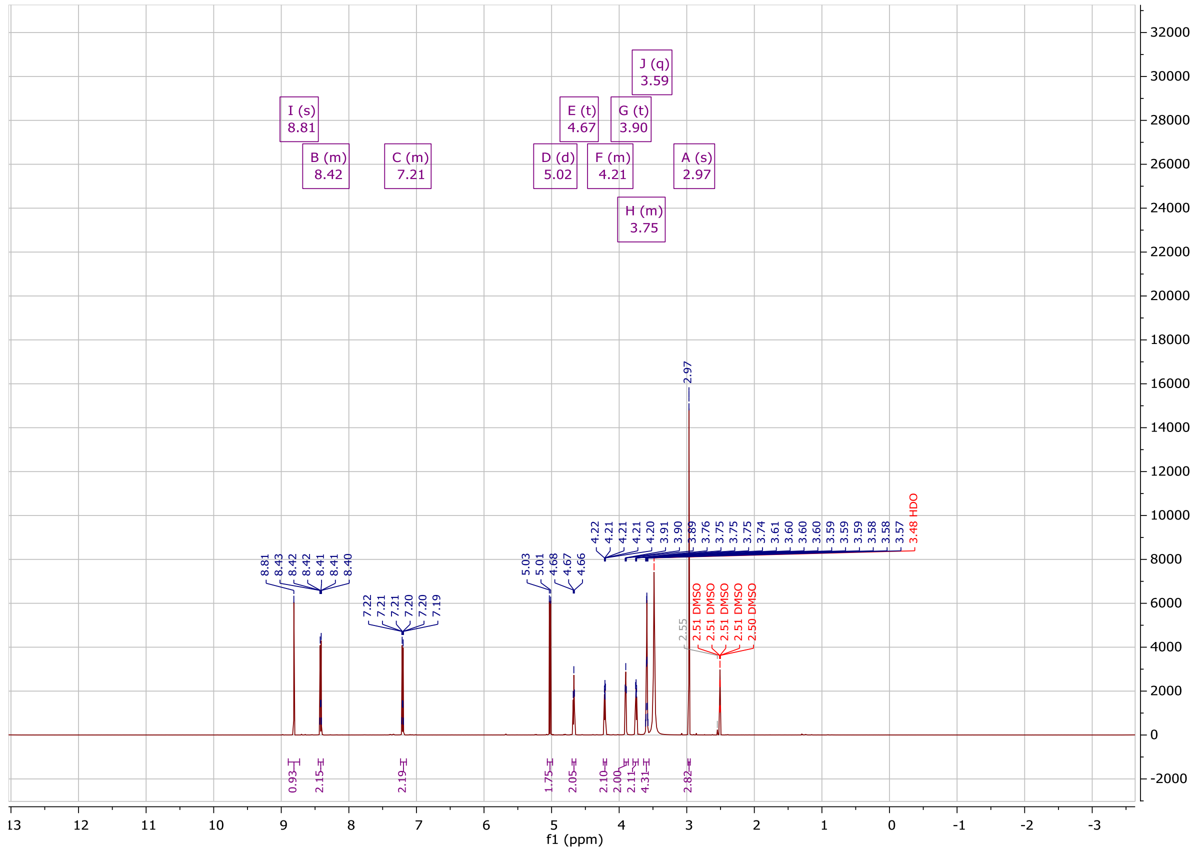
I (s) 8.81
B (m) 8.42
C (m) 7.21
D (d) 5.02
E (t) 4.67
F (m) 4.21
G (t) 3.90
H (m) 3.75
J (q) 3.59
A (s) 2.97
3.48 HDO
2.51 DMSO
f1 (ppm)

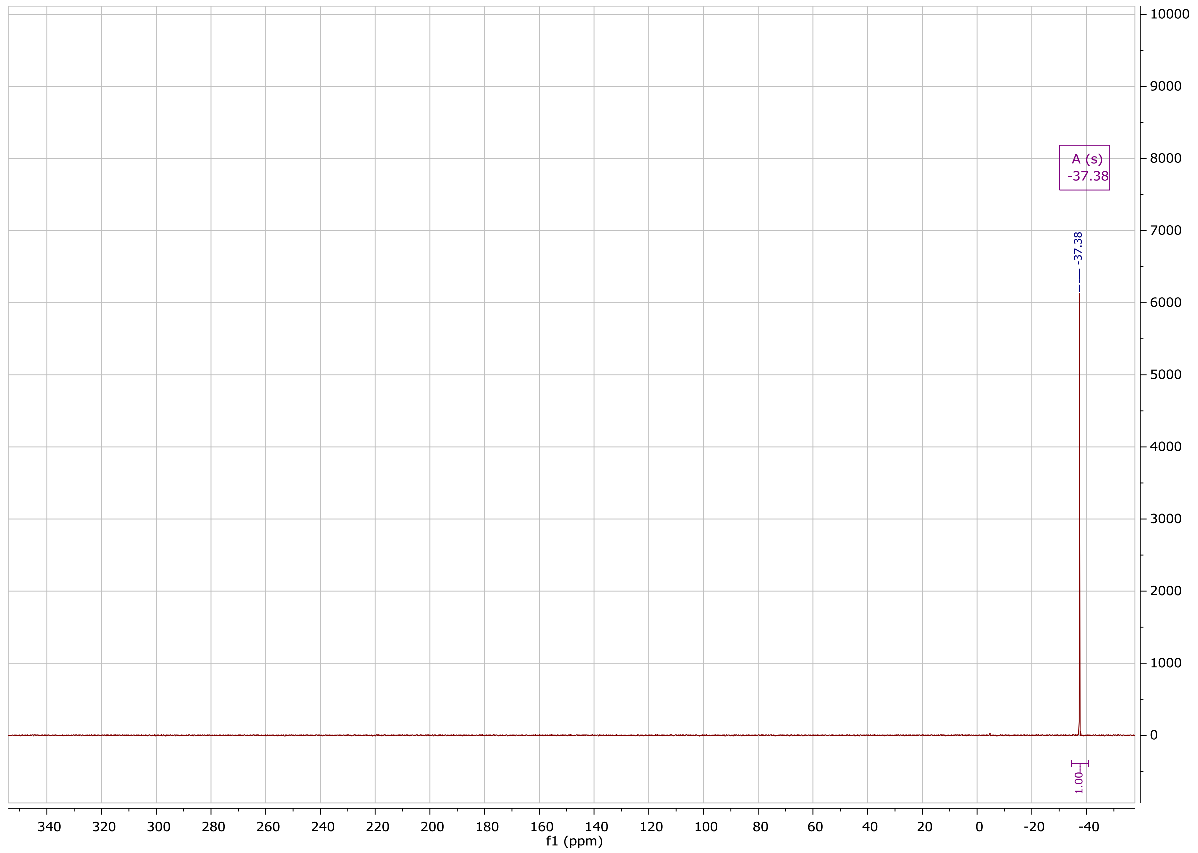
A (s) -37.38
-37.38
1.00
f1 (ppm)

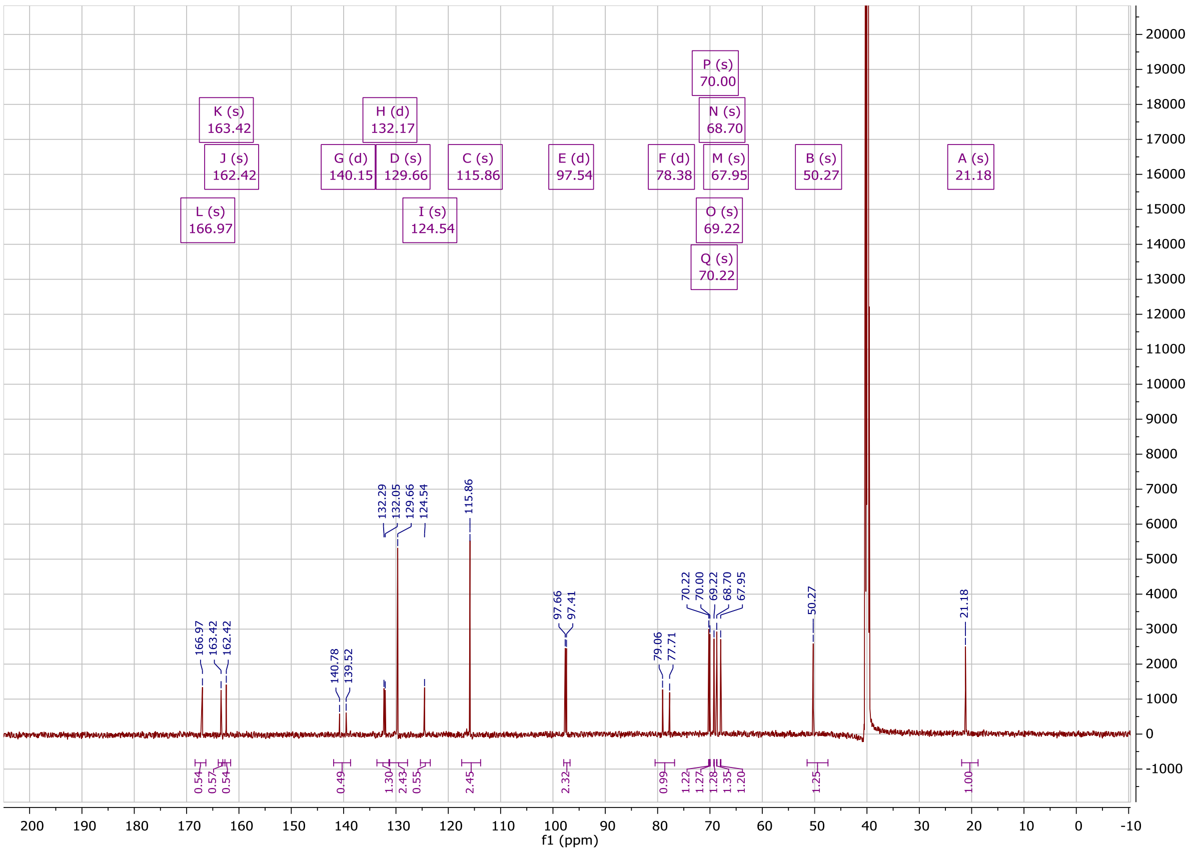

P (s) 70.00
K (s) 163.42
H (d) 132.17
N (s) 68.70
J (s) 162.42
G (d) 140.15
D (s) 129.66
C (s) 115.86
E (d) 97.54
F (d) 78.38
M (s) 67.95
B (s) 50.27
A (s) 21.18
L (s) 166.97
I (s) 124.54
O (s) 69.22
Q (s) 70.22
166.97
163.42
162.42
140.78
139.52
132.29
132.05
129.66
124.54
115.86
97.66
97.41
79.06
77.71
70.22
70.00
69.22
68.70
67.95
50.27
21.18
0.54
0.57
0.54
0.49
1.30
2.43
0.55
2.45
2.32
0.99
1.22
1.27
1.28
1.35
1.20
1.25
1.00
f1 (ppm)

4.2.8 Phenyl-Tetrazine-Ethyl-DTPO (DTPO 8):

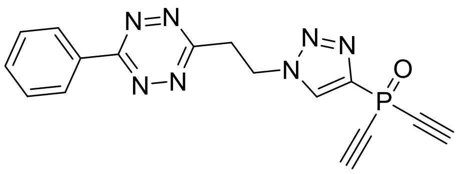

Was synthesized according to **general procedure A** from 15 mg **8a**. (7 mg, 30%).

^{1}H NMR (600 MHz, Chloroform-d) δ 8.70 – 8.53 (m, 2H), 8.31 (s, 1H), 7.79 – 7.56 (m, 4H), 5.25 (t, J = 6.8 Hz, 2H), 4.13 (t, J = 6.8 Hz, 2H), 3.33 (d, J = 11.7 Hz, 2H).
^{31}P NMR {^{1}H} (243 MHz, Chloroform-*d*) δ -35.18.
^{13}C NMR (151 MHz, Acetonitrile-*d*$_3$) δ 168.19, 165.86, 141.57 (d, *J* = 191.3 Hz), 134.12, 133.46, 132.90 (d, *J* = 35.7 Hz), 130.82, 129.20, 95.64 (d, *J* = 39.1 Hz), 78.85 (d, *J* = 205.2 Hz), 49.07, 35.91.
HRMS for $C_{16}H_{13}N_7OP^+$ $[M+H]^+$ calc.: 350.0914 Da; found: 350.0918 Da.

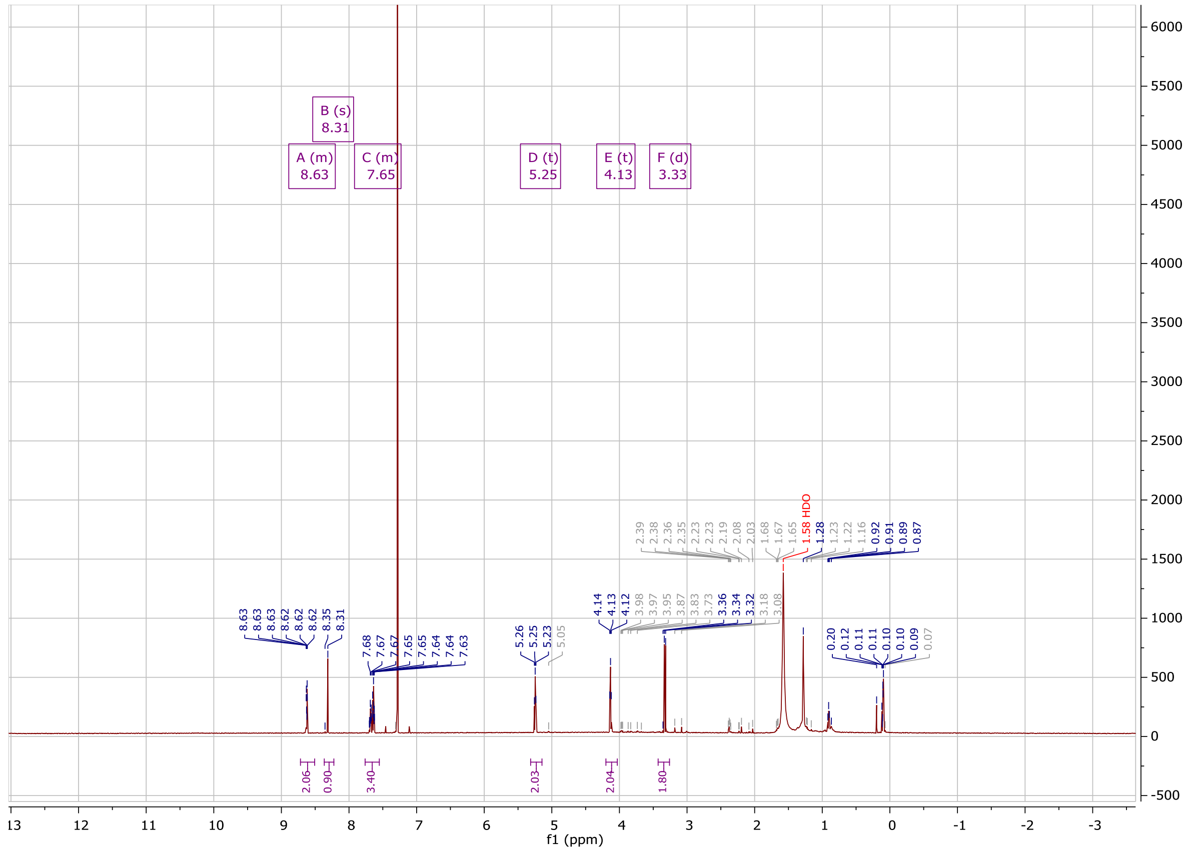

A (m)
8.63
B (s)
8.31
C (m)
7.65
D (t)
5.25
E (t)
4.13
F (d)
3.33
1.58 HDO
f1 (ppm)

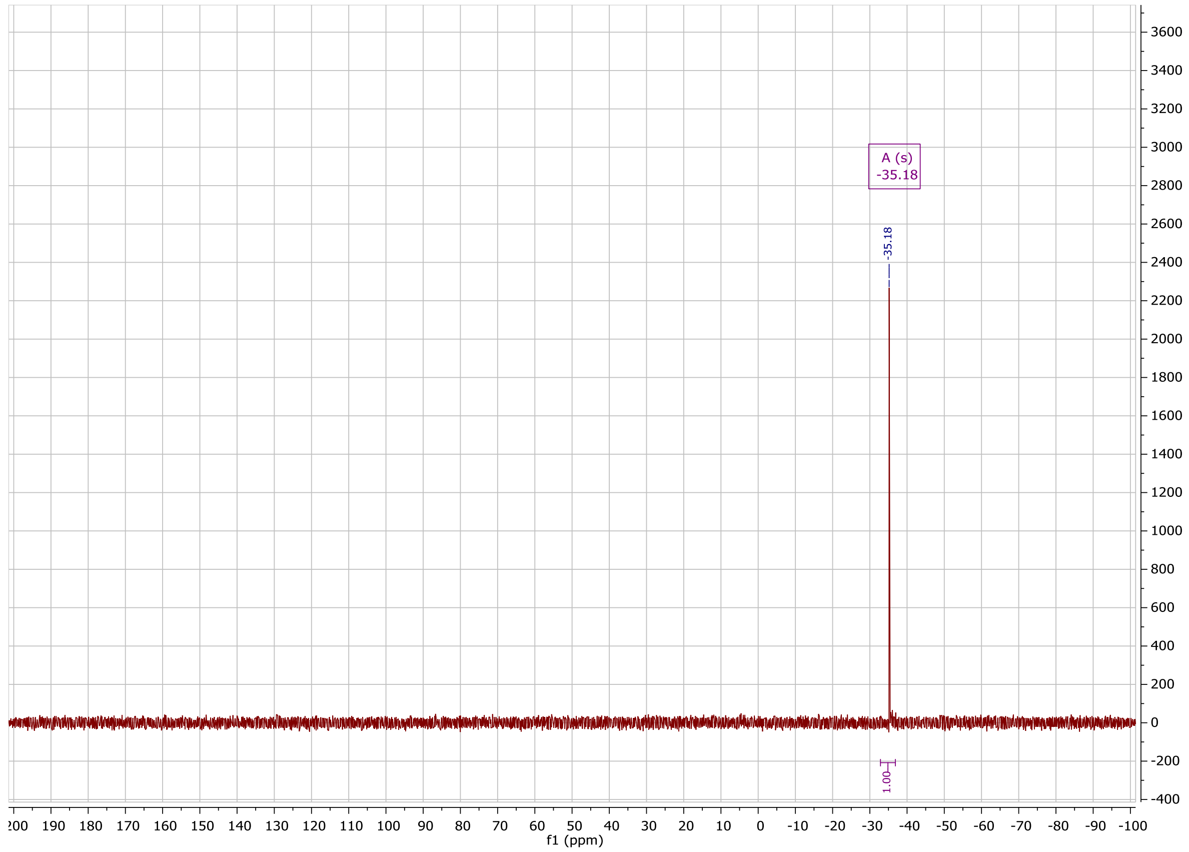

A (s)
-35.18
f1 (ppm)

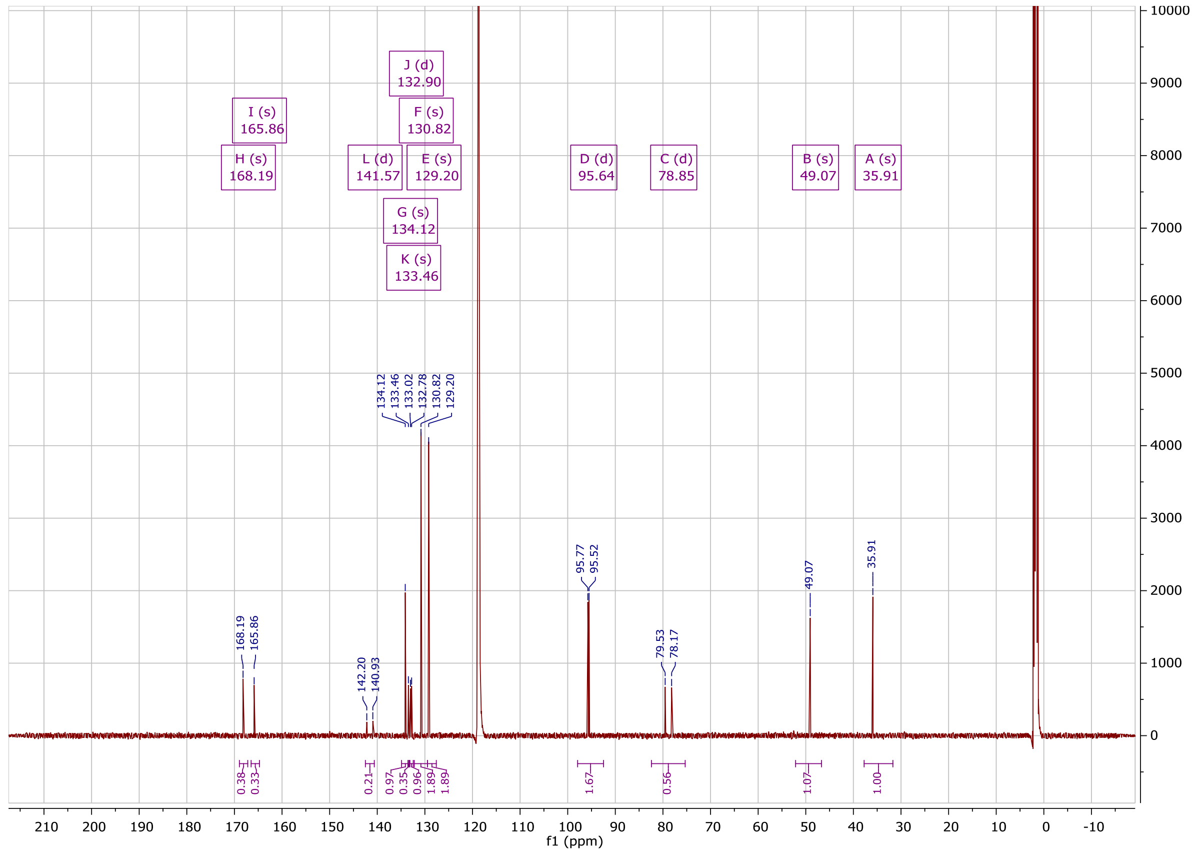

J (d) 132.90
I (s) 165.86
F (s) 130.82
H (s) 168.19
L (d) 141.57
E (s) 129.20
D (d) 95.64
C (d) 78.85
B (s) 49.07
A (s) 35.91
G (s) 134.12
K (s) 133.46
134.12 133.46 133.02 132.78 130.82 129.20
95.77 95.52
35.91
49.07
168.19 165.86
79.53 78.17
142.20 140.93
0.38 0.33 0.21 0.97 0.35 0.96 1.89 1.89 1.67 0.56 1.07 1.00
f1 (ppm)

4.3 Solid-phase peptide synthesis (SPPS)

4.3.1 General Procedure for synthesis of peptides:

Peptides were synthesized by (automated) standard fluorenylmethoxycarbonyl (Fmoc)-solid-phase peptide synthesis (SPPS) on Rink amide resin (0.1 mmol scale). Arginine was incorporated with Pbf protection, asparagine with Trt protection, Aspartic acid with OtBu protection, cysteine with Trt protection, glutamic acid with OtBu protection, Lysine with Boc protection, threonine with tBu protection, tryptophan with Boc protection and Tyrosine with tBu protection. Selective deprotection of Fmoc protected resin and Fmoc protected amino acids was achieved with 20% piperidine in DMF. Amino acid couplings were performed with 5 equiv. of amino acid, 20 equiv. of DIC and 10 equiv. of Ethyl cyanohydroxyiminoacetate (Oxyma) in DMF. All residues were coupled by single couplings with the exception or arginines which were doubly coupled. For *N*-terminus acetylation DMF:acetic anhydride:DIPEA (7:2:1, (v/v) overnight at RT. Resin cleavage was achieved by treatment with 4 ml of a TFA/TIS/H_2O/DCM (92.5:2.5:2.5:2.5 (v/v)) mixture for 1 h and precipitated in cold Et_2O/hexane (1:1). In the case of cysteine containing peptides, TFA/TIS/H_2O/EDT (92.5:2.5:2.5:2.5 (v/v)) was instead used. The crude peptides were immediately purified by semi-preparative HPLC.

4.3.2 **Ac-LYRCAK-NH₂**

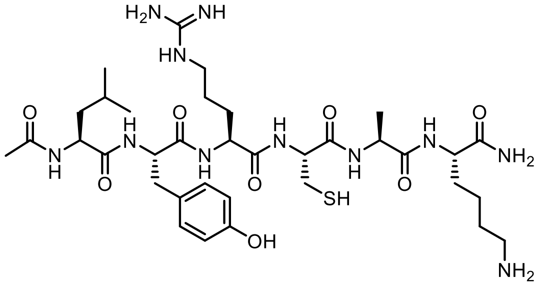

The peptide was synthesised according to the general procedure (HPLC gradient 5-30% MeCN/Water) to give the product in 14 mg, 13%. HRMS (ESI) for $C_{35}H_{60}N_{11}O_8S^+$ [M+H$^+$] calc.: 794.4342, found 794.4493.

UPLC UV trace (gradient D, 220 nm):

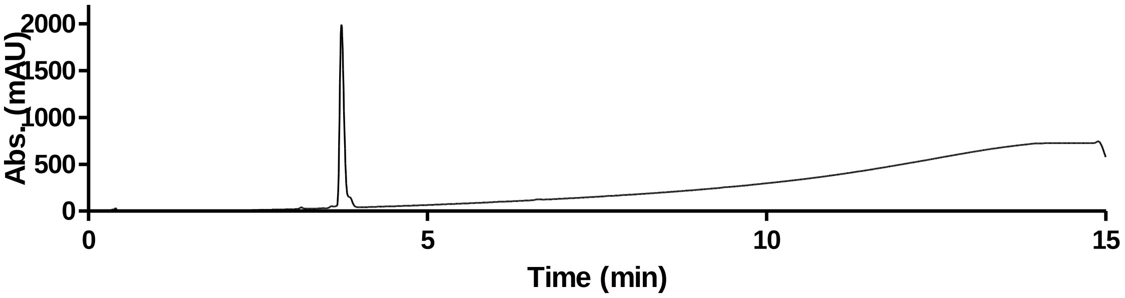

4.3.3 **N_3-GKRGDYK(N_3)-NH_2**

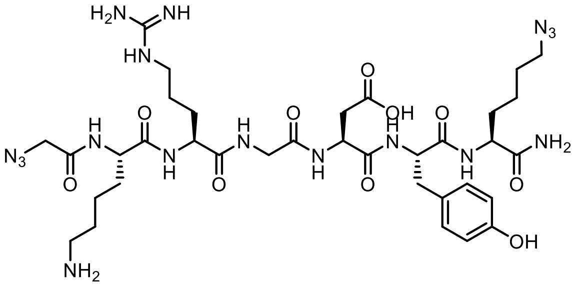

The peptide was synthesised according to the general procedure (HPLC gradient 15-95% MeCN/Water) to give the product in 23 mg, 21%. HRMS (ESI) for $C_{35}H_{56}N_{17}O_{10}^{+}$ [M+2H^{+}] calc.: 437.7232, found 437.7143.

UPLC UV trace (gradient B, 220 nm):

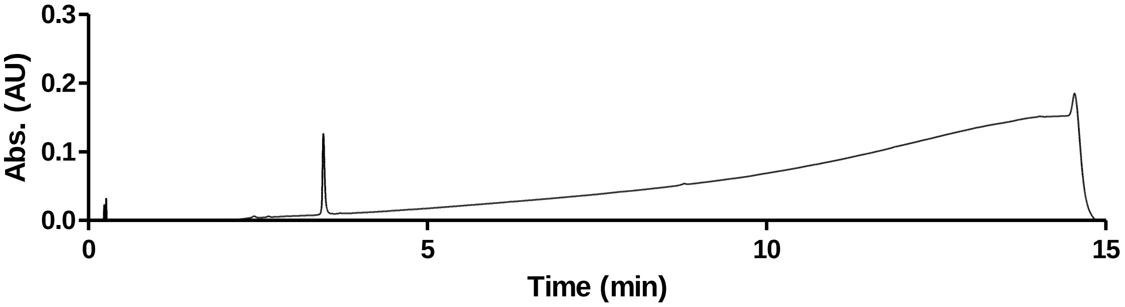

4.3.4 **N_3-GLYRAK(N_3)-NH_2**

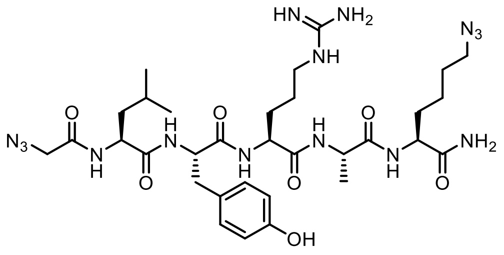

The peptide was synthesised according to the general procedure (HPLC gradient 15-95% MeCN/Water) to give the product in 32 mg, 36%. HRMS (ESI) for $C_{32}H_{52}N_{15}O_7^+$ [M+H$^+$] calc.: 758.4169, found 758.4369.

UPLC UV trace (gradient B, 220 nm):

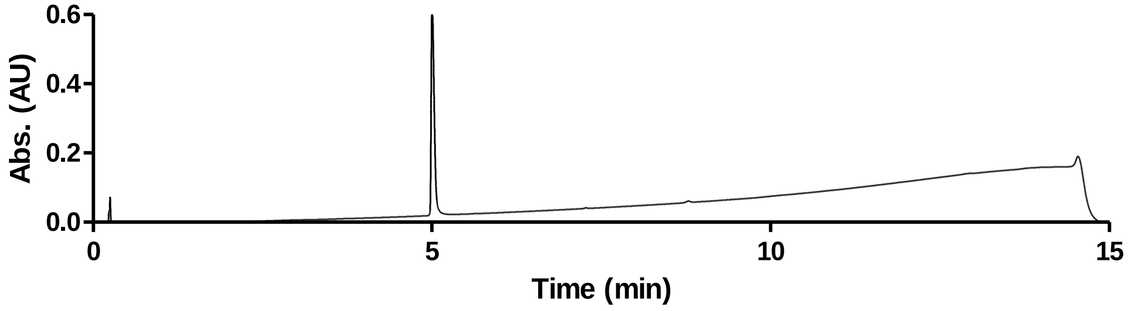

4.3.5 Ac-ETFK(N_3)DLWRLLK(N_3)EN-NH_2

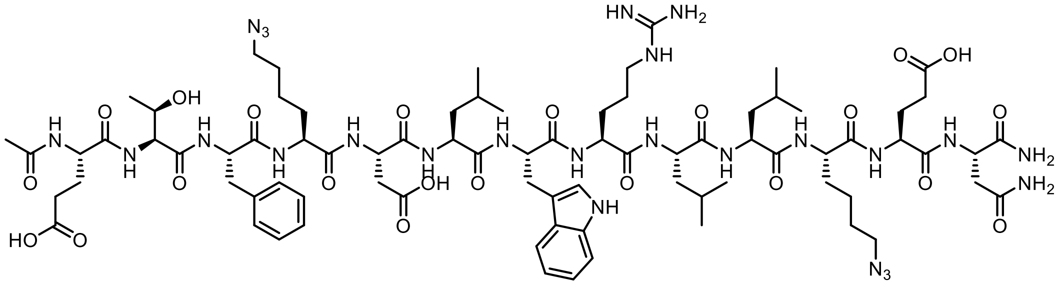

The peptide was synthesised according to the general procedure (HPLC gradient 30-95% MeCN/Water) to give the product in 47 mg, 24%. HRMS (ESI) for $C_{80}H_{122}N_{25}O_{22}^{+}$ [M+2H^{+}] calc.: 892.9632, found 892.9758.

UPLC UV trace (gradient B, 220 nm):

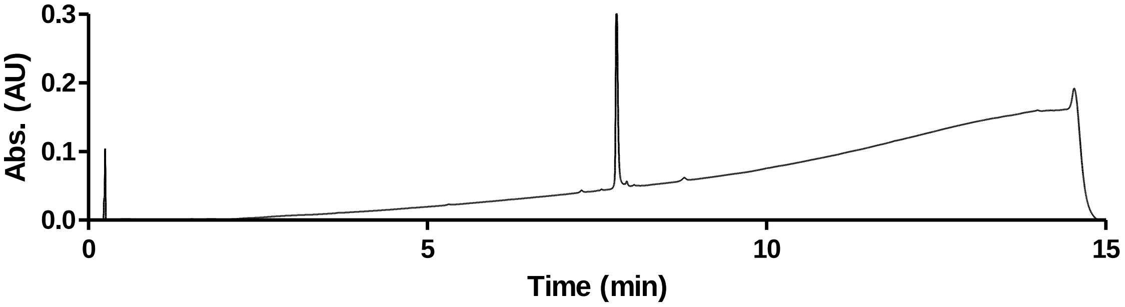

4.3.6 K(N3)rRrGrKkRrK(N3)-NH2

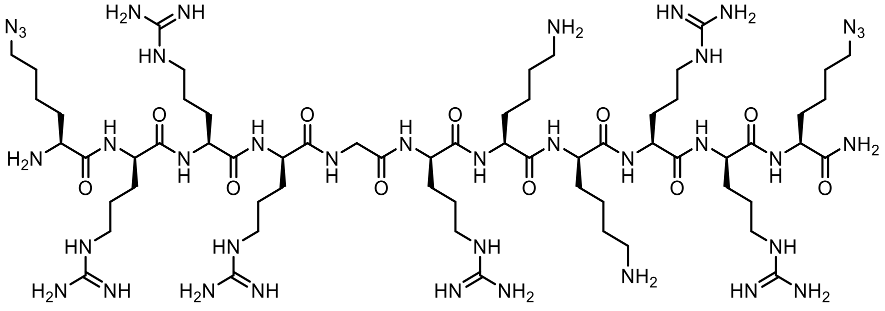

The peptide was synthesised according to the general procedure (HPLC gradient 5-95% MeCN/Water) to give the product in 41 mg, 16%. HRMS (ESI) for $C_{62}H_{123}N_{38}O_{11}^{+}$ [M+2H^{+}] calc.: 788.4984, found 788.5151.

UPLC UV trace (gradient B, 220 nm):

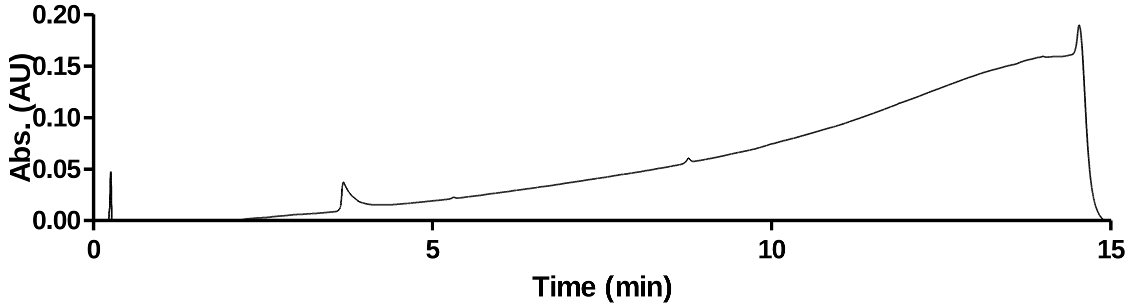

4.3.7 **GK(N3)RRRRRRRRRK(N3)G-NH2**

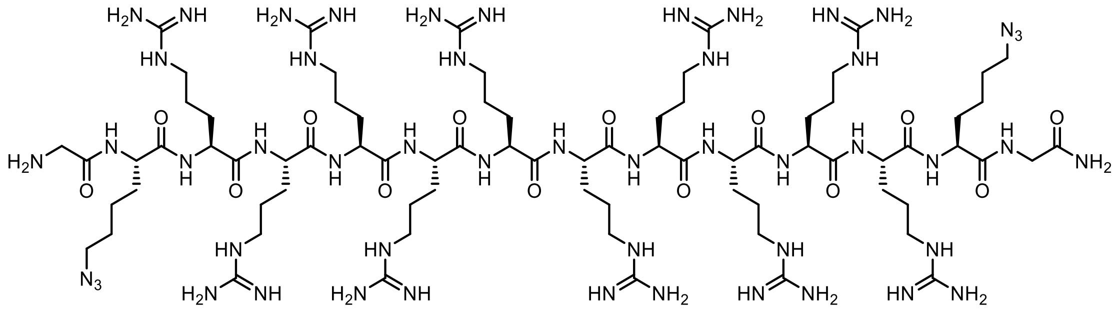

The peptide was synthesised according to the general procedure (HPLC gradient 5-95% MeCN/Water) to give the product in 35 mg, 11%. HRMS (ESI) for $C_{76}H_{150}N_{51}O_{14}^{+}$ [M+3H^{+}] calc.: 667.7578, found 667.7554.

UPLC UV trace (gradient D, 220 nm):

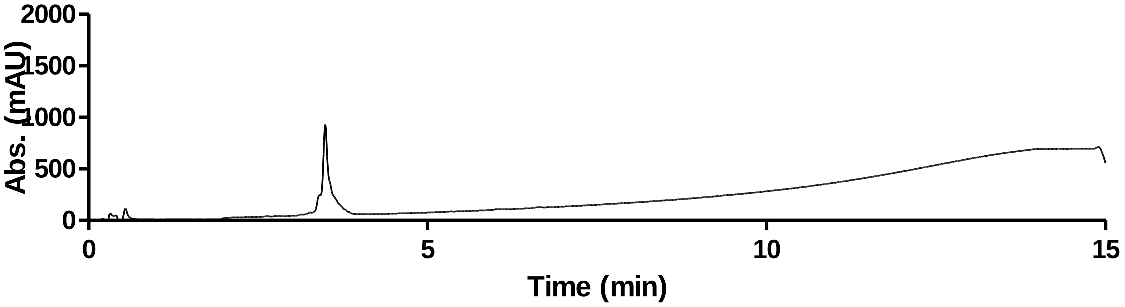

4.3.8 **GK(N_3)RRRRRRRRK(N_3)G-NH_2**

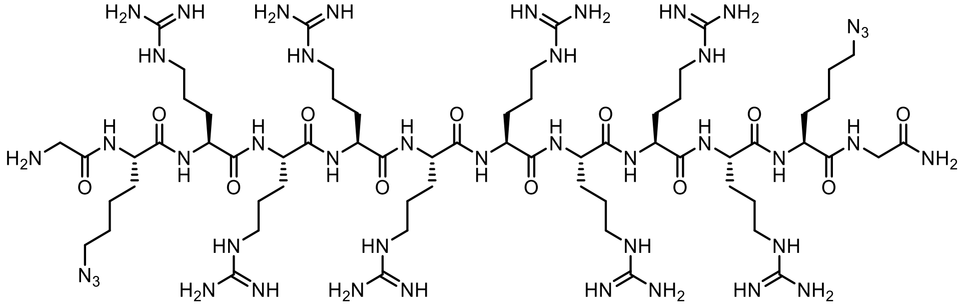

The peptide was synthesised according to the general procedure (HPLC gradient 5-95% MeCN/Water) to give the product in 34 mg, 13%. HRMS (ESI) for $C_{64}H_{126}N_{43}O_{12}^{+}$ [M+2H^{+}] calc.: 845.0319, found 845.0373.

UPLC UV trace (gradient B, 220 nm):

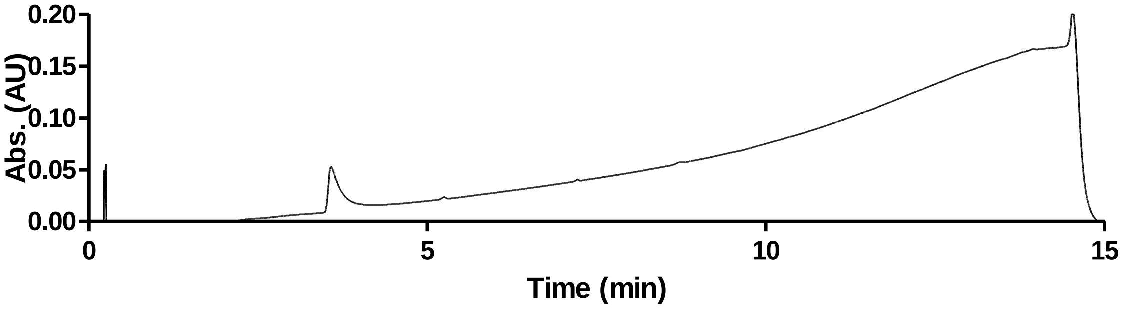

4.4 Synthesis of EDPOs and Development of the Cyclization

4.4.1 **Bis-fluorescein-EDPO (EDPO 4a)**

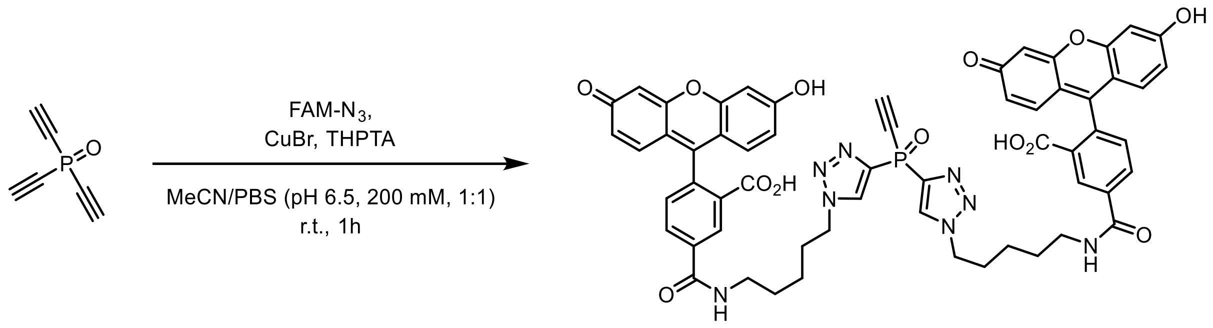

To a solution of FAM-N_3 (4.4 mg, 9 µmol, 1.8 equiv.) in 1:1 MeCN/PBS (200 mM, pH 6.5, combined volume 410 µL) was added triethynylphosphine oxide (50 µL of 100 mM stock in DMSO, 5 µmol, 1 equiv.), THPTA (20 µL of 100 mM stock in PBS, 2 µmol, 0.4 equiv., TFA salt) and CuBr (20 µL of 50 mM stock in MeCN, 1 µmol, 0.2 equiv.). The reaction was stirred for 1 hour then quenched with aqueous TFA solution (1% v/v, 250 µL) before purification by preparatory HPLC (20-95% MeCN/Water) to give the product (1.48 mg, 1.35 µmol, 27%). HRMS (ESI) for $C_{58}H_{48}N_8O_{13}P^+$ [M+H$^+$] calc.: 1095.3073, found 1095.3263. ^{31}P NMR {^{1}H} (MeOH-d$_4$, 243 MHz) δ -17.23.

UPLC UV trace (gradient B, 220 nm):

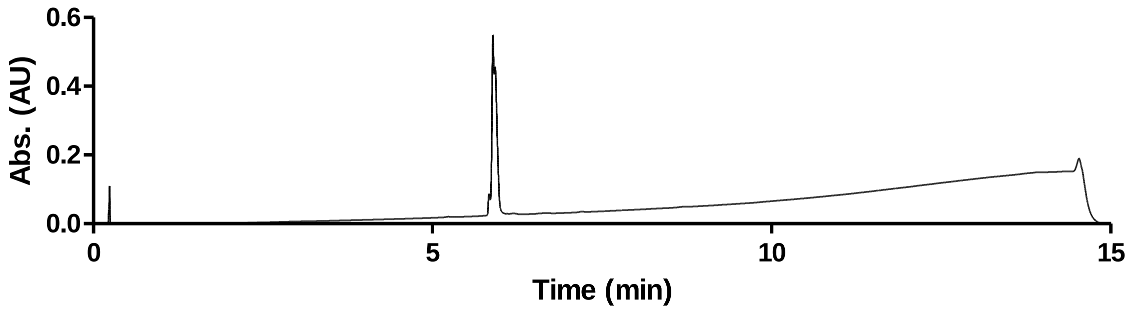

4a – ^{31}P NMR {^{1}H} (243 MHz, MeOH-d_4)

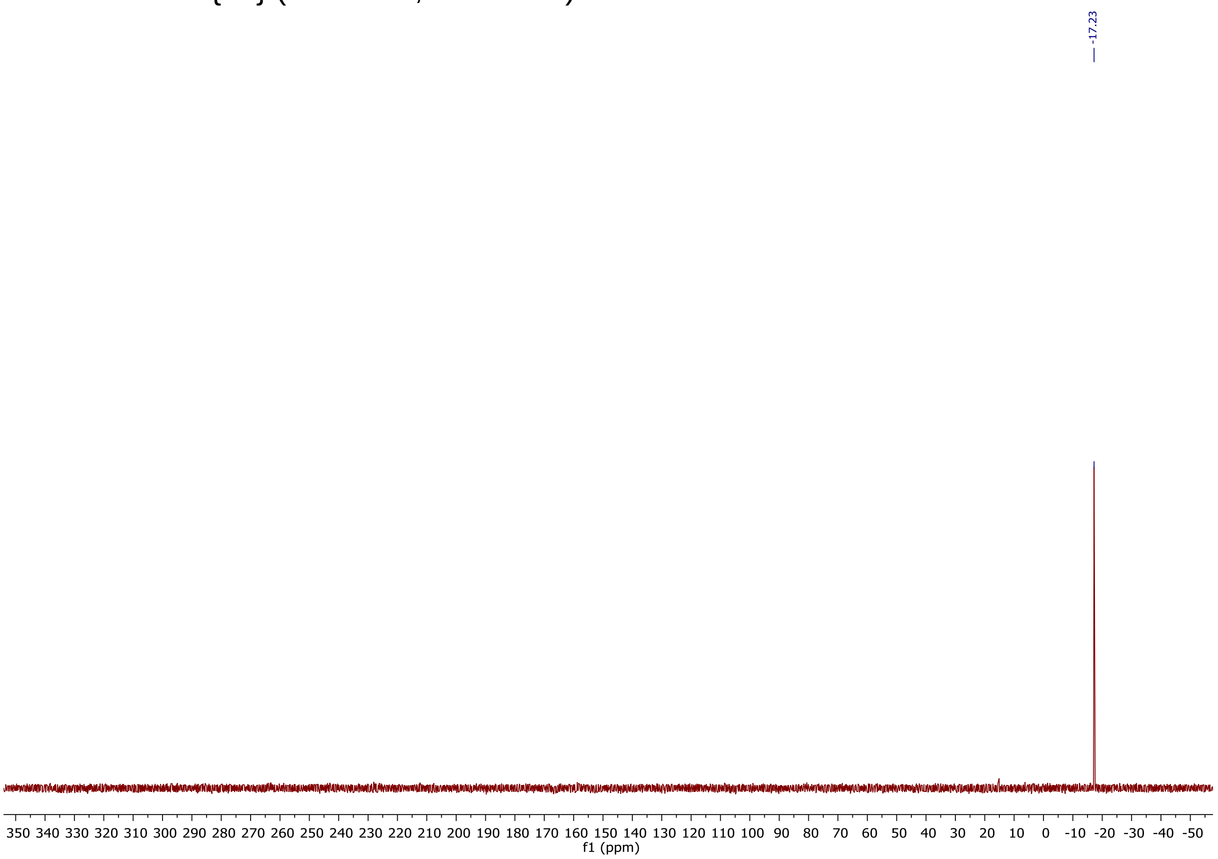

4.4.2 Optimization of the Cyclization procedure

To a solution of peptide (1 µL of 100 mM stock in DMSO, 0.1 µmol, 1 equiv.) and the desired amount of triethynylphosphine oxide (from 100 mM stock in DMSO) was added a premixed solution of CuBr (from fresh 50 mM stock in MeCN), THPTA (from 25 or 100 mM stock in 100 mM pH 5 acetate buffer, TFA salt) in one portion. The reaction mixture was stirred vigorously for 5 minutes then quenched with aqueous TFA solution (1% v/v) before analysis by UPLC (gradient E). Efficacy of the reaction measured by conversion of SM (peptide) and Area% UV comparison of product peak to that of the bis-phosphorylated by-product in the UPLC chromatogram.

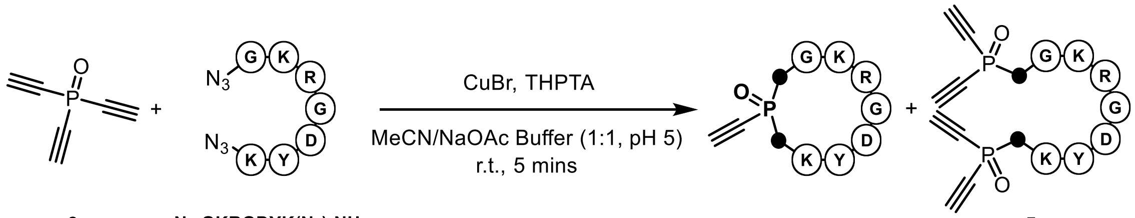

2 **N_3-GKRGDYK(N_3)-NH_2** **y** **z**

Entry	Equiv. of CuBr[a]	Equiv. of 2	Peptide conc. (µM)	Conversion (%)	y:z
1[b]	0.2	2	200	0	-
2	20	2	200	93	5.3
3	10	2	200	95	5.4
4	5	2	200	67	6.3
5	1	2	200	9	2.0
6	10	3	200	94	2.1
7	10	1	200	68	6.4
8	10	1.2	200	77	6.3
9	10	1.2	600	92	7.0
10[c]	10	1.2	1000	95	11.9

[a] THPTA always 2 equiv. with respect to CuBr. [b] MeCN/PBS (50 mM, pH 6), original conditions. [c] MeCN/Acetate Buffer (1:4) instead of 1:1.

Optimization notes

The product **P1**, was found to degrade under the reaction conditions. To avoid this, reaction times were kept to 5 minutes by using a large copper/ THPTA excess. To avoid observed product decomposition during lyophilization, reaction concentration and proportion of water in solvent mixture were increased to allow direct injection of reaction mixture into preparatory HPLC. Amount of bis-phosphorylation was reduced by lowering phosphine oxide equivalents. Once purified, EDPO products could be safely freeze-dried and were stored at -20 °C. Decomposition is usually seen to $R_2P(O)$-OH over time. Although the formation of multimers/ polymers cannot be excluded, masses corresponding to the formation of dimers were not seen.

4.4.3 **Confirmation of the identity of the products**

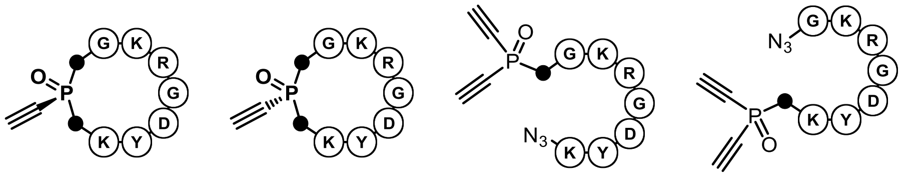

I **II** **III** **IV**

Figure S39: possible reaction products with the same mass.

All reactions resulted in the formation of two compounds of the product mass although they were not always easily separable by preparatory HPLC. These two compounds could feasibly be two diastereoisomers (**I** and **II** in **Figure S39**, chiral center at phosphorous) or uncycled peptides functionalized at one azide or another (**III** or **IV** in **Figure S39**). ^{31}P-NMR data for the products came in the region of -15 ppm which fits better with the shift of bis-triazole compound **4a** than with bis-ethynyl compounds **4-8**. Further confirmation came by separately reacting both isolated **P1** and **P1'** with 100 equivalents of glutathione. After 20 min the starting material was completely consumed and a single product of m/z 652 was found. This matches the expected mass of product **V** (**Figure S40 & S41**). No peaks of m/z 806 were found, the expected mass of product **VI** (**Figure S40 & S41**) This data agrees with the identification of the products as cyclized diastereoisomers.

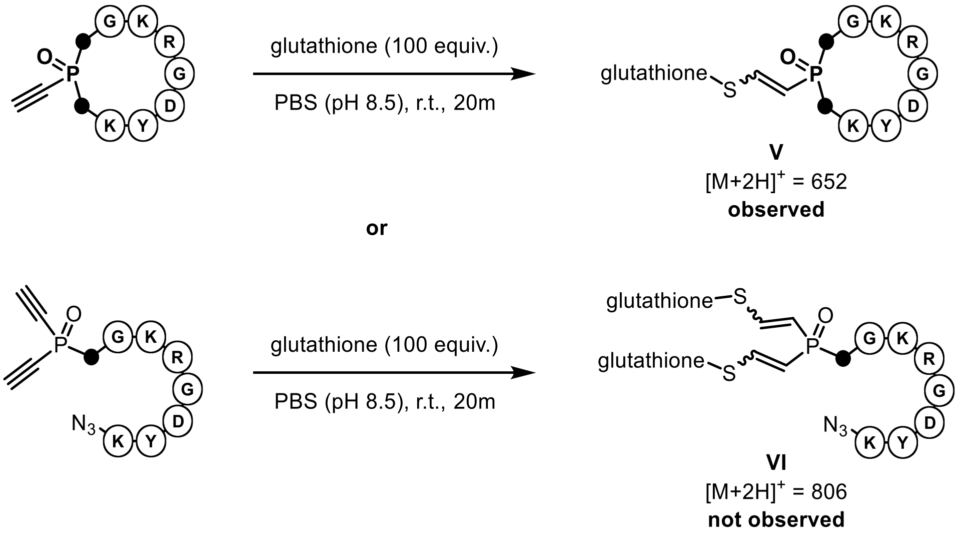

Figure S40: theoretical reaction products of the addition of glutathione to both possible product classes which revealed only the single addition product to be formed.

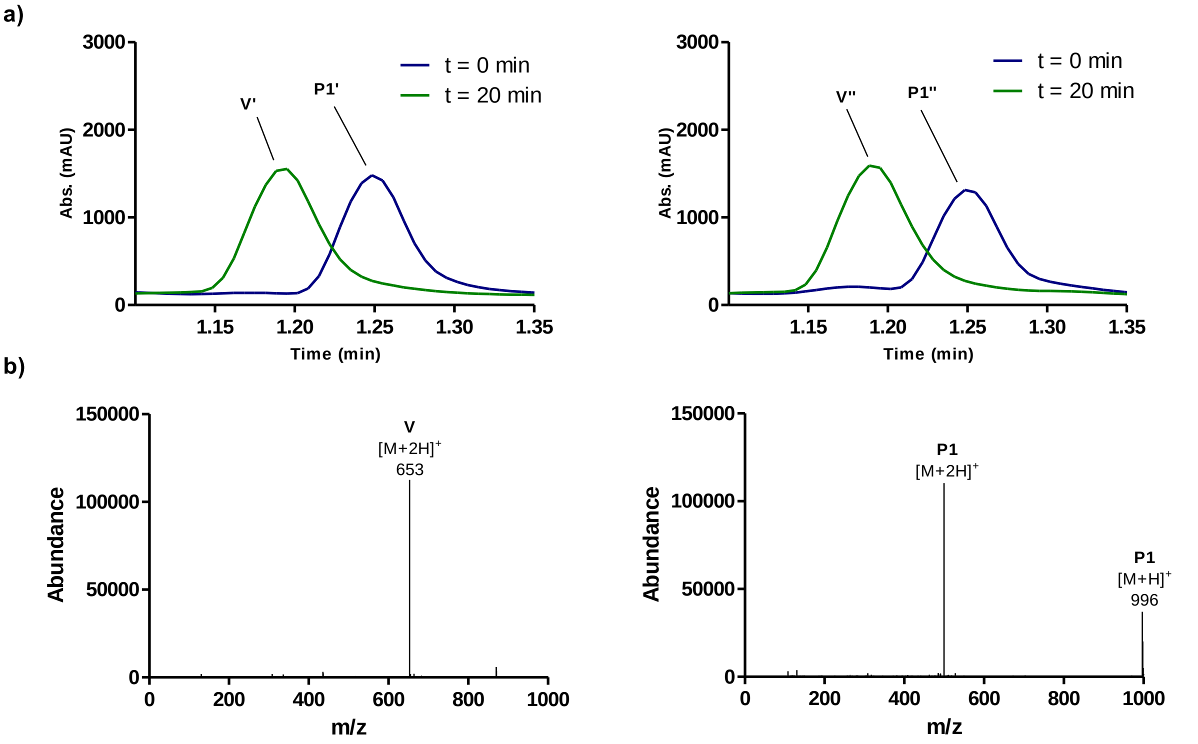

Figure S41: a) zoomed-in UPLC traces showing the conversion of compound **P1'** (left) and **P1''** (right) at t = 0 min to their corresponding glutathione adducts **V'** and **V''** at t = 20 min. b) Corresponding mass spectra of the **V** peaks (left) and **P1** peaks (right). The spectra are the same for both **P1'** and **P1''**. No peak at m/z 806 was detected in either case.

4.4.4 **General Cyclization procedure**

To a solution of peptide (20 µL of 100 mM stock in DMSO, 2 µmol, 1 equiv.) and triethynylphosphine oxide (24 µL of 100 mM stock in DMSO, 2.4 µmol, 1.2 equiv.) was added a premixed solution of CuBr (400 µL of 50 mM stock in MeCN, 20 µmol, 10 equiv.), THPTA (1.6 mL of 25 mM stock in 100 mM pH 5 acetate buffer, 20 µmol, 20 equiv., TFA salt) in one portion. The reaction mixture was stirred vigorously for 5 minutes then quenched with aqueous TFA solution (1% v/v, 1.6 mL) before *immediate* purification by preparatory HPLC.

4.4.5 **Ethynyl-PO-c(N_3-GKRGDYK(N_3)-NH_2 (EDPO P1)**

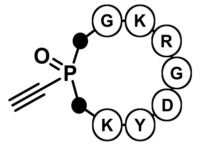

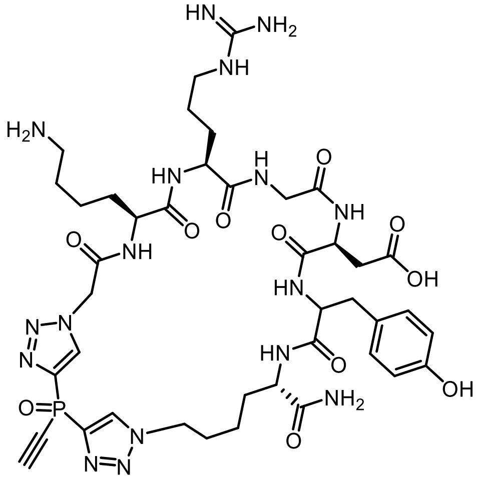

The peptide was cyclized according to general procedure (HPLC gradient 5-30% MeCN/Water) to give **P1** as a separable mixture of two diastereoisomers (1.01 mg, 0.83 µmol, 41%, dr 1:1.1).
For the earlier eluting diastereoisomer **P1'**: HRMS (ESI) for $C_{41}H_{59}N_{17}O_{11}P^+$ [M+H$^+$] calc.: 996.4313, found 996.4462. ^{31}P NMR {^1H} (H_2O, 243 MHz) δ -16.79.

UPLC UV trace (gradient D, 220 nm):

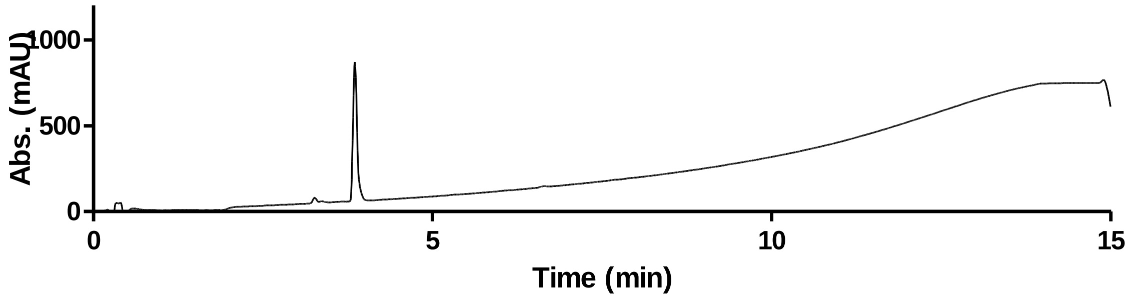

For the later eluting diastereoisomer **P1''**: HRMS (ESI) for $C_{41}H_{59}N_{17}O_{11}P^+$ [M+H$^+$] calc.: 996.4313, found 996.4590. ^{31}P NMR {^1H} (H_2O, 243 MHz) δ -16.38.

UPLC UV trace (gradient D, 220 nm):

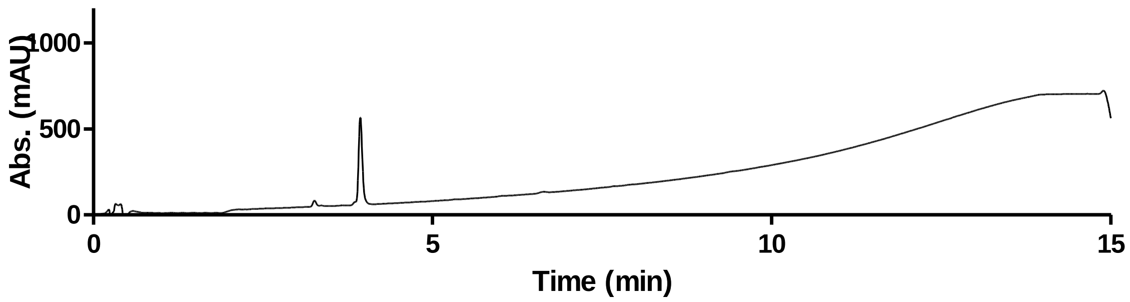

P1' – ^{31}P NMR {^{1}H} (243 MHz, H_2O)

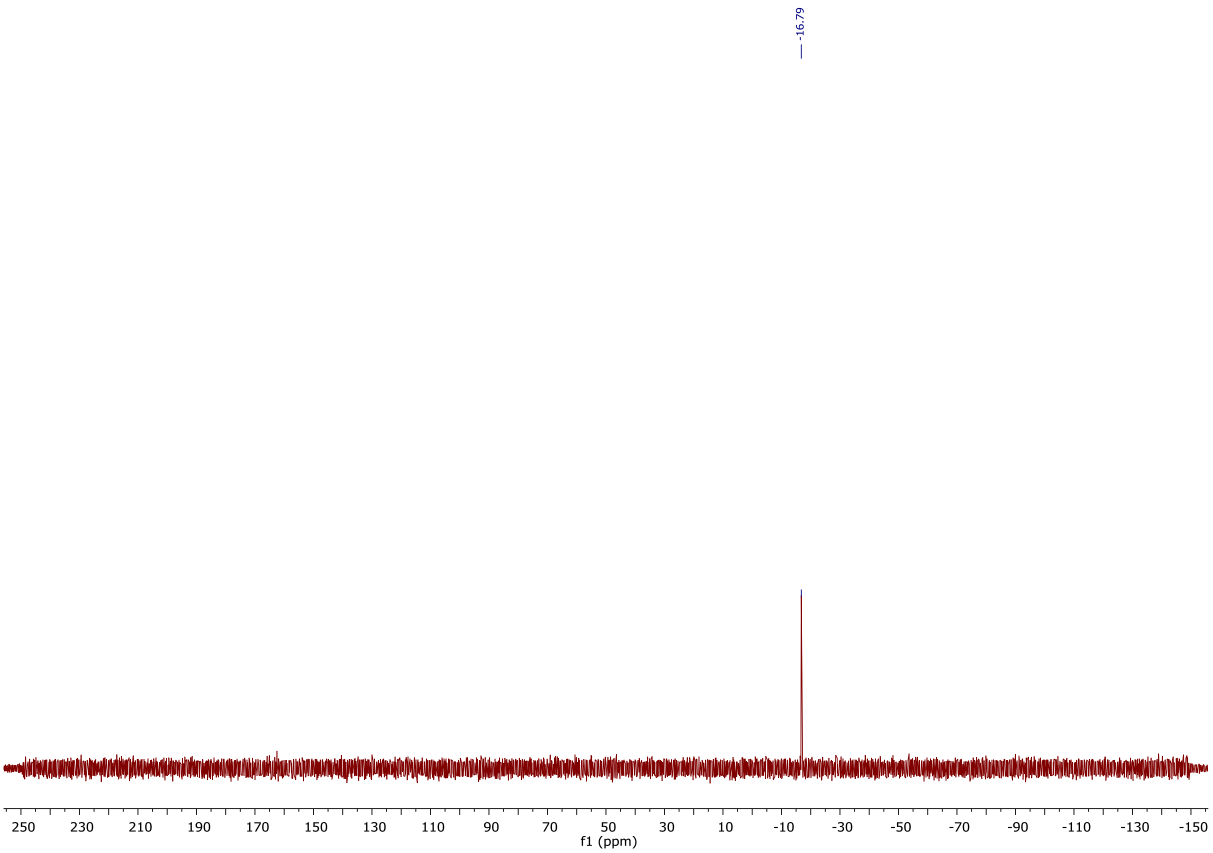

P1'' – ^{31}P NMR {^{1}H} (243 MHz, H_2O)

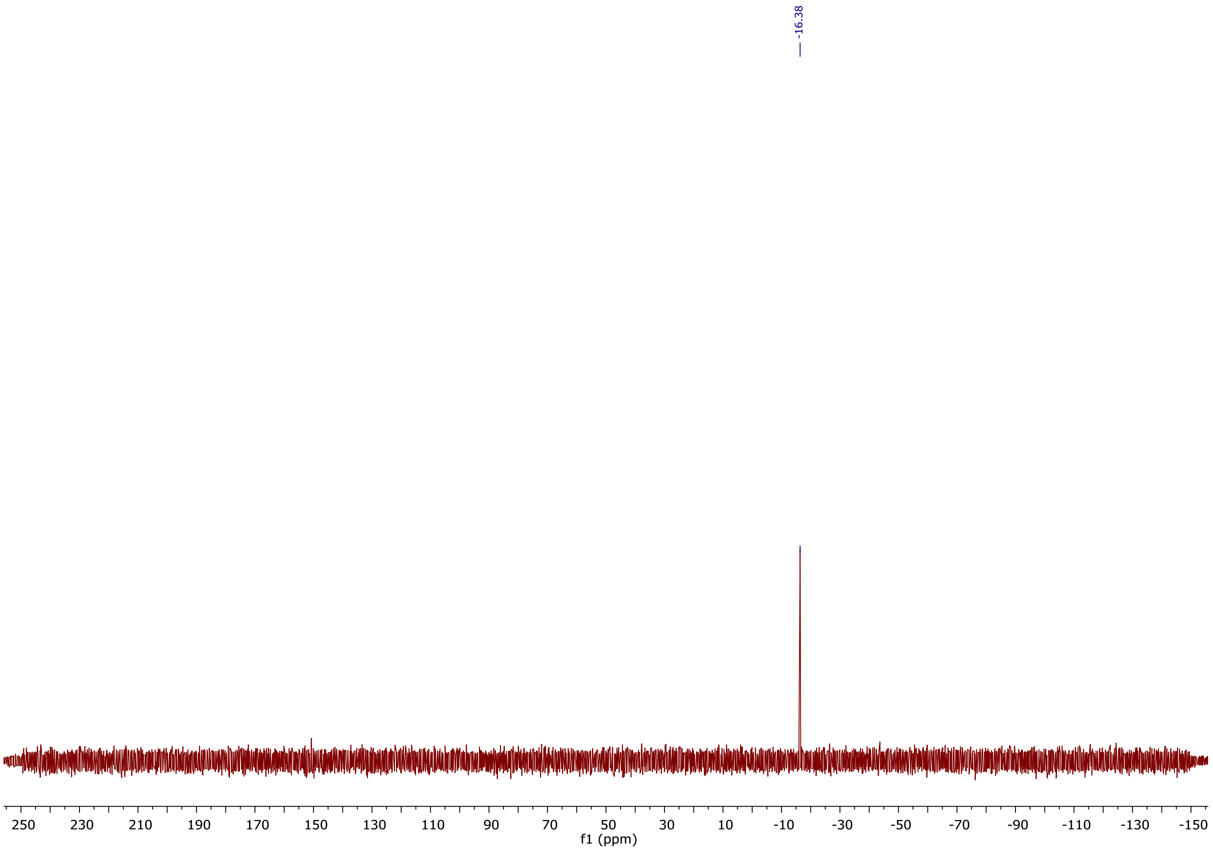

4.4.6 **Ethynyl-PO-c(N_3-GLYRAK(N_3)-NH_2) (EDPO P2)**

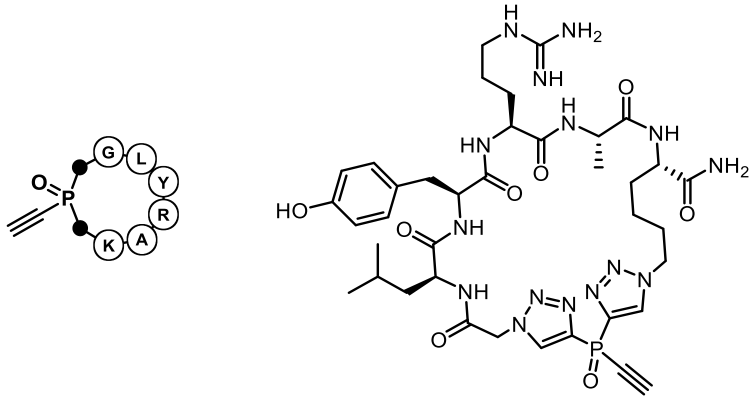

The peptide was cyclized according to general procedure (HPLC gradient 10-30% MeCN/Water) to give **P2** as a separable mixture of two diastereoisomers (0.78 mg, 0.78 µmol, 39%, dr 1:1.4).
For the earlier eluting diastereoisomer **P2'**: HRMS (ESI) for $C_{38}H_{55}N_{15}O_8P^+$ [M+H$^+$] calc.: 880.4090, found 880.4149. ^{31}P NMR {^{1}H} (H_2O, 243 MHz) δ -14.91.

UPLC UV trace (gradient D, 220 nm):

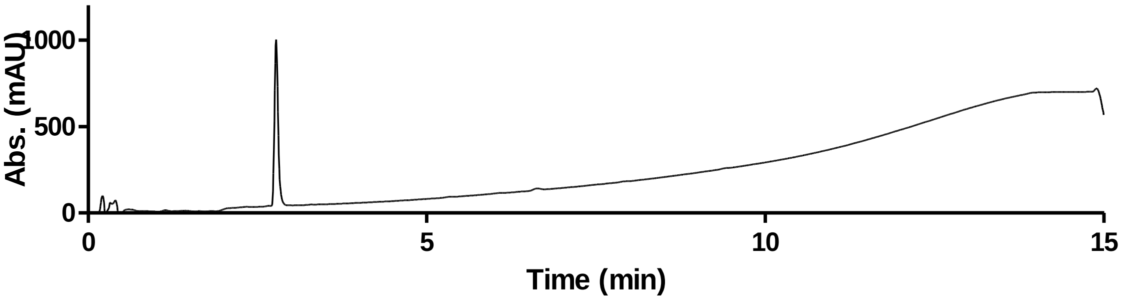

For the later eluting diastereoisomer: **P2''**: HRMS (ESI) for $C_{38}H_{55}N_{15}O_8P^+$ [M+H$^+$] calc.: 880.4090, found 880.4392. ^{31}P NMR {^{1}H} (H_2O, 243 MHz) δ -15.99.

UPLC UV trace (gradient D, 220 nm):

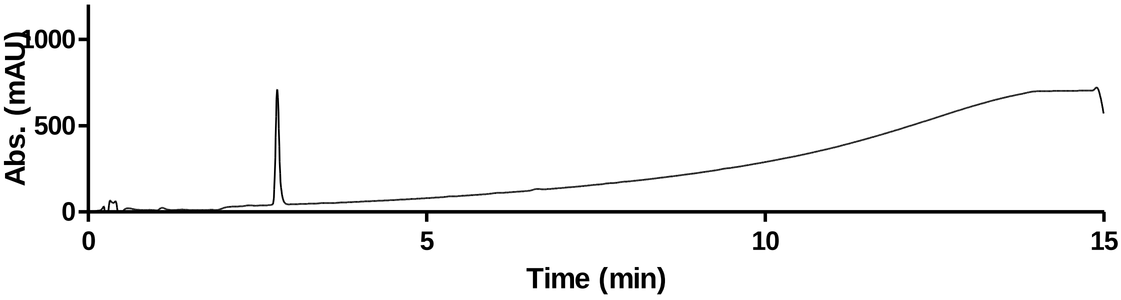

P2’ – ^{31}P NMR {^{1}H} (243 MHz, H_2O)

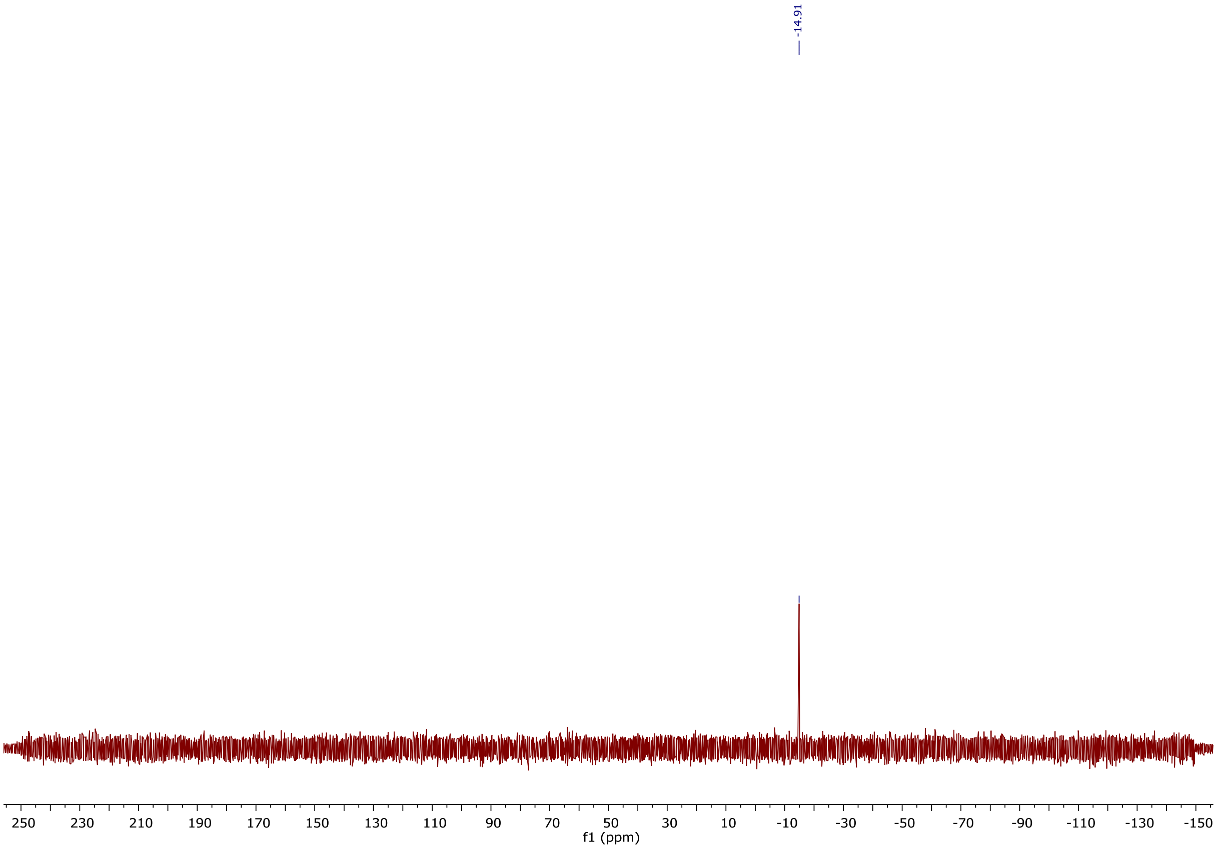

P2’’ – ^{31}P NMR {^{1}H} (243 MHz, H_2O)

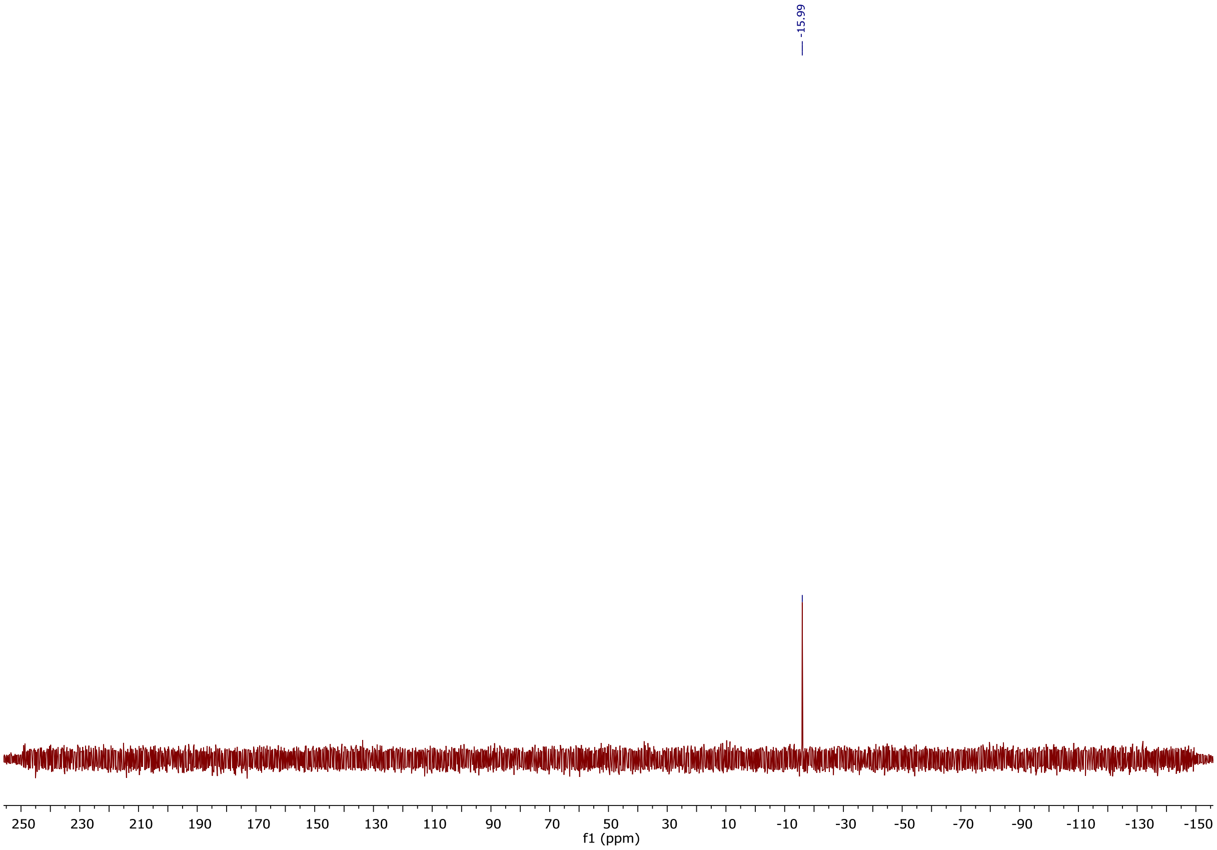

4.4.7 **Ethynyl-PO-c(Ac-ETFK(N_3)DLWRLLK(N_3)EN-NH_2) (EDPO P3)**

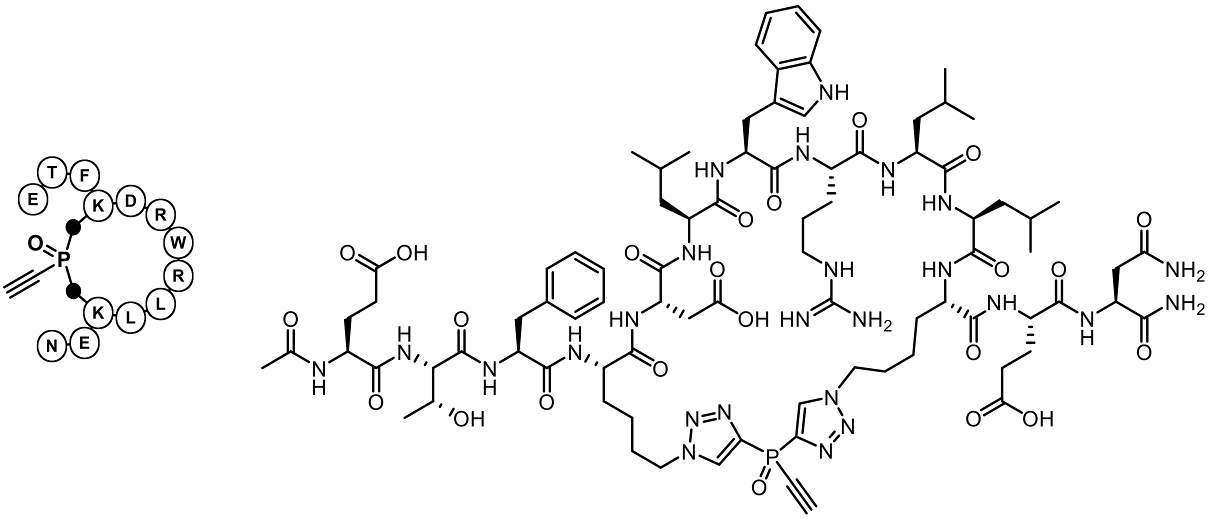

The peptide was cyclized according to general procedure (HPLC gradient 25-95% MeCN/Water) to give **P3** as a separable mixture of two diastereoisomers (0.97 mg, 0.48 µmol, 24%, dr 1:1.2).
For the earlier eluting diastereoisomer **P3'**: HRMS (ESI) for $C_{86}H_{125}N_{25}O_{23}P^+$ [M+2H$^+$] calc.: 953.9593, found 953.9781. ^{31}P NMR {^1H} (H_2O/MeCN 1:1, 243 MHz) δ -16.61.

UPLC UV trace (gradient D, 220 nm):

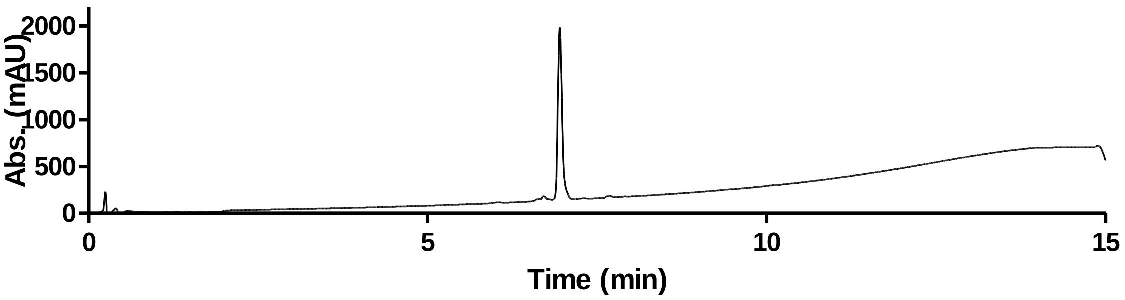

For the later eluting diastereoisomer **P3''**: HRMS (ESI) for $C_{86}H_{125}N_{25}O_{23}P^+$ [M+2H$^+$] calc.: 953.9593, found 953.9781. ^{31}P NMR {^1H} (H_2O/MeCN 1:1, 243 MHz) δ -16.75.

UPLC UV trace (gradient D, 220 nm):

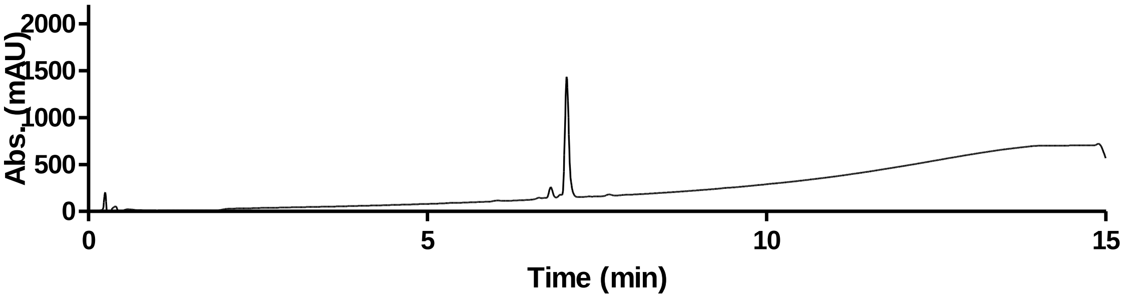

P3' – ^{31}P NMR {^{1}H} (243 MHz, H_2O/MeCN (1:1))

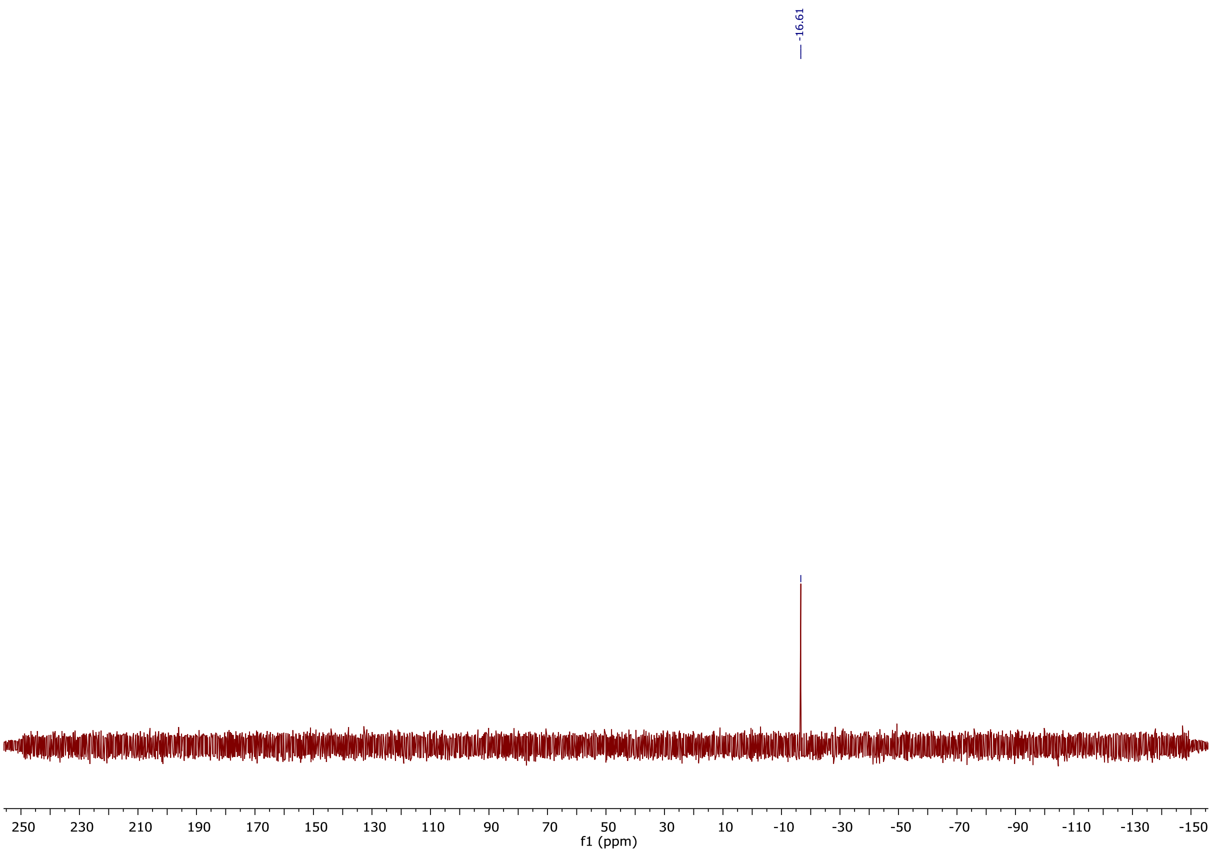

P3'' – ^{31}P NMR {^{1}H} (243 MHz, H_2O/MeCN (1:1))

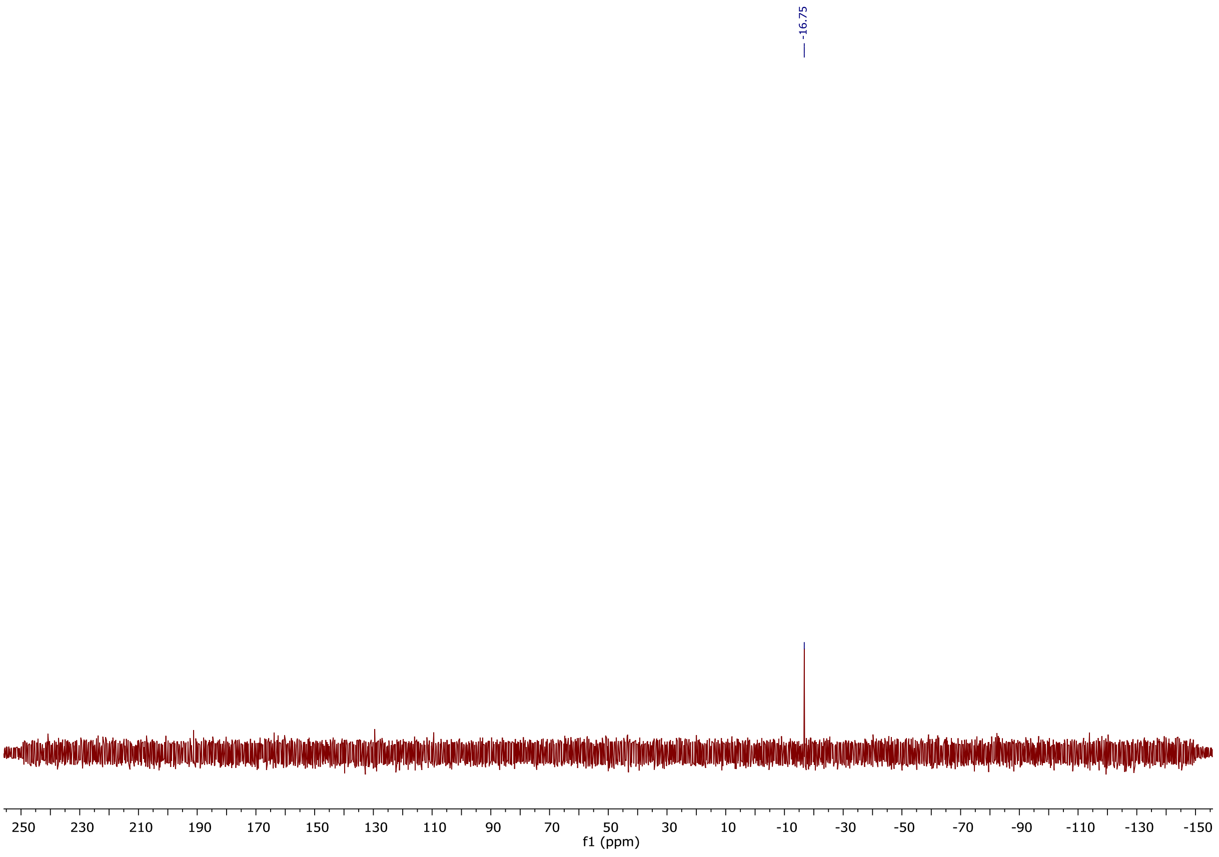

4.4.8 **Ethynyl-PO-c(K(N_3)rRrGrKkRrK(N_3)-NH_2) (EDPO P4)**

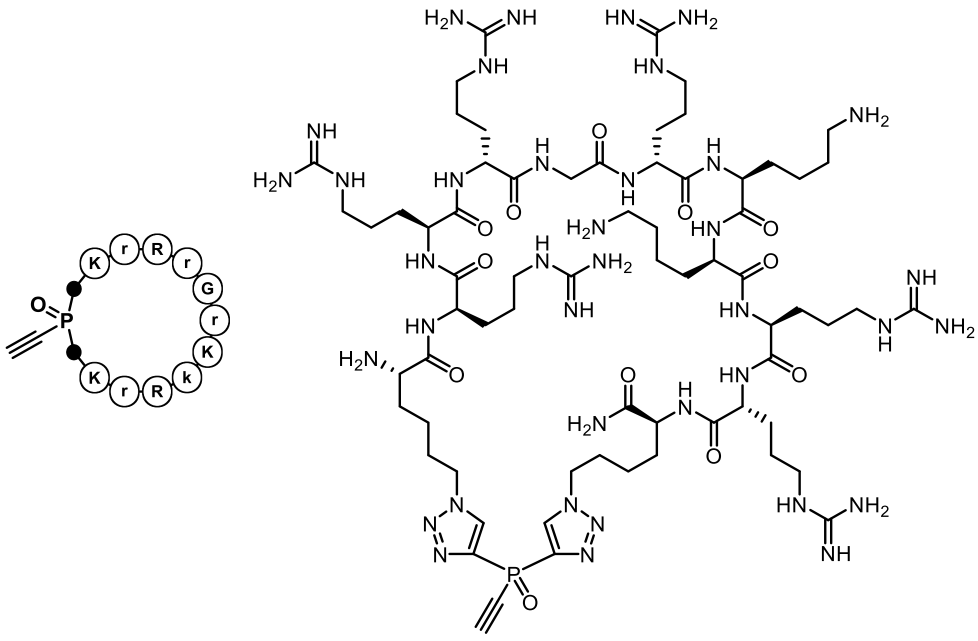

The peptide was cyclized according to general procedure (HPLC gradient 10-30% MeCN/Water) to give **P4** as an inseparable mixture of two diastereoisomers (2.14 mg, 0.79 µmol, 39%, dr 1:1.2). HRMS (ESI) for $C_{68}H_{125}N_{38}O_{12}P^+$ [M+2H$^+$] calc.: 849.5111, found 849.5047. ^{31}P NMR {^{1}H} (H_2O, 243 MHz) δ -15.38, -15.72.

UPLC UV trace (gradient D, 220 nm):

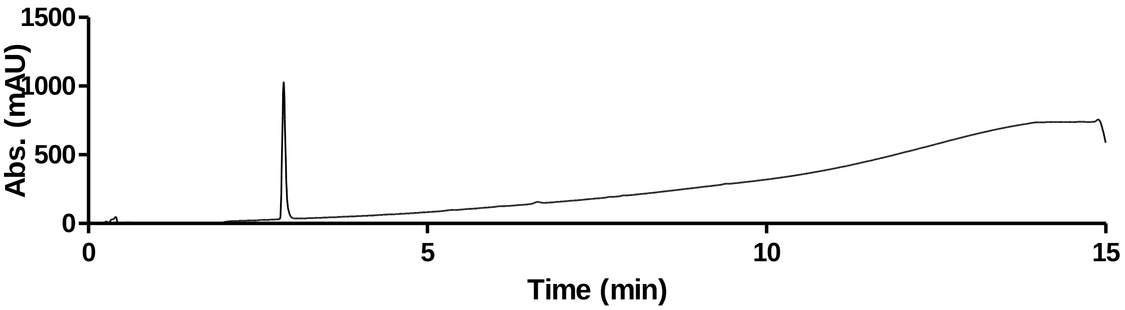

P4 – ^{31}P NMR {^{1}H} (243 MHz, H_2O)

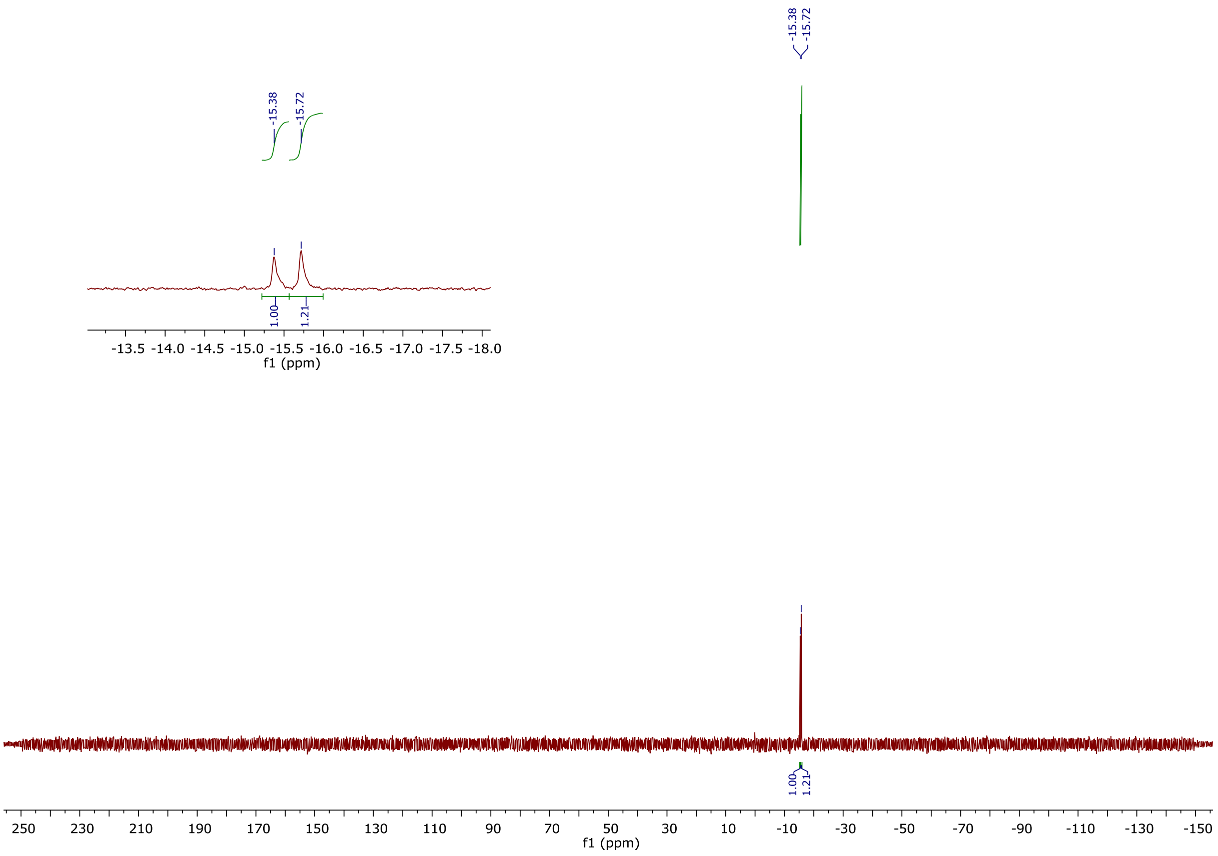

4.4.9 **Ethynyl-PO-c(GK(N_3)RRRRRRRRRRK(N_3)G-NH_2) (EDPO P5)**

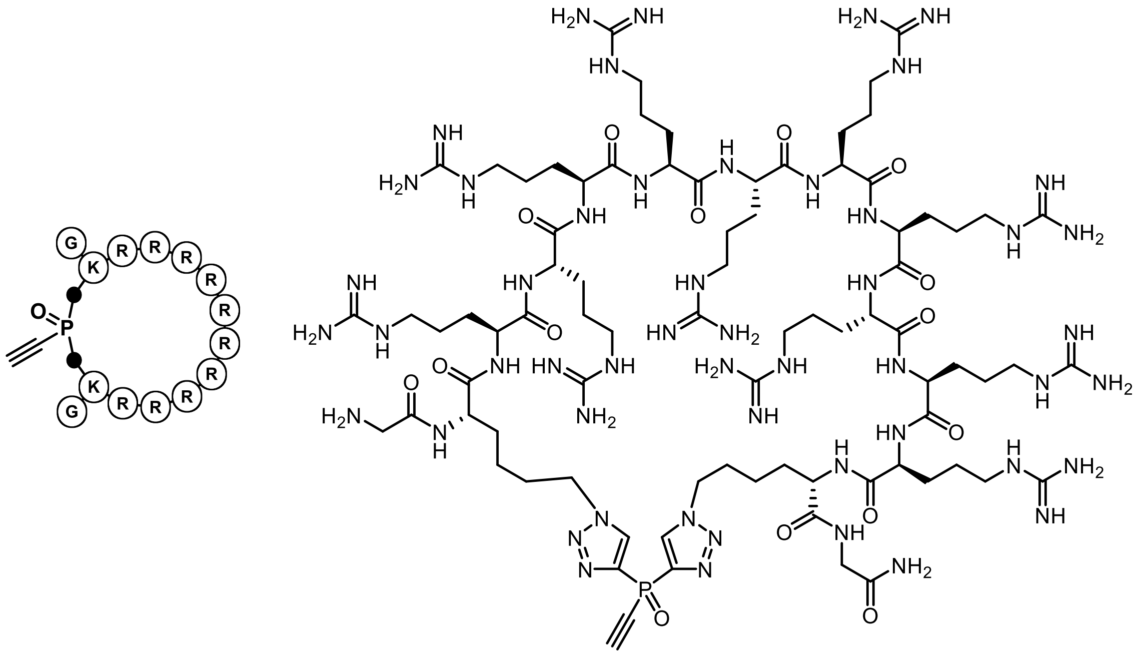

The peptide was cyclised according to general procedure but on a reduced 1.1 µmol scale (HPLC gradient 5-30% MeCN/Water) to give **P5** as an inseparable mixture of two diastereoisomers (0.66 mg, 0.21 µmol, 18%, dr 1:1.2). HRMS (ESI) for $C_{82}H_{153}N_{51}O_{15}P^+$ [M+3H$^+$] calc.: 708.4218, found 708.4162. ^{31}P NMR {^{1}H} (H_2O, 243 MHz) δ -15.81, -15.82.

UPLC UV trace (gradient D, 220 nm):

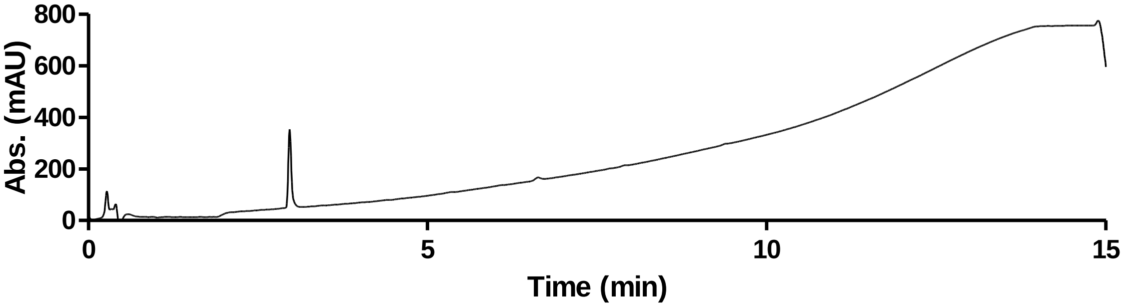

P5 – ^{31}P NMR {^{1}H} (243 MHz, H_2O)

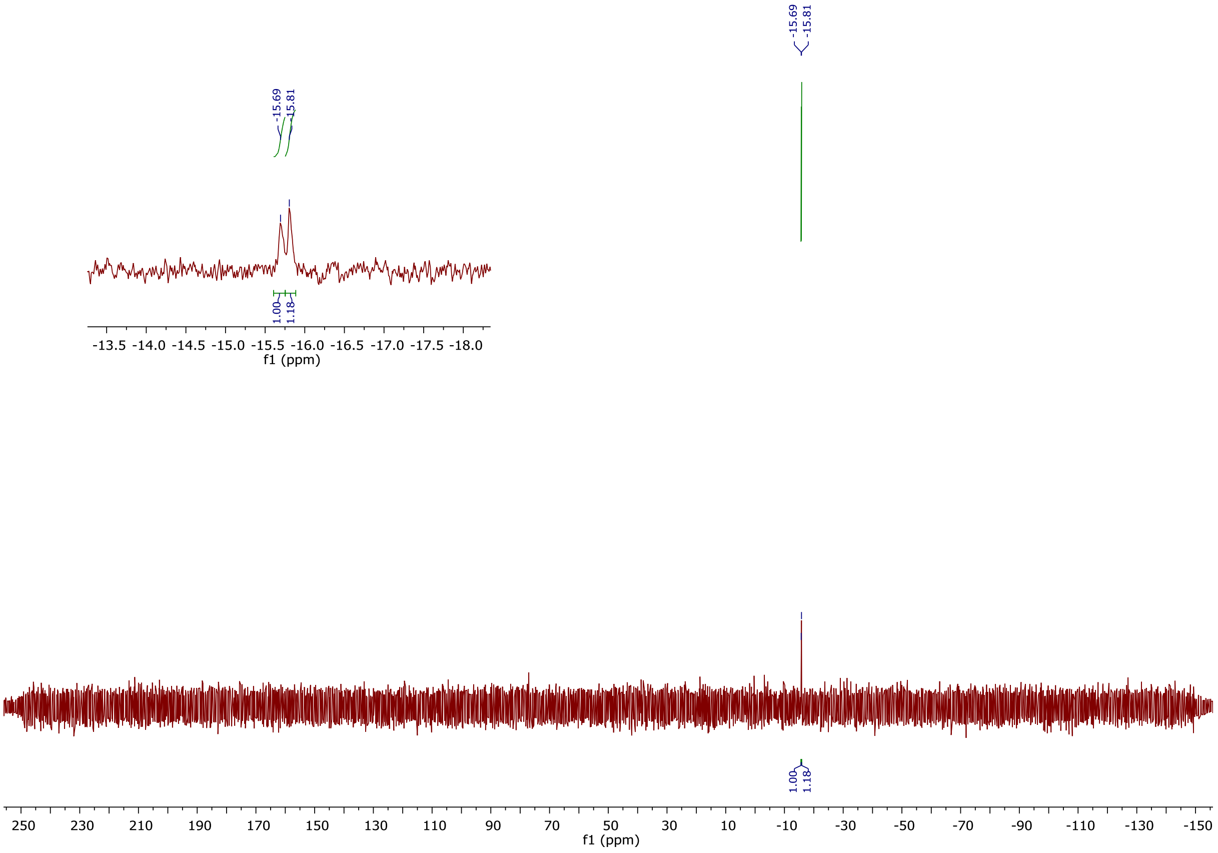

4.4.1 Ethynyl-PO-c(GK(N_3)RRRRRRRK(N_3)G-NH_2) (EDPO P6)

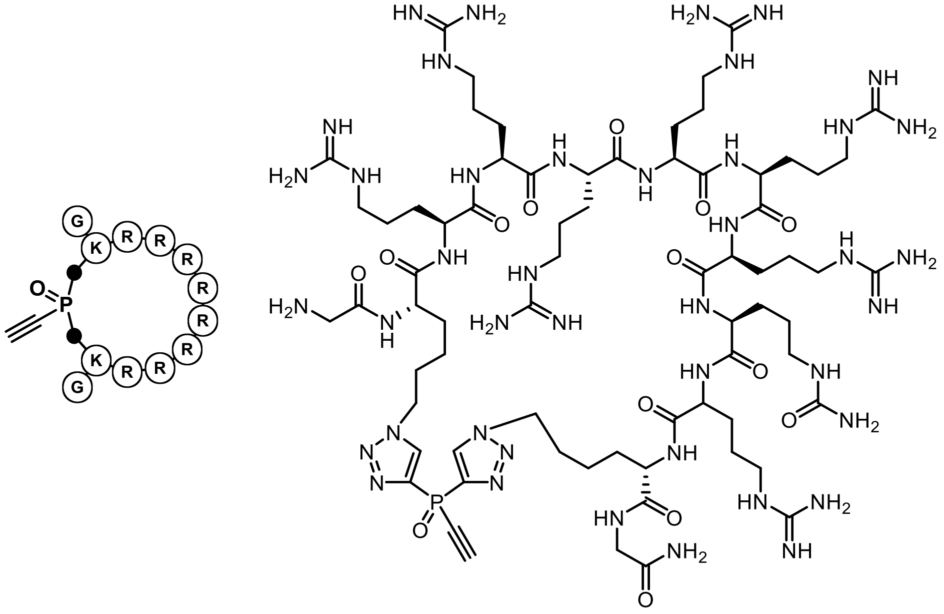

The peptide was cyclized according to general procedure but on a reduced 1.125 µmol scale (HPLC gradient 5-30% MeCN/Water) to give **P6** as an inseparable mixture of two diastereoisomers (0.91 mg, 0.31 µmol, 27%, dr 1:1.2). HRMS (ESI) for $C_{70}H_{128}N_{42}O_{14}P^{+}$ [M+2H^{+}] calc.: 906.0280, found 906.0320. ^{31}P NMR {^{1}H} (H_2O, 243 MHz) δ -15.66, -15.82.

UPLC UV trace (gradient D, 220 nm):

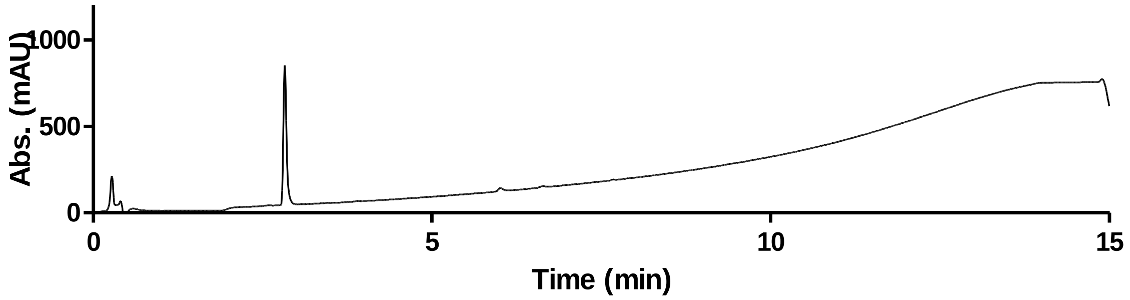

P6 – ^{31}P NMR {^{1}H} (243 MHz, H_2O)

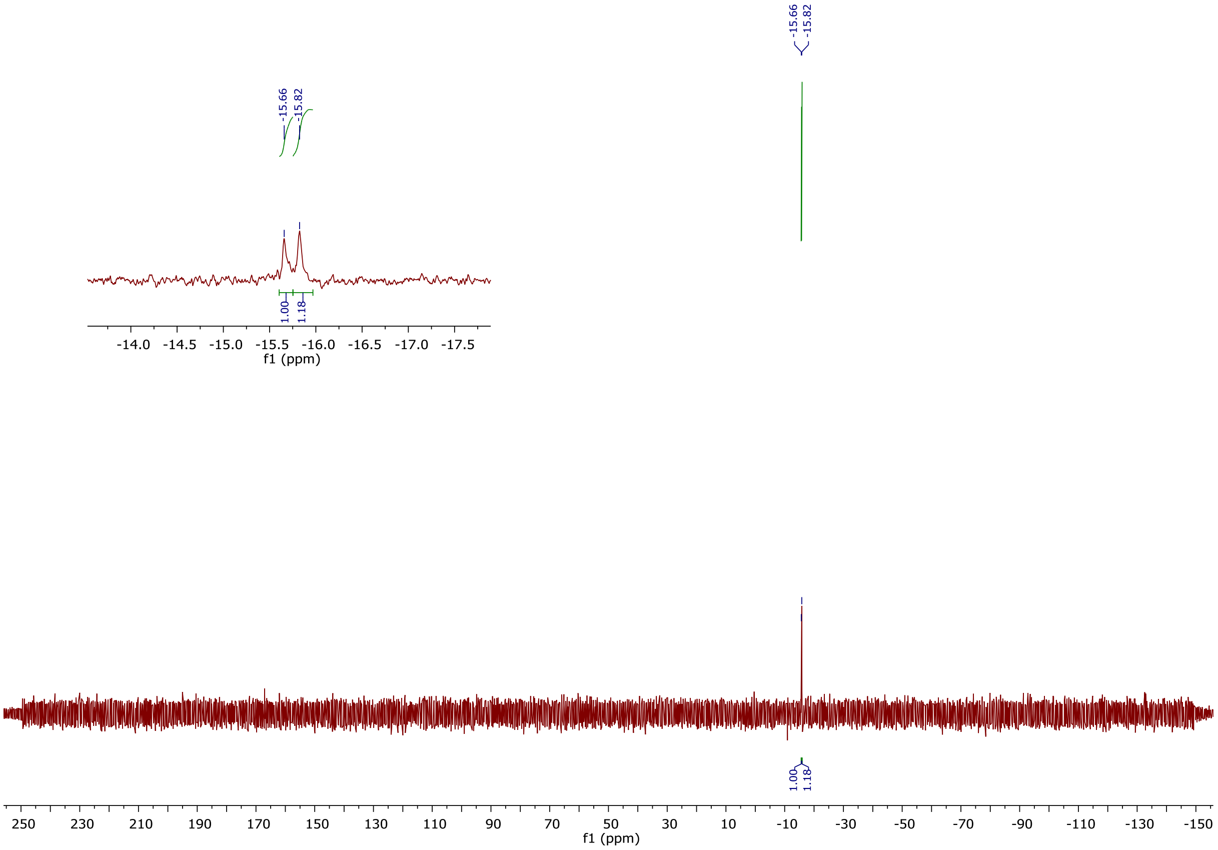

4.5 EDPO-Thiol conjugation:

4.5.1 Ac-LYRCAK-NH2- EDPO 4a

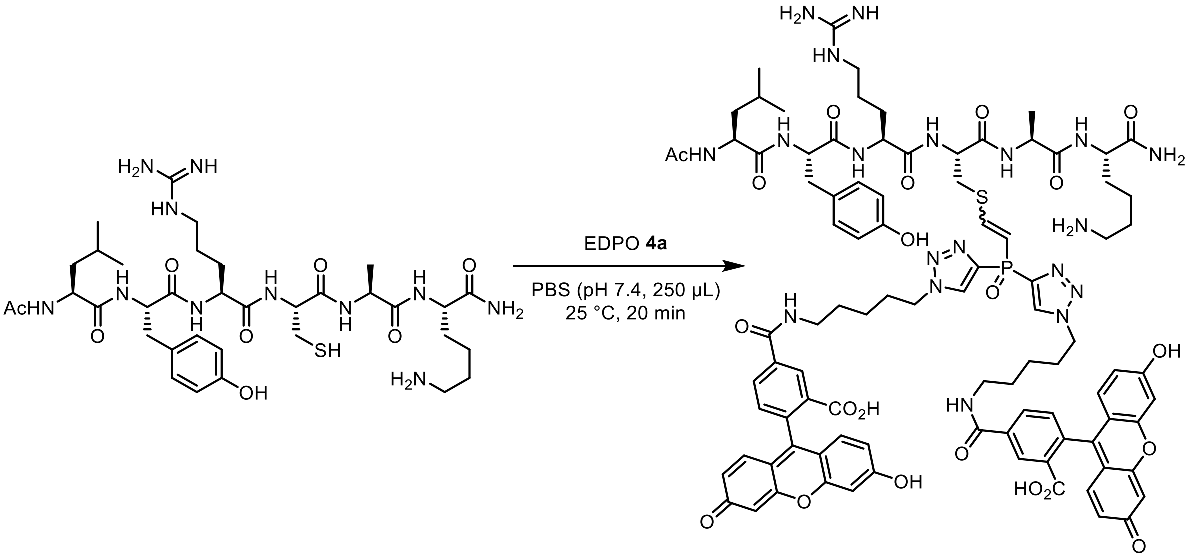

To a solution of Ac-LYRCAK-NH2 (2.5 µL of 100 mM stock in DMSO, 0.25 µmol, 1.0 equiv.) in PBS (pH 7.4, 250 µL) was added EDPO **4a** (30 µL of 10 mM stock in DMSO, 0.3 µmol, 1.2 equiv.). The reaction mixture was shaken at 25 °C for 20 minutes then purified by preparative HPLC (20-95% MeCN/Water) to give **LYRCAK-4a** (0.53 mg, 0.24 µmol, 97%). HRMS (ESI) for $C_{93}H_{107}N_{19}O_{21}PS^{+}$ $[M+2H]^{2+}$ calc.: 945.3724, found 945.3792.

UPLC UV trace (gradient D, 220 nm):

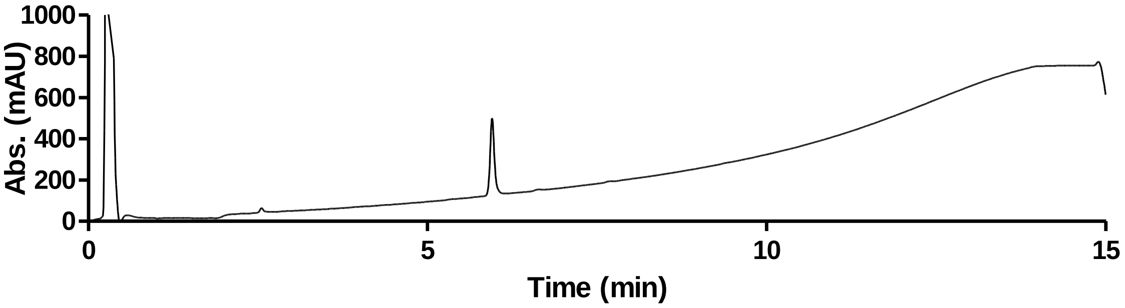

5 Uncropped Gels

5.1.1 **Uncropped Gel for figure 1c**

Coomassie stain:

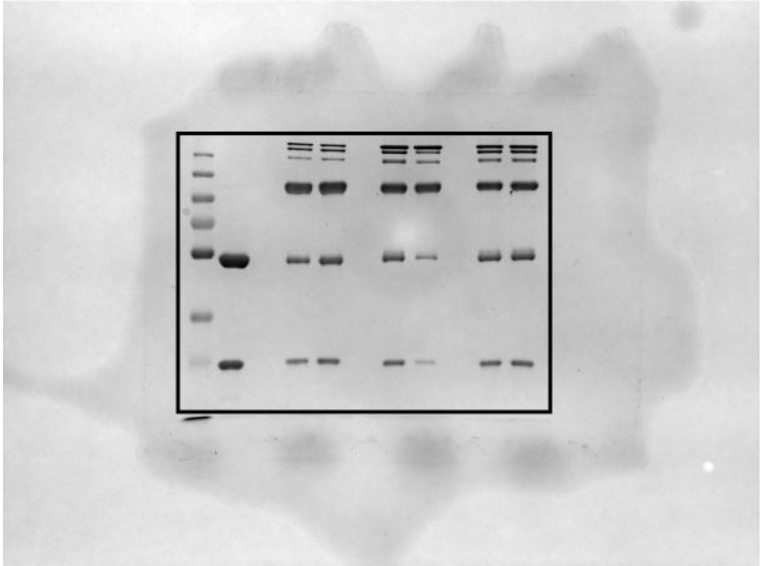

5.1.2 Uncropped Gel for figure 2c

Coomassie stain:

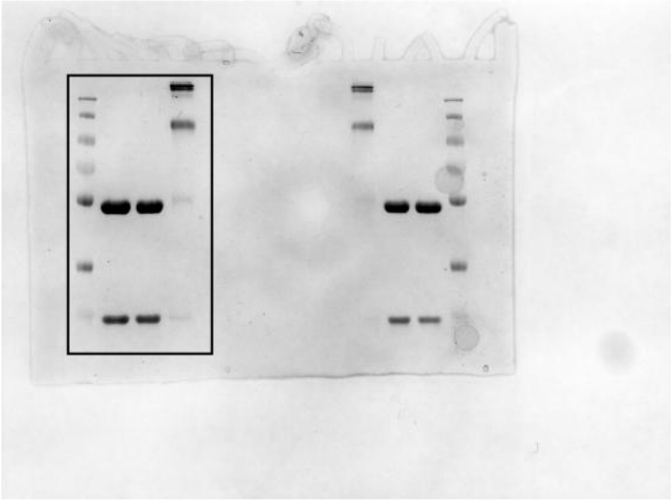

Fluorescent gel:

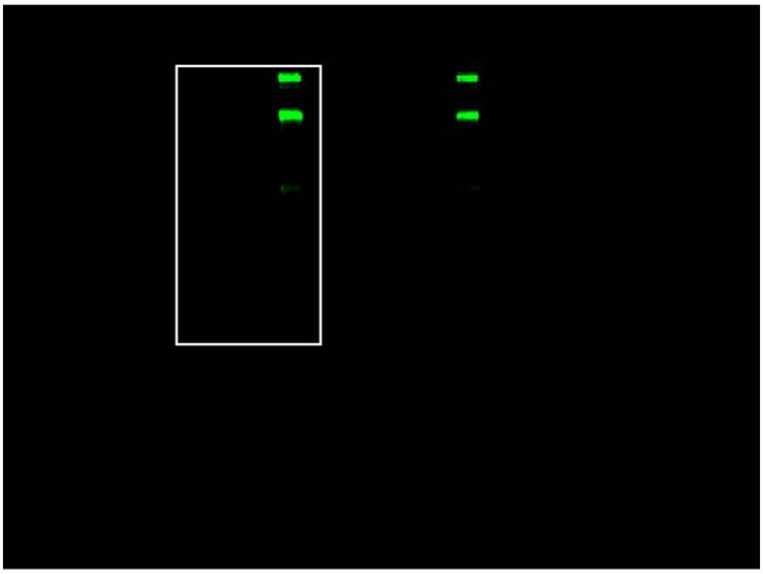

5.1.3 **Uncropped Gel for figure 2e**

Coomassie stain:

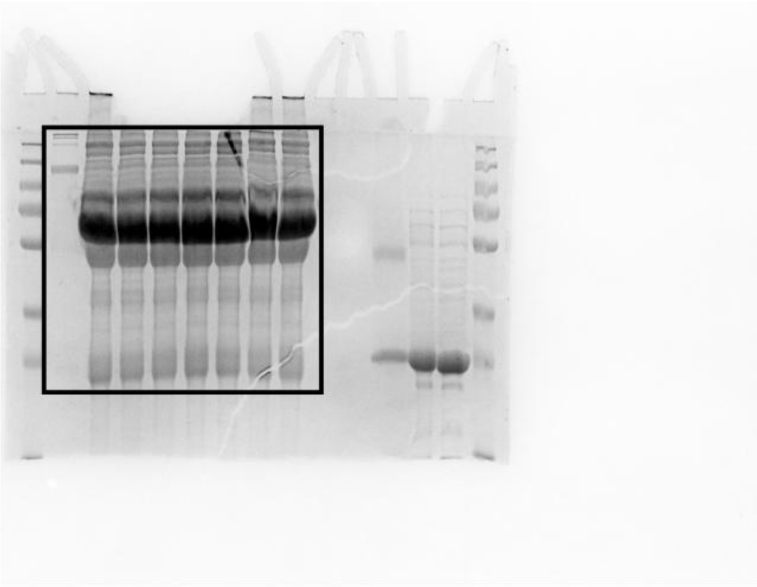

Fluorescent gel:

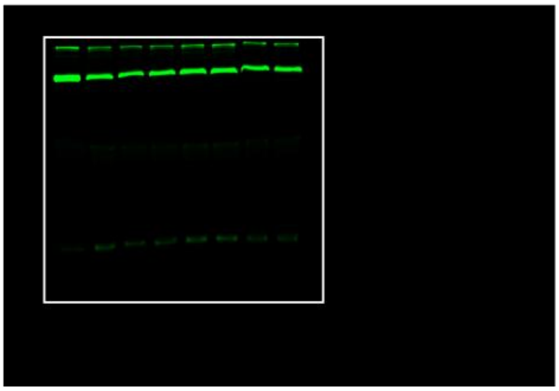

5.1.4 Uncropped Gel for figure S3

Coomassie stain:

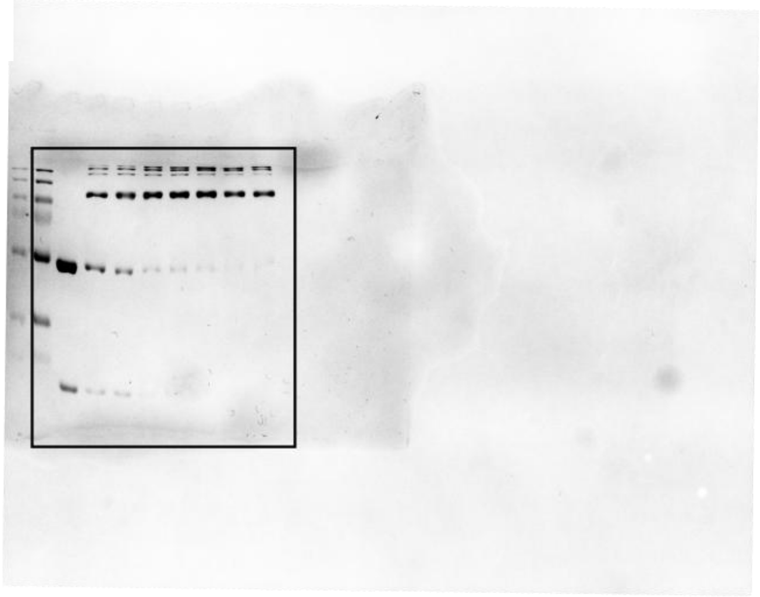

Fluorescent gel:

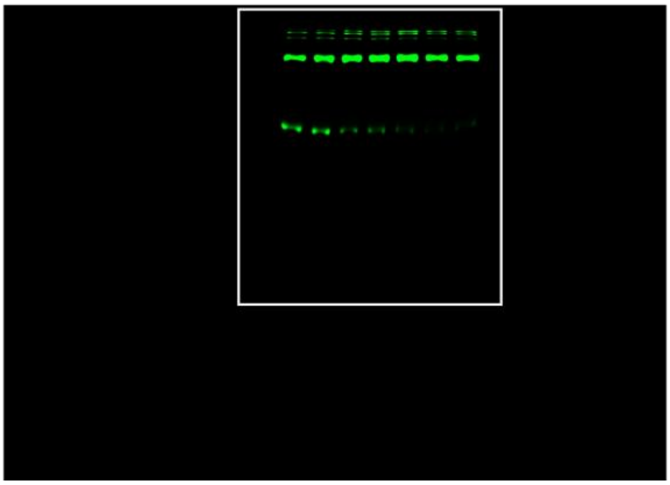

5.1.5 Uncropped Gel for figure S4

Coomassie stain:

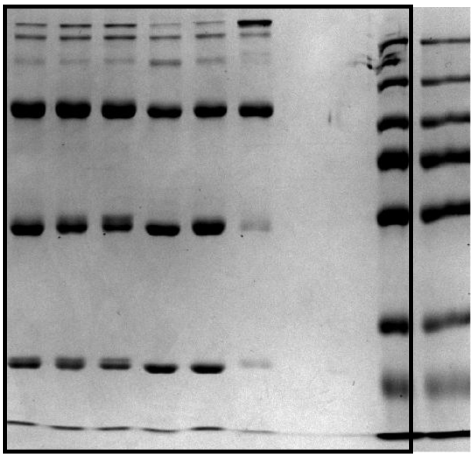

5.1.6 Uncropped Gel for figure S6

Coomassie stain:

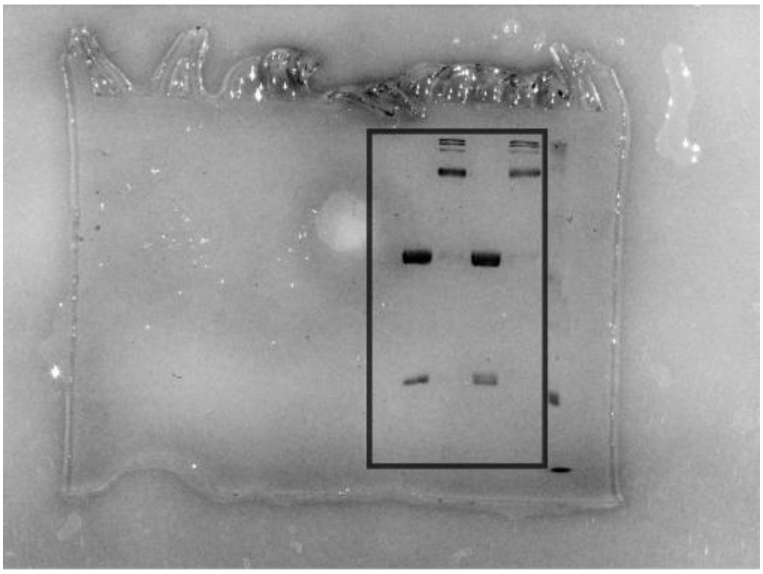

Fluorescent gel:

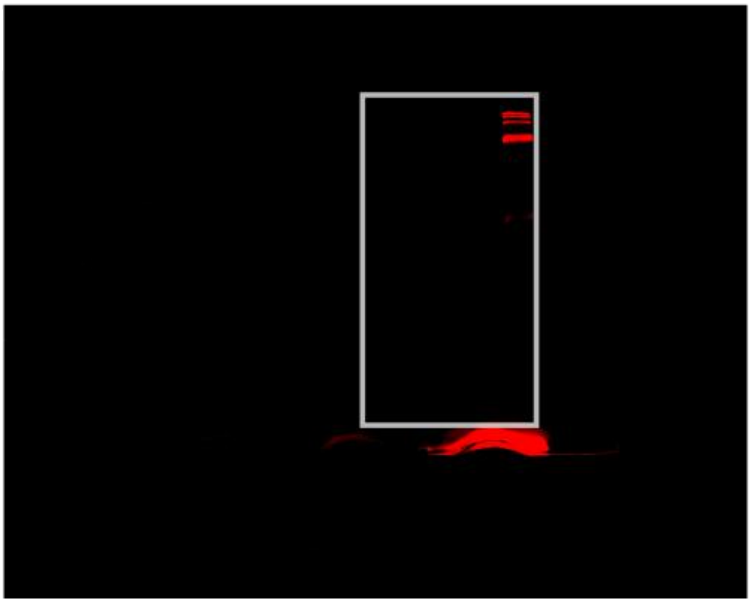

5.1.7 Uncropped Gel for figure S24a

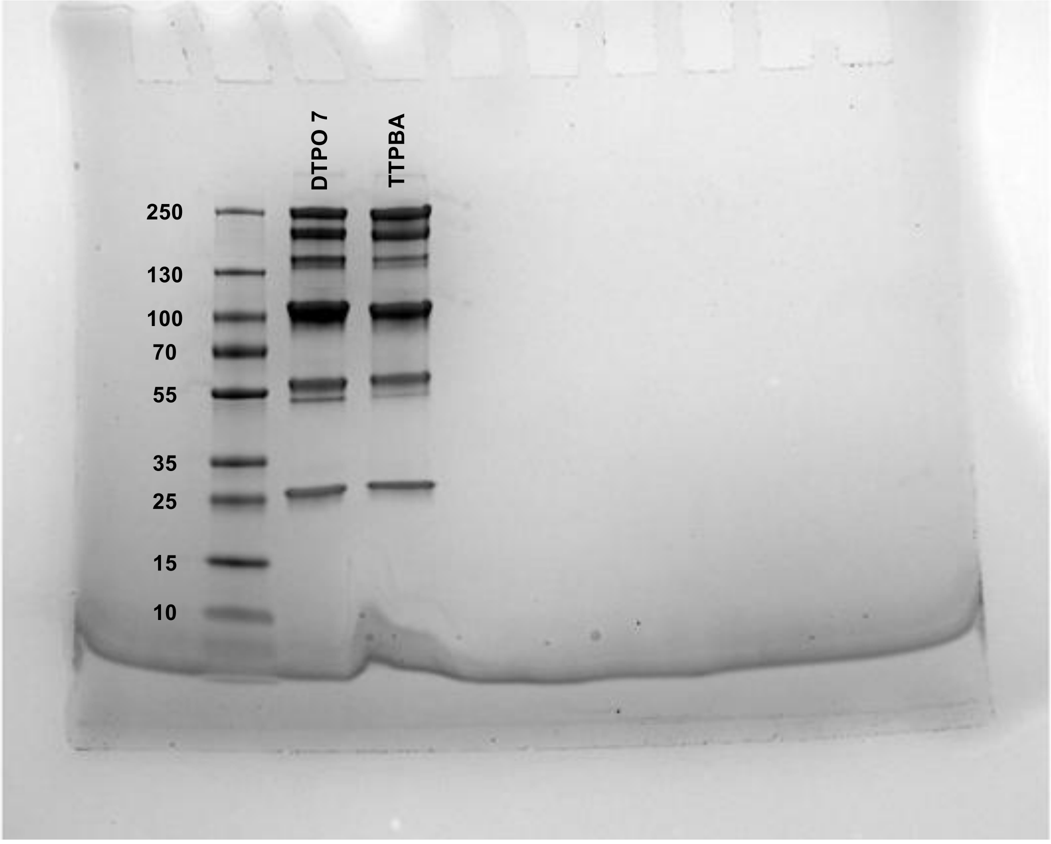

5.1.8 Uncropped Gel for figure S24b

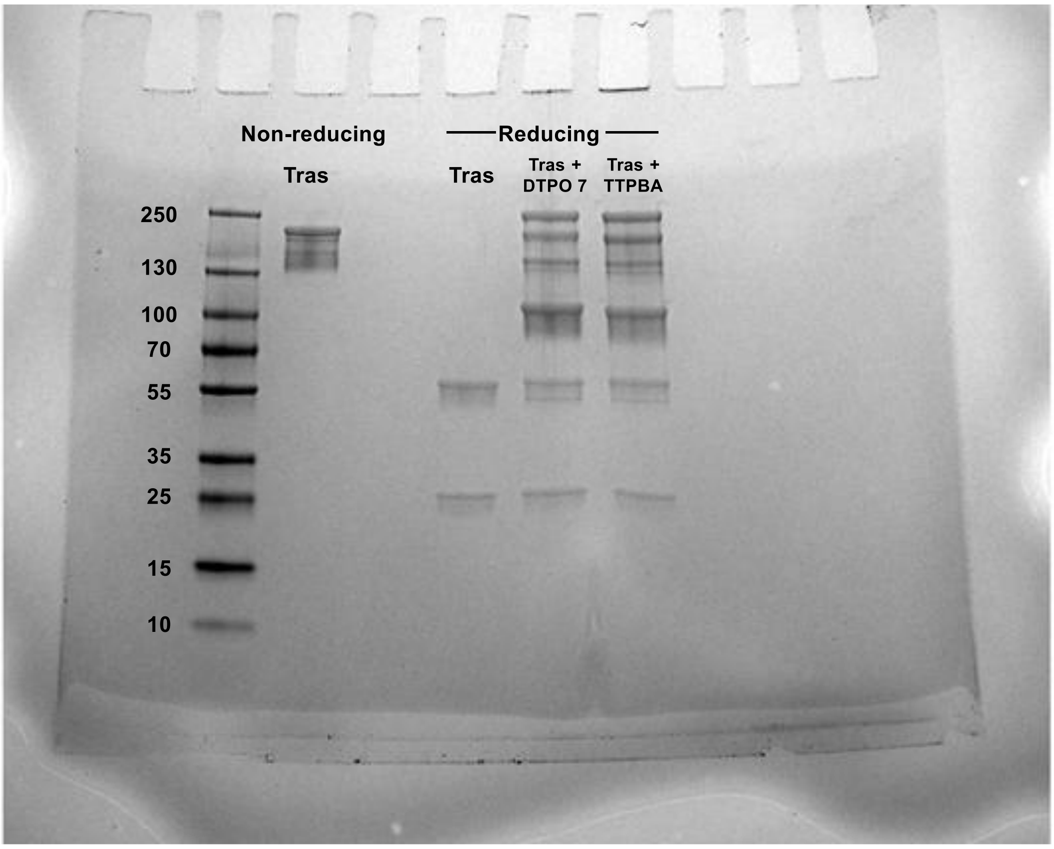

6 Supporting References

[1] M. C. Finniss, K. S. Chu, C. J. Bowerman, J. C. Luft, Z. A. Haroon, J. M. DeSimone, *Med. Chem. Commun.* **2014**, *5*, 1355-1358.

[2] P. Bryant, M. Pabst, G. Badescu, M. Bird, W. McDowell, E. Jamieson, J. Swierkosz, K. Jurlewicz, R. Tommasi, K. Henseleit, X. Sheng, N. Camper, A. Manin, K. Kozakowska, K. Peciak, E. Laurine, R. Grygorash, A. Kyle, D. Morris, V. Parekh, A. Abhilash, J. W. Choi, J. Edwards, M. Frigerio, M. P. Baker, A. Godwin, *Mol. Pharm.* **2015**, *12*, 1872-1879.

[3] F. Thoreau, L. N. C. Rochet, J. R. Baker, V. Chudasama, *Chem. Sci.* **2023**, *14*, 3752-3762.

[4] a) R. Benazza, I. Koutsopetras, V. Vaur, G. Chaubet, O. Hernandez-Alba, S. Cianferani, *Talanta* **2024**, *272*, 125727; b) L. Juen, C. B. Baltus, C. Gely, O. Feuillatre, A. Desgranges, M. C. Viaud-Massuard, C. Martin, *Bioconjug. Chem.* **2021**, *32*, 595-606; c) C. Bahou, D. A. Richards, A. Maruani, E. A. Love, F. Javaid, S. Caddick, J. R. Baker, V. Chudasama, *Org. Biomol. Chem.* **2018**, *16*, 1359-1366; d) F. F. Schumacher, J. P. Nunes, A. Maruani, V. Chudasama, M. E. Smith, K. A. Chester, J. R. Baker, S. Caddick, *Org. Biomol. Chem.* **2014**, *12*, 7261-7269.

[5] A. F. L. Schneider, M. Kithil, M. C. Cardoso, M. Lehmann, C. P. R. Hackenberger, *Nature Chemistry* **2021**, *13*, 530-539.

[6] A. L. Baumann, S. Schwagerus, K. Broi, K. Kemnitz-Hassanin, C. E. Stieger, N. Trieloff, P. Schmieder, C. P. R. Hackenberger, *J Am Chem Soc* **2020**, *142*, 9544-9552.

[7] W. Mao, W. Shi, J. Li, D. Su, X. Wang, L. Zhang, L. Pan, X. Wu, H. Wu, *Angew. Chem. Int. Ed.* **2019**, *58*, 1106-1109.

[8] C. E. Stieger, Y. Park, M. A. R. de Geus, D. Kim, C. Huhn, J. S. Slenczka, P. Ochtrop, J. M. Muchler, R. D. Sussmuth, J. Broichhagen, M. H. Baik, C. P. R. Hackenberger, *Angew. Chem. Int. Ed.* **2022**, *61*, e202205348.

[9] C. E. Stieger, L. Franz, F. Korlin, C. P. R. Hackenberger, *Angew. Chem. Int. Ed.* **2021**, *60*, 15359-15364.
